# Surmounting Recalcitrant Airway Inflammatory Disorders With a Multi‐Targeting Therapeutic Strategy by Tannic Acid‐Modified CuInP_2_S_6_ Nanosheets

**DOI:** 10.1002/EXP.20250073

**Published:** 2026-03-12

**Authors:** Ming Liu, Changyi Xu, Renqiang Ma, Xinran Xie, Ying Xie, Mengya Du, Zhaofeng Xu, Shuaiyin Zhang, Jian Li, Weiping Wen, Zhaoxu Tu

**Affiliations:** ^1^ Department of Otolaryngology The Sixth Affiliated Hospital Sun Yat‐sen University Guangzhou China; ^2^ Biomedical Innovation Center The Sixth Affiliated Hospital Sun Yat‐sen University Guangzhou China; ^3^ Key Laboratory of Human Microbiome and Chronic Diseases Ministry of Education Sun Yat‐sen University Guangzhou China; ^4^ Department of Clinical Laboratory The Sixth Affiliated Hospital Sun Yat‐sen University Guangzhou China; ^5^ Department of Otolaryngology The First Affiliated Hospital Sun Yat‐sen University Guangzhou China; ^6^ The Biomedical Translational Research Institute Key Laboratory of Viral Pathogenesis & Infection Prevention and Control School of Medicine Jinan University Guangzhou China; ^7^ Department of Biomedical Engineering Columbia University New York New York USA

**Keywords:** airway inflammation, bacterial infection, cell‐free DNA, CuInP_2_S_6_ nanosheets, reactive species, tannic acid

## Abstract

Recalcitrant airway inflammatory diseases have drawn significant attention due to their high incidence and substantial healthcare costs. Analysis of clinical samples from patients with chronic rhinosinusitis with nasal polyps (CRSwNP) revealed the coexistence of neutrophilic and eosinophilic inflammation, which may account for the limited efficacy of traditional single‐target therapeutic strategies. Moreover, cell‐free DNA (cfDNA)—an emerging inflammatory mediator—has been implicated in both eosinophilic and neutrophilic responses through its role in the formation of extracellular traps. In this study, we developed tannic acid (TA)‐modified CuInP_2_S_6_ (CIPS) nanosheets (C‐TA_1_; w/w = 1:1) as a multi‐targeting therapeutic nanoplatform for recalcitrant airway inflammatory diseases. The C‐TA_1_ nanosheets demonstrated efficient cfDNA clearance via hydrogen bonding interactions, thereby inhibiting cfDNA‐triggered toll‐like receptor 9 (TLR9) activation and subsequent nuclear factor‐κB (NF‐κB) inflammatory signaling. Additionally, C‐TA_1_ exhibited potent antioxidant and antibacterial activities, which were ascribed to the inherent properties of the two‐dimensional nanostructure and the chemical characteristics of TA, respectively. The in vivo therapeutic efficacy of C‐TA_1_ was evaluated in murine models with neutrophilic and eosinophilic airway inflammation, respectively. C‐TA_1_ markedly attenuated airway inflammation in both of these animal models by reducing reactive species, immune cell infiltration, goblet cell hyperplasia and the expression of pro‑inflammatory cytokines. Furthermore, treatment with C‐TA_1_ effectively modulated the dysregulated airway microbiota observed in the inflammatory state. Our findings demonstrate a multi‐targeting nanoformulation designed to mitigate multiple key pathological drivers of severe airway inflammation concurrently. This engineered system presents a promising strategy for managing respiratory inflammatory disorders and also other inflammation‐related diseases.

## Graphical abstract

Our findings underscore a multi‐targeting strategy for severe airway inflammation that multi‐targeting nanoformulation C‐TA_1_ markedly attenuated airway inflammation by (1) cfDNA elimination to alleviate neutrophilic/eosinophilic inflammation mediated by the TLR9 pathway, (2) prevention of RONS‐induced tissue injury and inflammatory response, (3) modulation of the airway microbiota through multi‐targeting therapeutic strategy.

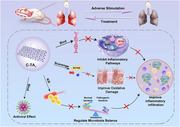

## 1 Introduction

Severe airway inflammatory diseases have attracted escalating attention, including chronic rhinosinusitis with nasal polyps (CRSwNP), asthma, acute lung injury (ALI), chronic obstructive pulmonary disease (COPD) etc., affecting millions of people worldwide [1, 2, 3, 4]. Therefore, it is crucial to develop novel therapeutic strategies that achieve satisfactory therapeutic outcomes while reducing systemic side effects. According to previous reports, CRSwNP and asthma are typically characterized by type 2 inflammation, dominated by eosinophil infiltration [5, 6]. However, recent studies proclaimed that the immune cascade in severe airway inflammatory diseases is complicated and often exhibits mixed inflammatory patterns involving both neutrophils and eosinophils [7, 8, 9, 10]. Although the role of eosinophils in recalcitrant airway inflammatory disorders has long been recognized, the study from Liu et al. disclosed that the degree of neutrophil infiltration is positively correlated to the severity of CRSwNP patients [11]. These findings collectively manifested the significance of targeting both neutrophilic/eosinophilic inflammation in the treatment of severe airway inflammation.

Cell‐free DNA (cfDNA) is extracellular DNA fragments released from injured body cells under external stimulus [12, 13, 14, 15], serving as a damage‐associated molecular pattern (DAMP) [16, 17] and acting as an agonist of toll‐like receptors (TLRs). cfDNA was reported to trigger a cascade of intracellular signaling, activate transcription factors, including nuclear factor kappa B (NF‐κB), and elicit the expression of inflammatory cytokines [18, 19, 20, 21]. The abnormal elevation of cfDNA was observed in many inflammatory diseases, and cfDNA scavenging by biomaterials has been successfully exploited for inflammation modulation [22, 23]. More importantly, it was revealed that cfDNA could trigger the degranulation of neutrophils and eosinophils via TLR9 activation and induce the formation of neutrophil extracellular traps (NETs) and eosinophil extracellular traps (EETs) [24], indicating that cfDNA is a promising therapeutic target for both neutrophilic and eosinophilic inflammation.

In addition, excessive generation of reactive oxygen and nitrogen species (RONS) also heavily participate in the progression of dysregulated inflammation [25, 26, 27]. The accumulation of RONS in pathological tissues induces DNA damage and cell apoptosis while boosting the expression of pro‐inflammatory factors. Moreover, reactive species compromise the integrity of the mucosal barrier, thereby facilitating pathogen invasion [28, 29, 30]. Complications of bacterial and viral infections further exacerbate airway inflammation [31], and the emergence of antibiotic‐resistant bacteria compounds these challenges [32, 33], hindering effective therapy. Pathogen infections also alter the composition of bacterial communities in the airway, and the microbiota imbalances can retard the resolution of inflammation [34, 35]. Consequently, developing multifunctional nanoplatforms with simultaneous cfDNA scavenging, antioxidant, and anti‐pathogen properties is extremely significant for the treatment of recalcitrant airway inflammatory disorders.

Two‐dimensional (2D) nanomaterials with a planar backbone and sheet‐like nanostructure provided new insights for the construction of therapeutic nanoformulations [36]. In recent years, CuInP_2_S_6_ (CIPS), as novel 2D nanosheets, has shown great potential in biomedical applications due to its large surface to volume ratio, exceptional molecular adsorption selectivity, and outstanding biocompatibility [37, 38]. Meanwhile, CIPS exhibits excellent SARS‐CoV‐2 receptor‐binding domain (RBD) adsorption ability and has become a promising candidate drug for antiviral therapy [39]. Tannic acid (TA) is a polyphenolic compound with considerable antioxidant capacity, which prevents free radical damage and enhances the activity of antioxidant enzymes. In addition, TA molecules can be expediently anchored onto the metal‐incorporated nanomaterials, owing to the metal–phenol network (MPN) and Fenton reactions between phenolic hydroxyls and metal atoms [40, 41, 42]. As a result, TA has been extensively adopted in the construction of functional nanoplatforms, including anti‐inflammatory and anti‐tumor nanomedicines [43, 44]. Moreover, TA modification is also expected to enhance the solubility and stability of nanosheets while improving in vivo bioavailability to promote therapeutic efficacy.

**SCHEME 1:**
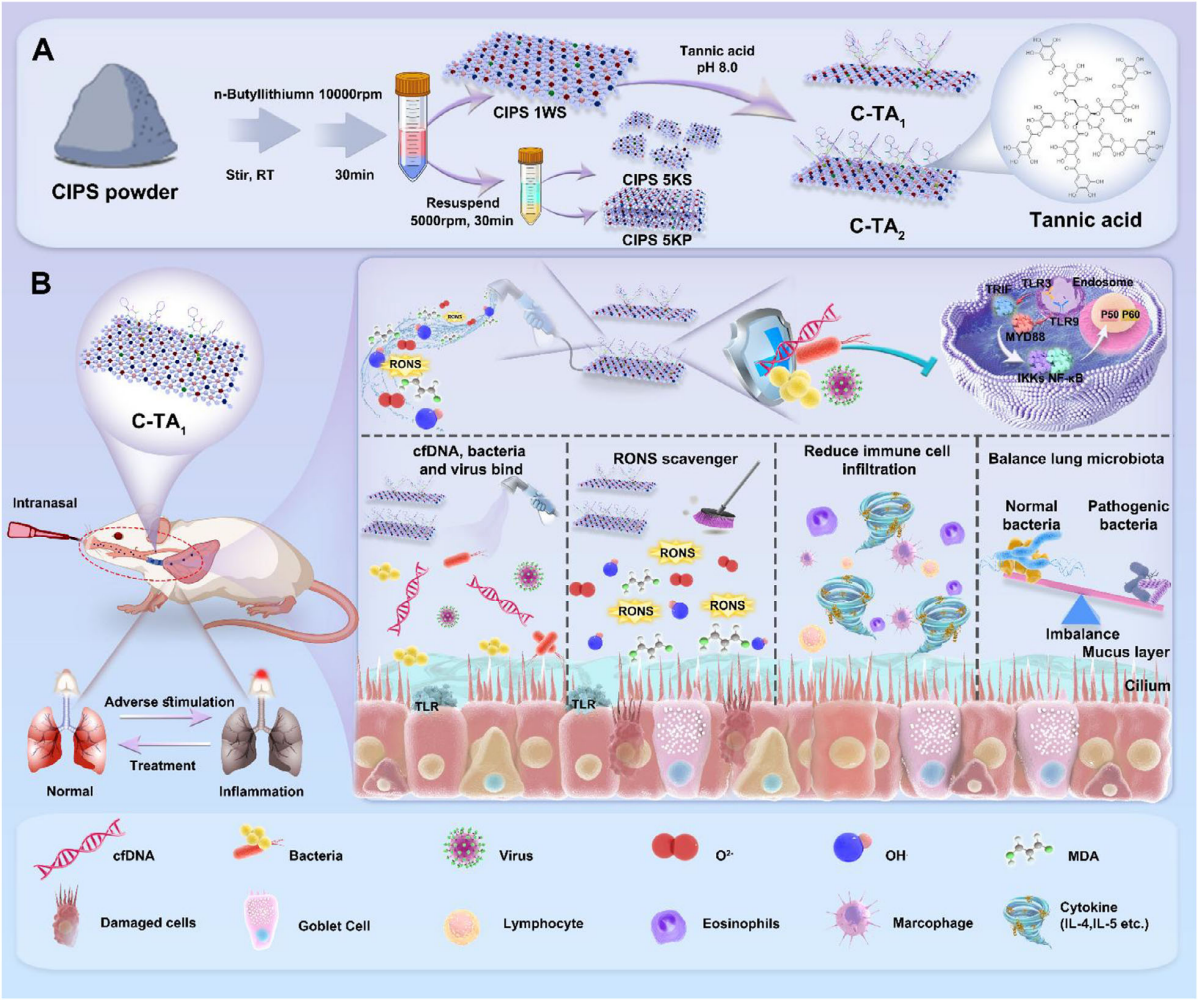
The preparation of TA‐covered CIPS nanosheets and their application for airway inflammation treatment. (A) The synthesis routes of C‐TA_1_ and C‐TA_2_. (B) C‐TA_1_ alleviated airway inflammation and restored airway microbiota in model mice through a multi‐targeting therapeutic strategy.

Here, we developed multifunctional TA‐covered CIPS nanosheets aimed at providing a feasible therapeutic strategy for recalcitrant airway inflammation characterized by mixed neutrophil/eosinophil infiltration and pathogen infection (Scheme 1). The CIPS monolayer nanosheets were exfoliated by the lithium‐ion intercalation method. And then, TA was strategically anchored on the surface of the nanosheets, forming functionalized TA‐covered CIPS nanosheets with different ratios (C‐TA_1_ and C‐TA_2_). The cfDNA binding, antioxidant, and anti‐pathogen capacities of multifunctional TA‐covered nanosheets were examined in detail. To comprehensively evaluate the in vivo therapeutic effects of functional nanosheets, both mouse models with LPS‐stimulated neutrophilic inflammation and OVA‐stimulated eosinophilic inflammation were established. In addition to examining the effects of inflammation modulation, the regulation of airway microbiota in pathological conditions was meticulously scrutinized by 16S rRNA gene sequencing.

## 2 Results and Discussion

### 2.1 Analysis of Nasal Polyps and Nasal Secretions of CRSwNP Patients

**FIGURE 1:**
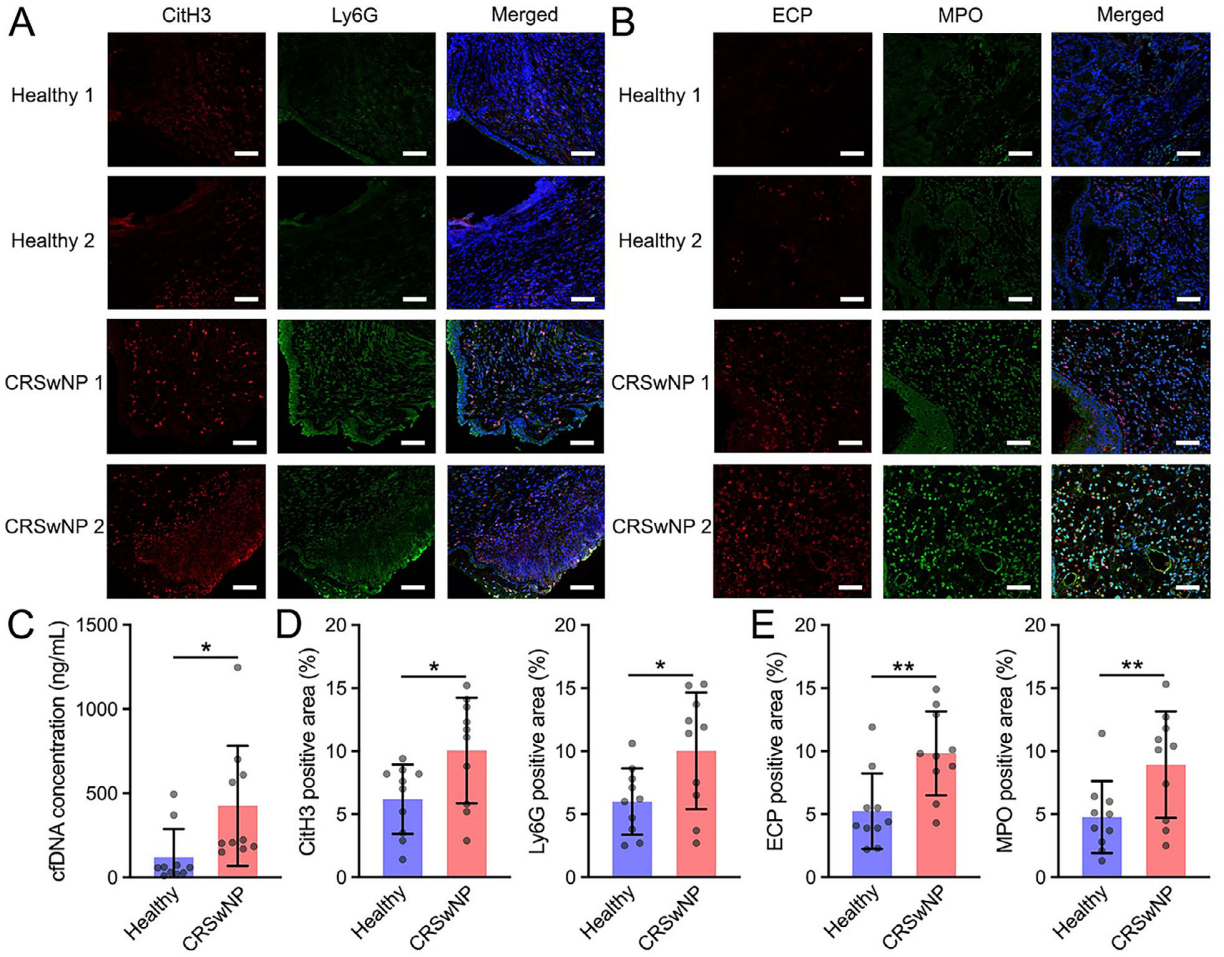
The NETs/EETs in nasal polyps of CRSwNP patients and the correlation with cfDNA level in nasal secretions. (A, B) Representative images of the nasal mucosa and nasal polyps from healthy volunteers and CRSwNP patients, respectively. DAPI staining and CitH3 and Ly6G immunostaining were adopted in the (A) group. DAPI staining and ECP and MPO immunostaining were applied in the (B) group. Scale bars: 20 µm. (C) The cfDNA concentration in the nasal secretions. (D) Quantification of the CitH3‐positive area (%) and Ly6G‐positive area (%) in the immunostaining images. (E) Quantification of the ECP‐positive area (%) and MPO‐positive area (%) in the immunostaining images. Data are presented as mean ± SEM (*n* = 10, *P < 0.05, **P < 0.01).

Although the coexistence of neutrophilic/eosinophilic inflammation was reported in some airway inflammatory diseases, the cfDNA and NETs/EETs in CRSwNP patients still need to be investigated. We collected nasal mucosa and nasal secretions from CRSwNP patients (*n* = 10) and healthy volunteers (*n* = 10). Subsequently, fluorescence staining was adopted to measure the immune cell infiltration in the nasal mucosa, and the pico‐green assay was utilized to determine the cfDNA level in nasal secretions. In detail, nasal mucosal slices were stained with citrullinated histone H3 (CitH3) and lymphocyte antigen 6G (Ly6G) antibodies to identify NETs (Figure 1A), as well as eosinophil cationic protein (ECP) and myeloperoxidase (MPO) antibodies to label EETs (Figure 1B). Representative images of the staining slices using confocal laser scanning microscopy (CLSM) depicted that the fluorescence from the above antibodies in the nasal polyps of CRSwNP patients was higher than that of healthy volunteers. Consistent with previous studies, cfDNA levels in the nasal secretions of CRSwNP patients were significantly higher than those in the nasal secretions of healthy volunteers (426.1±112.7 ng/mL vs. 120.7±53.1 ng/mL) (Figure 1C). Elevated CitH3^+^ and Ly6G^+^ regions in CLSM images (1.71‐fold and 1.67‐fold) showed that the formation of NETs in the nasal polyps of CRSwNP patients was higher than that in healthy volunteers (Figure 1D). Excessive EET generation in inflammatory tissues of CRSwNP patients was also revealed by the quantification of ECP^+^ and MPO^+^ regions (1.87‐fold and 1.99‐fold) (Figure 1E). Furthermore, the results were further validated through western blotting (WB) analysis, confirming that CitH3 expression was significantly higher in CRSwNP patients compared to healthy volunteers (Figure S2). Based on the analysis of clinical samples and previous publications, both neutrophilic and eosinophilic inflammatory pathways were involved in the dysregulated inflammation of CRSwNP patients. As cfDNA was reported to sensitize neutrophils and eosinophils via TLR‐9 activation and promote NET and EET formation [24, 45], cfDNA scavenging strategies with biomaterials were developed for the treatment of intractable inflammatory diseases.

However, the therapeutic efficacy of nanoplatforms targeting only cfDNA remains unsatisfactory due to persistent challenges, such as the presence of elevated RONS and bacterial infections in the injured nasal mucosa. In consequence, exploiting multifunctional nanosheets that can simultaneously address multiple inflammatory mediators is rewarding for the effective treatment of recalcitrant airway inflammation.

### 2.2 Preparation and Characterization of TA‐Covered CIPS Nanosheets

In this study, CIPS was selected and modified by the TA to increase the antioxidant function and biocompatibility of nanosheets. Firstly, we adopted lithium‐ion intercalation technology to exfoliate CIPS powder and investigated the properties of the products (Scheme 1A). It has been confirmed that the CIPS nanosheets in the supernatant after centrifuging at 10,000 rpm (CIPS 1WS) exhibited robust cfDNA binding capacity, mild cytotoxicity, and excellent antibacterial activity (Figures S3–S6). Then, we mixed the CIPS 1WS product with TA in ddH_2_O (pH = 8.0), and the weight ratios (w/w) of CIPS/TA were set as 1:0.1, 1:0.5, 1:1, 1:2, 1:3, 1:5, 1:10, and 1:25 (C‐TA_0.1_, C‐TA_0.5_, C‐TA_1_, C‐TA_2_, C‐TA_3_, C‐TA_5_, C‐TA_10_, and C‐TA_25_), respectively. It was found that TA modification barely changed the dimension of nanosheets; the size of CIPS, C‐TA_1_, C‐TA_2_, and C‐TA_25_ was 160.51 ± 1.08 nm, 189.60 ± 1.51 nm, 197.33 ± 1.76 nm, and 160.37 ± 5.42 nm, respectively. In contrast, the surficial charge was different for the TA‐covered nanosheets; the zeta potential of CIPS, C‐TA_1_, C‐TA_2_, and C‐TA_25_ was −60.65 ± 4.36 mV, −54.35 ± 3.55 mV, −45.19 ± 4.35 mV, and −37.91 ± 3.94 mV, respectively (Figure S7). In addition, along with the increase of TA modification, the antioxidant ability of nanosheets was enhanced (DPPH· elimination: from 66.26 % to 89.24%; ABTS^+^ elimination: from 48.00% to 69.87%) (Figure S8). On the contrary, the cfDNA binding and antibacterial capacities of nanosheets decreased with the promotion of TA ratios (cfDNA binding: from 91.31% to 71.29%) (Figures S9 and S10). Based on the above results, C‐TA_1_ and C‐TA_2_ were selected in the subsequent experiments, which were attributed to the simultaneous robust cfDNA binding, antioxidant, and antibacterial performances.

**FIGURE 2:**
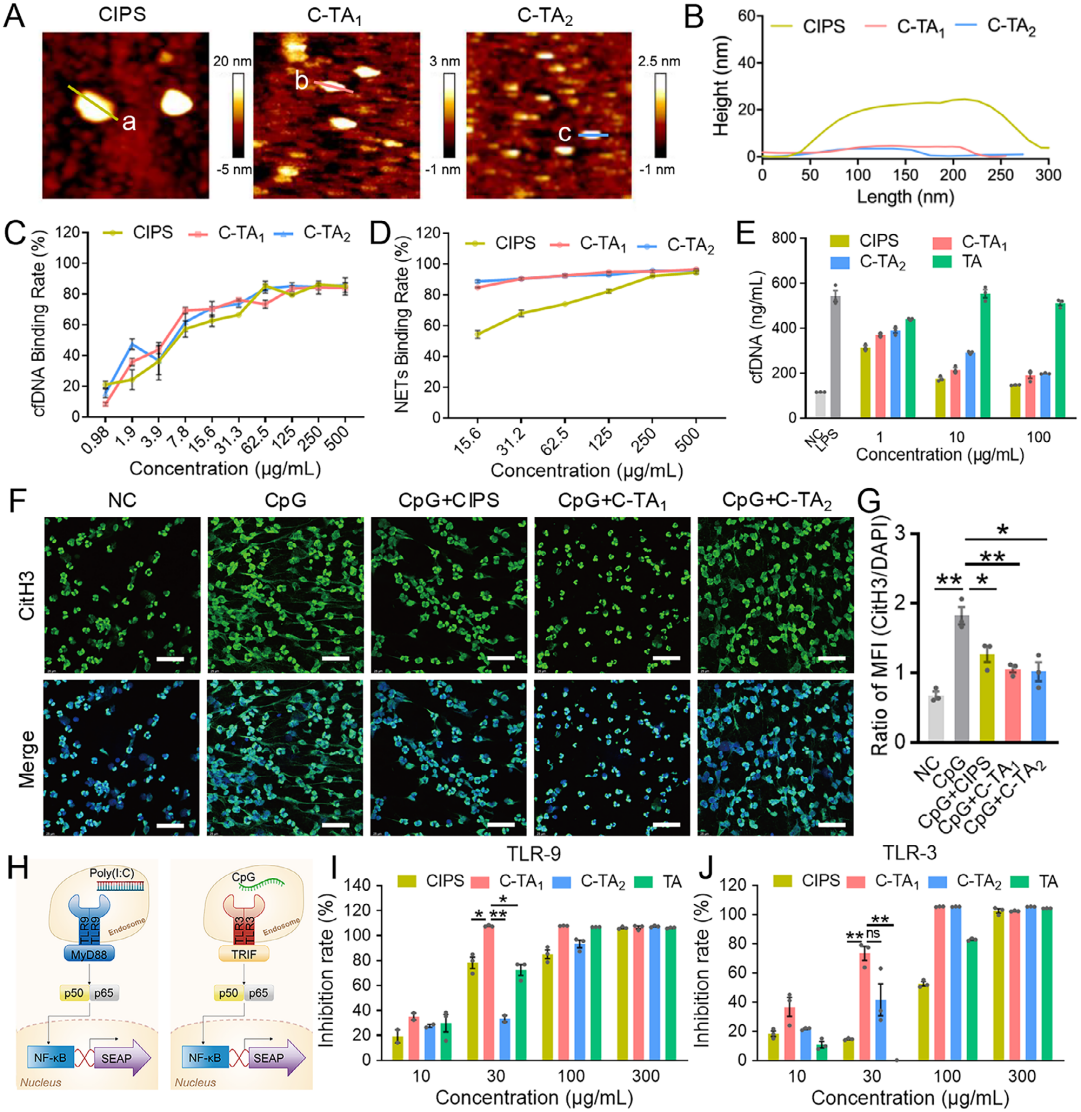
Multifunctional nanosheets inhibited TLR activation via cfDNA binding. (A) AFM images of CIPS, C‐TA_1_, and C‐TA_2_ nanosheets. (B) The height profiles of lines a–c in the AFM images. (C, D) cfDNA or NETs binding efficiency of CIPS, C‐TA_1_, and C‐TA_2_. (E) LPS‐stimulated cfDNA generation by BEAS‐2B cells after treatment with CIPS, C‐TA_1_, and C‐TA_2_. (F) Representative DAPI and CitH3 co‐staining images of the CpG‐treated neutrophils after incubation with CIPS, C‐TA_1_, and C‐TA_2_. Scale bars: 50 µm. (G) Quantitative analysis of fluorescence in NETs images. (H) Schematic of TLR activation assay. (I, J) TLR9 or TLR3 activation of HEK‐hTLR cells triggered by Ploy(I:C) or CpG after incubation with CIPS, C‐TA_1_, and C‐TA_2_. Data are presented as mean ± SEM (*n* = 3, Student's *t*‐test, two‐tailed, ns: no significant difference, *P < 0.05, **P < 0.01).

The 2D structural properties of CIPS, C‐TA_1_, and C‐TA_2_ were recorded using atomic force microscopy (AFM), with a thickness of 5 nm for C‐TA_1_ and C‐TA_2_ (Figure 2A,B). Interestingly, the height of CIPS was obviously higher than that of TA‐covered CIPS, indicating that TA modification was also beneficial for the full exfoliation of nanosheets. The transmission electron microscope (TEM) images also demonstrated their sheet‐like nanoscale morphology (Figure S11). According to the UV–vis absorption spectra results, the appearance of characteristic TA peak (275 nm) of C‐TA_1_ and C‐TA_2_ substantiated the successful preparation of TA‐covered nanosheets (Figure S12). The modification of TA on CIPS was also proved by Fourier transform infrared (FTIR) spectroscopy with the peaks at 755 cm^−1^ (Ph─C─H), 1313 cm^−1^ (Ph─O), and 1701 cm^−1^ (C═O), which are the characteristic absorption bands of TA (Figure S13). We examined the cytotoxicity of CIPS, C‐TA_1_, and C‐TA_2_ against BEAS‐2B and RPMI 2650 cells with the CCK8 assay. The outcomes showed that CIPS and C‐TA_1_ displayed almost no cytotoxicity after 24 h and 48 h incubation and even slightly promoted cell proliferation. Despite higher toxicity than C‐TA_1_, C‐TA_2_ scarcely descended cell growth when the concentration was below 100 µg/mL (Figure S14). In addition, hemolysis tests were conducted, and the hemolysis ratio induced by CIPS, C‐TA_1_, and C‐TA_2_ treatments (100 µg/mL) was less than 5% (Figure S15). Stability is a pivotal parameter of nanomaterials for biomedical applications, and the stable size of CIPS, C‐TA_1_, and C‐TA_2_ in ddH_2_O and 10% fetal bovine serum (FBS) at 4°C or at 37°C was recorded (Figure S16). These results indicated the successful preparation of TA‐modified CIPS, and the nanosheets with appropriate TA coverage (C‐TA_1_ and C‐TA_2_) were selected for the subsequent anti‐inflammatory studies.

### 2.3 cfDNA Binding Tests of Functional Nanosheets

Previous studies have reported that cfDNA plays crucial roles in the inflammatory cascade, such as TLR‐9 and cGAS‐STING pathways [21, 46, 47]. As TA was reported to adsorb dsDNA via hydrogen bonding [48], the cfDNA binding capacity of TA‐covered CIPS nanosheets was carefully examined. CIPS, C‐TA_1_, and C‐TA_2_ exhibited potent cfDNA binding ability even at a low concentration of 15.6 µg/mL, with rates of 62.8%, 70.4%, and 71.2%, respectively (Figure 2C). It was reported that cfDNA could activate neutrophils and trigger NETs formation, and dsDNA is also a principal component of NETs [24]. Therefore, TA‐covered nanosheets are likely to bind NETs and suppress NET‐elicited inflammatory pathways. As expected, the NETs binding ability of C‐TA_1_ and C‐TA_2_ was superior to CIPS (84.9% and 88.9% vs. 54.4%) (Figure 2D). To further explore the detailed interaction between TA‐modified nanosheets and cfDNA, agarose gel electrophoresis was performed on cfDNA incubated with CIPS, C‐TA_1_, and C‐TA_2_. Results revealed that cfDNA was bound to nanosheets via non‐covalent interactions, as the interaction could be disrupted by electrophoresis. Additionally, centrifugation of nanosheet‐cfDNA complexes resulted in their precipitation, with no detectable cfDNA in the supernatant, confirming the robust cfDNA adsorption (Figure S17). This indicates that the potent nanosheet‐cfDNA binding is promising to facilitate the clearance of cfDNA in the inflammatory tissues.

The results further demonstrated that C‐TA_1_ and C‐TA_2_ were able to scavenge cfDNA in the culture medium of LPS‐treated airway epithelial (Figure 2E and Figure S18). Visualization of NETs in vitro relies on their composition of histones, MPO, and neutrophil elastase [49], as NETs are web‐like structures composed of extracellular DNA fibers associated with proteins [50, 51]. The results showed that CIPS, C‐TA_1_, and C‐TA_2_ also significantly mitigated the NET generation of CpG‐treated neutrophils, which was calculated by the ratio of CitH3/DAPI fluorescence intensity (from 1.82 ± 0.12 to 1.27 ± 0.12, 1.06 ± 0.05, and 1.02 ± 0.13) (Figure 2F,G and Figure S19). We have validated NET generation using WB and Pico‐green assay. WB results showed that following CpG treatment, CitH3 expression increased in neutrophils, whereas treatment with C‐TA_1_ and C‐TA_2_ declined this upregulation (Figure S20A). In another experimental group, NETs were collected and quantified via PicoGreen detection of dsDNA. The results also confirmed CpG treatment significantly elevated NET level, which was reduced to the level of the control group after C‐TA_1_/C‐TA_2_ treatment (Figure S20B). It is generally known that TLR9 can recognize unmethylated CpG DNA, while TLR3 can recognize dsRNA, thereby activating the downstream inflammatory pathways [52]. We used HEK‐hTLR9 and HEK‐hTLR3 cells to simulate the TLR activation process (Figure 2H), and CIPS, C‐TA_1_, and C‐TA_2_ (30 µg/mL) treatment considerably modulated the activation of TLR3 and TLR9 (Figure 2I,J). These results collectively indicated that TA‐modified nanosheets inhibited the TLR activation via cfDNA/NETs scavenging, thereby suppressing the subsequent inflammatory cascade.

### 2.4 Antioxidant Efficiency of C‐TA_1_ and C‐TA_2_

**FIGURE 3:**
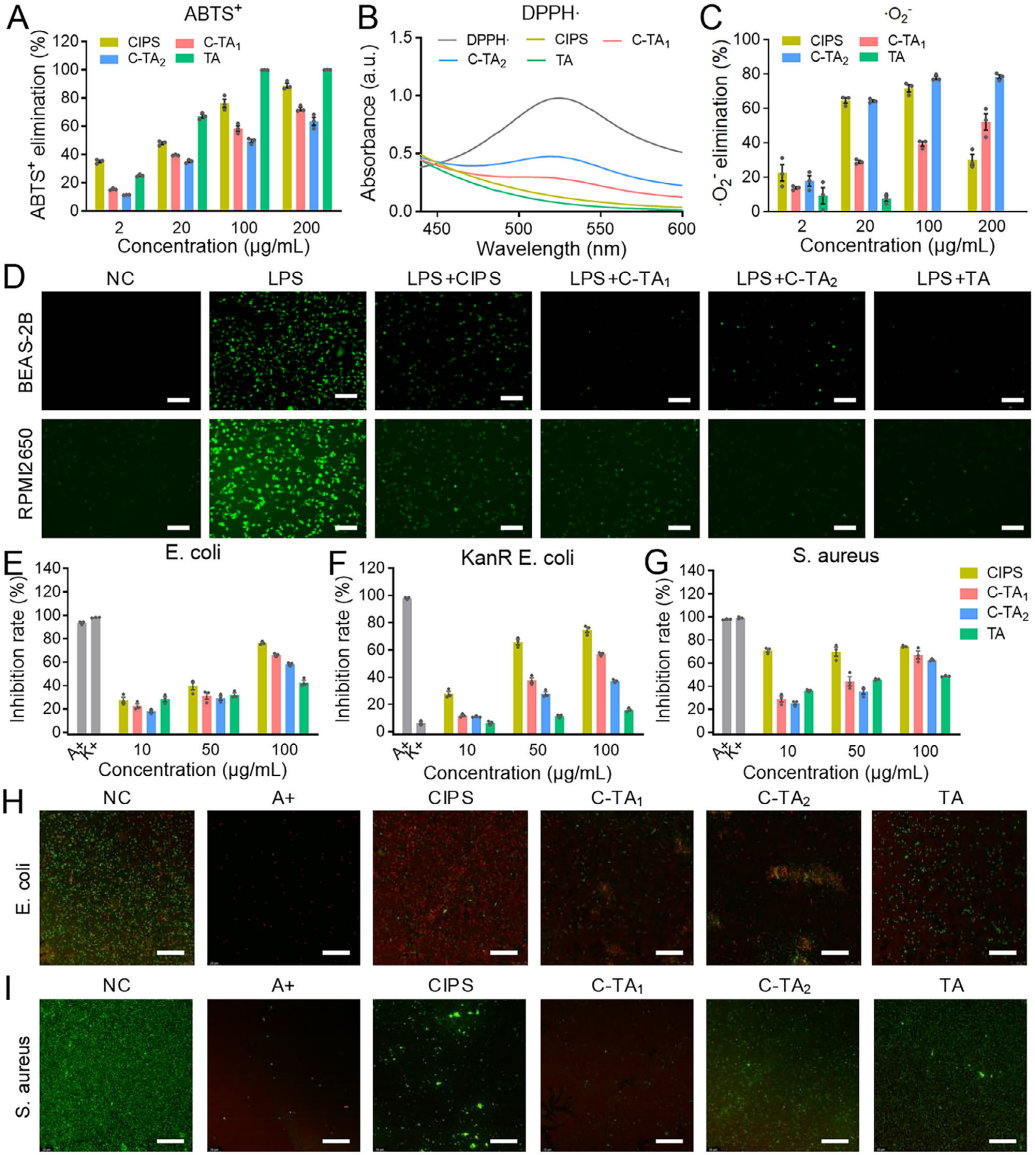
Antioxidant and anti‐bacterial efficiency of multifunctional nanosheets. (A–C) The antioxidant capacity of CIPS, C‐TA_1_, C‐TA_2_, and TA to (A) ABTS^+^, (B) DPPH∙, and (C) ∙O_2_^−^. (D) Representative DCFH‐DA fluorescent images of the LPS‐treated BEAS‐2B cells and RPMI2650 cells after incubation with CIPS, C‐TA_1_, C‐TA_2_, and TA. Scale bars: 200 µm. (E–G) The concentration of (E) *E. coli*, (F) KanR *E. coli*, and (G) *S. aureus* treated with CIPS, C‐TA_1_, C‐TA_2_, and TA. (H, I) Live/dead staining of (H) *E. coli* and (I) *S. aureus* treated with CIPS, C‐TA_1_, C‐TA_2_, and TA. Scale bars: 100 µm. Data are presented as mean ± SEM (*n* = 3).

In addition to cfDNA and NETs, the persistent RONS generation in the damaged airway tissues further aggravates the inflammation and delays the repair of injured tissues [26, 53]. Consequently, the antioxidant properties of nanomaterials are fascinating for the development of anti‐inflammatory nanoplatforms. ABTS^+^ and DPPH· are two classic free radical reagents and were adopted in this research to detect the redox capacities of nanomaterials. It was revealed that C‐TA_1_ and C‐TA_2_ (200 µg/mL) displayed robust ABTS^+^ (72.27% and 63.40%) and DPPH· (93.53% and 93.34%) reduction capacities (Figure 3A,B and Figure S21A). Furthermore, the ·O_2_^−^ and OH· could also be eliminated by C‐TA_1_ and C‐TA_2_, respectively (Figure 3C and Figure S21B). In addition, 2,7‐dichlorofluorescein diacetate (DCHF‐DA) was adopted as an indicator to detect intracellular ROS levels [54]. The quantification of fluorescence images depicted that CIPS, C‐TA_1_, C‐TA_2_, and TA effectively modulated LPS‐elicited intracellular ROS production (Figure 3D and Figures S22 and S23). Interestingly, the antioxidant capacities of C‐TA_1_ and C‐TA_2_ were superior to bare CIPS nanosheets in the above experiments, highlighting the critical role of TA in the therapeutic 2D nanoplatforms.

### 2.5 The Pathogen Inhibition by C‐TA_1_ and C‐TA_2_

In addition to the excessive cfDNA and RONS in the inflammatory nasal mucosa, patients with airway inflammation were commonly accompanied by pathogen infection. More seriously, antibiotic abuse leads to the emergence of drug‐resistant bacteria, and antivirus treatment is ineffective in some special situations, such as COVID‐19 [32, 33]. Therefore, the broad‐spectrum anti‐pathogen ability of nanomaterials is extremely beneficial for the treatment of airway inflammatory disorders. Fortunately, CIPS has been reported to mitigate COVID‐19 infection in model animals [39], and the 2D nanostructure is also promising for impeding bacterial growth via physical contact.

We investigated the bactericidal ability of CIPS, C‐TA_1_, C‐TA_2_, and TA using Gram‐negative bacteria *E. coli* and Gram‐positive bacteria *S. aureus*. Furthermore, ampicillin‐resistant *E. coli* (AmpR *E. coli*) and kanamycin‐resistant *E. coli* (KanR *E. coli*) were also adopted for antibacterial studies. Fascinatingly, these nanosheets impeded the growth of broad‐spectrum bacteria in a dose‐dependent manner, including antibiotic‐resistant strains. For example, the inhibitory efficacy of C‐TA_1_ (100 µg/mL) on *E. coli*, AmpR *E. coli*, KanR *E. coli*, and *S. aureus* was 66.14%, 68.27%, 57.07%, and 66.96%, respectively (Figure 3E–G and Figure S24). The agar plate colony counting assay also showed that CIPS, C‐TA_1_, and C‐TA_2_ extremely retarded the formation of bacterial colonies compared to the NC group (Figures S25–S28). In addition, the live/dead staining images confirmed that CIPS, C‐TA_1_, and C‐TA_2_ treatments resulted in significant bacterial deactivation, with stronger red (PI) fluorescence than the NC group (Figure 3H,I and Figures S29–S33). 2D nanosheets with a high surface to volume ratio can encapsulate bacteria, isolating them from the external environment and depriving them of nutrients, thereby inducing death [55]. To further investigate the interaction between TA‐modified nanosheets and bacteria, scanning electron microscopy (SEM) was performed on *E. coli* treated with CIPS, C‐TA_1_, and C‐TA_2_. SEM images confirmed that incubation with CIPS, C‐TA_1_, and C‐TA_2_ disrupted bacterial morphology, with the nanosheets adhering to the bacterial surface (Figure S34). These outcomes suggested that the TA modification in C‐TA_1_, and C‐TA_2_ scarcely declined the bactericidal capacity of CIPS and exhibited robust antibacterial ability in vitro.

The COVID‐19 pandemic caused a large number of deaths worldwide, and viral pneumonia has brought serious threats to human beings every several decades. It was reported that CIPS proclaimed relatively high selective binding loading for the receptor binding domain of spike proteins of wild‐type (WT) SARS‐CoV‐2 and its variants of interest (including Delta and Omicron), thereby impeding viral infection of the host and exerting antiviral effects [39]. In this study, we simulated virus infection in cells using a pseudo‐virus neutralization test, and the consequences substantiated the valid inhibition for SARS‐CoV‐2 and the BA.5 variant strain by TA‐covered CIPS nanosheets (Figure S35). Interestingly, CIPS displayed better therapeutic effects in the early stages of viral infection, which is determined by its antiviral properties through specific binding (Figure S36). These results collectively indicated that C‐TA_1_ and C‐TA_2_ retained the antiviral effect of CIPS, particularly against the currently prevalent strain, BA.5.

### 2.6 Biodistribution of C‐TA_1_ and C‐TA_2_

Despite potent cfDNA/NETs binding efficacy, along with antioxidant and antipathogenic properties, the in vivo biosafety and biodistribution of TA‐covered CIPS nanosheets need to be comprehensively evaluated before further therapeutic purposes. After 4 consecutive days of intranasal administration of nanosheets to healthy mice, important organs (heart, liver, spleen, lungs, and kidneys) and the peripheral blood of mice were collected. H&E staining images showed that CIPS, C‐TA_1_, and C‐TA_2_ treatments scarcely cause evident pathological damage to these organs (Figure S37). In addition, barely significant differences were observed in levels of aspartate aminotransferase (AST), alanine aminotransferase (ALT), creatinine (CREA), urea (URE), and creatine kinase (CK) (Figure S38), indicating the hepatic and renal functions were not injured by nanosheets. These outcomes jointly unraveled the sterling biocompatibilities of TA‐covered CIPS nanosheets and highlighted the significance of exploring their biomedical applications.

**FIGURE 4:**
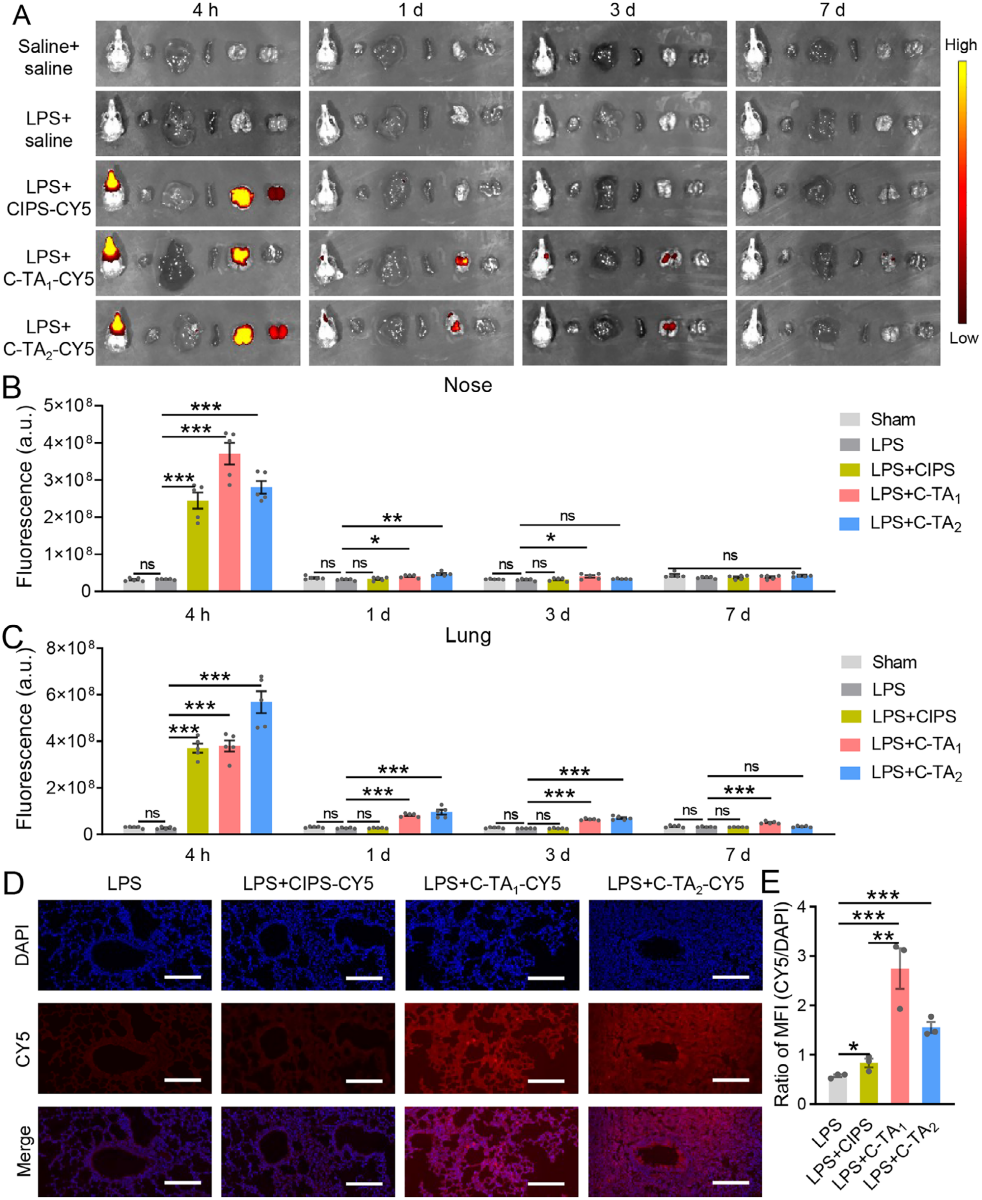
Biodistribution of multifunctional nanosheets. (A) Ex vivo fluorescence imaging of MBs and major organs (heart, liver, spleen, lungs, and kidneys in sequence) at 4 h, 1 day, 3 days, and 7 days after intranasal administration of CY5‐labeled nanosheets. (B, C) Quantification of fluorescence intensity from isolated (B) noses and (C) lungs from experimental mice. (D) Representative DAPI and CY5 co‐staining images of the lungs at 1 day after instillation. (E) Quantitative analysis of fluorescence intensity in fluorescent images of lungs. Scale bars: 200 µm. Data are presented as mean ± SEM (*n* = 3, Student's *t*‐test, two‐tailed, ns: no significant difference, *P < 0.05, **P < 0.01, ***P < 0.001).

To investigate the biodistribution of functional nanosheets after intranasal administration, we first prepared Cy5‐labeled CIPS, C‐TA_1_, and C‐TA_2_ (designated as CIPS‐CY5, C‐TA_1_‐CY5, and C‐TA_2_‐CY5, respectively). Subsequently, an LPS‐induced mouse model was established and intranasally administered with CY5‐labeled nanosheets. In vivo fluorescence recording at different time points (4 h, 1 days, 3 days, and 7 days) revealed that the nanosheets were rapidly accumulated in the nose and lung after local instillation. After 1 day, CIPS was almost undetectable, while the fluorescent signals from C‐TA_1_ and C‐TA_2_ were still observed in the respiratory systems. 7 days later, CIPS, C‐TA_1_, and C‐TA_2_ were fully degraded and excreted from the body (Figure 4A–C). It can be seen that TA modification delayed the biodegradation of CIPS and promoted its accumulation in the inflammatory lung tissues. More importantly, weak fluorescence signals were detected in hearts, livers, spleens, kidneys, and blood at 4 h after administration, indicating that only a trace of nanomaterials entered the circulation system through capillaries and were quickly degraded and excreted by the livers and kidneys (Figure S39). Finally, the ex vivo fluorescence images showed that C‐TA_1_ and C‐TA_2_ exhibited more significant accumulation in the lungs (Figure 4D,E). These studies jointly depicted that TA coverage contributed to the penetration and retention of nanosheets in inflammatory sites in vivo, which is beneficial for the treatment of airway diseases.

### 2.7 Functional Nanosheets Alleviated Neutrophilic Airway Inflammation

The analysis of clinical samples revealed that both neutrophilic and eosinophilic inflammation were observed in the nasal polyps of CRSwNP patients (Figure 1). However, there is still no well‐established mouse model with simultaneous neutrophilic/eosinophilic inflammatory characteristics. In regard to this, LPS‐stimulated model mice with neutrophilic airway inflammation and OVA‐stimulated model mice with eosinophilic airway inflammation were adopted to evaluate the therapeutic effects of functional nanosheets, respectively.

**FIGURE 5:**
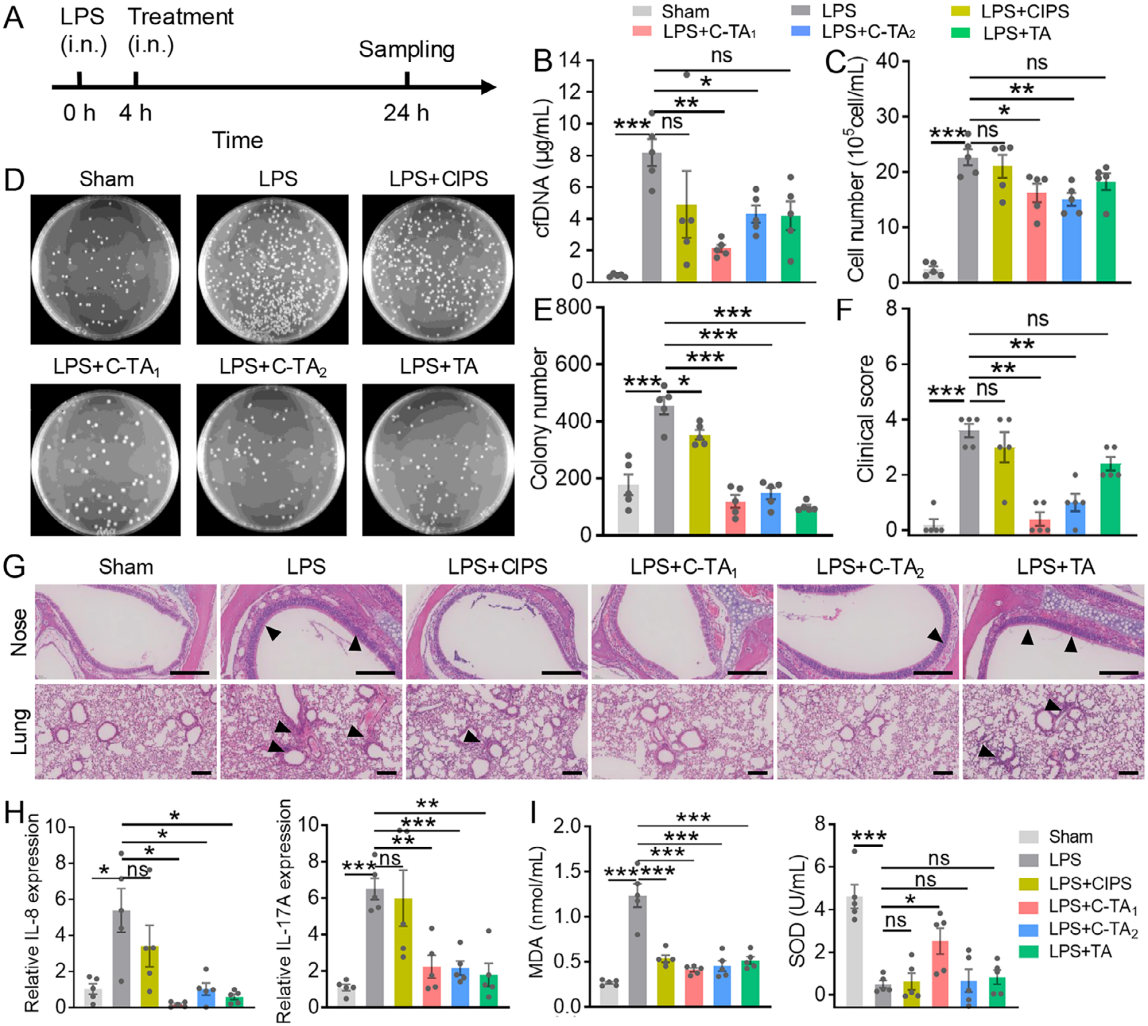
Multifunctional nanosheets modulated LPS‐stimulated neutrophilic airway inflammation. (A) Schematic diagram of the LPS‐stimulated mouse model and treatment strategy. (B) cfDNA concentration in the BALF of experimental mice. (C) Cell counting in the BALF of mice in different treatment groups. (D) Images of bacteria in the BALF after incubation in the plate for 12 h. (E) Count of colonies in bacterial images. (F) Clinical scores of experimental mice based on the H&E staining images of lung slices. (G) Representative H&E staining of the noses and the lungs isolated from the experimental mice. Scale bars: 200 µm. (H) RT‐qPCR results of IL‐8 and IL‐17A expressions in the lungs of mice in each group. (I) MDA and SOD levels in BALF of mice in each group. Data are presented as mean ± SEM (*n* = 5, Student's *t*‐test, two‐tailed, ns: no significant difference, *P < 0.05, **P < 0.01, ***P < 0.001).

For the study with neutrophilic inflammation [56], LPS was intranasally instilled in the mice; then, the mice were treated with CIPS, C‐TA_1_, C‐TA_2_, and TA after 4 h. The mice were raised for another 24 h before being sacrificed, and the maxillary bones (MBs) and lungs were collected (Figure 5A). Compared to the sham group, cfDNA levels in the bronchoalveolar lavage fluid (BALF) of LPS‐treated mice increased more than tenfold, and a similar trend was observed in the total cell count of BALF, confirming the LPS‐stimulated airway inflammation in model mice. As expected, C‐TA_1_ and C‐TA_2_ treatments markedly reduced cfDNA levels in BALF from 8.18 ± 0.85 µg/mL to 2.14 ± 0.23 µg/mL, and 4.29 ± 0.55 µg/mL, respectively (Figure 5B) and the decline of total cell count in BALF was also calculated (Figure 5C). At the same time, the agar plate colony counting assay was measured by dilution plate test with BALF, and the monoclonal formation in the nanosheets‐treated mice was significantly dampened and restored to nearly healthy levels, particularly in the C‐TA_1_, and C‐TA_2_ groups (Figure 5D,E).

To further evaluate the anti‐inflammatory effects of functional nanosheets, hematoxylin and eosin (H&E) staining was adopted, and the quantification results demonstrated that the infiltration of inflammatory cells was alleviated, and clinical scores were improved after treatment (Figure 5F,G). The periodic acid‐Schiff (PAS) staining images uncovered that LPS‐stimulated mucus layer thickening was ameliorated after treatment, suggesting nanosheets are promising to suppress excessive mucus production in patients with chronic airway disease (Figure S40). We also quantified the expression of cytokines (such as IL‐8 and IL‐17A) in the lungs of mice by RT‐qPCR, and the treatments greatly relieved the expression of inflammatory factors, especially in the C‐TA_1_ group (Figure 5H). Additionally, the in vivo antioxidant effects of TA‐covered CIPS nanosheets were further evaluated in the BALF of experimental mice. The results showed that C‐TA_1_ and C‐TA_2_ treatments considerably descended the content of malondialdehyde (MDA) and restored the level of superoxide dismutase (SOD), which is consistent with previous antioxidant tests (Figure 5I). These experiments collectively manifested that TA‐modified CIPS nanosheets (C‐TA_1_ and C‐TA_2_) could ameliorate LPS‐induced neutrophilic airway inflammation.

### 2.8 Functional Nanosheets Alleviated Eosinophilic Airway Inflammation

**FIGURE 6:**
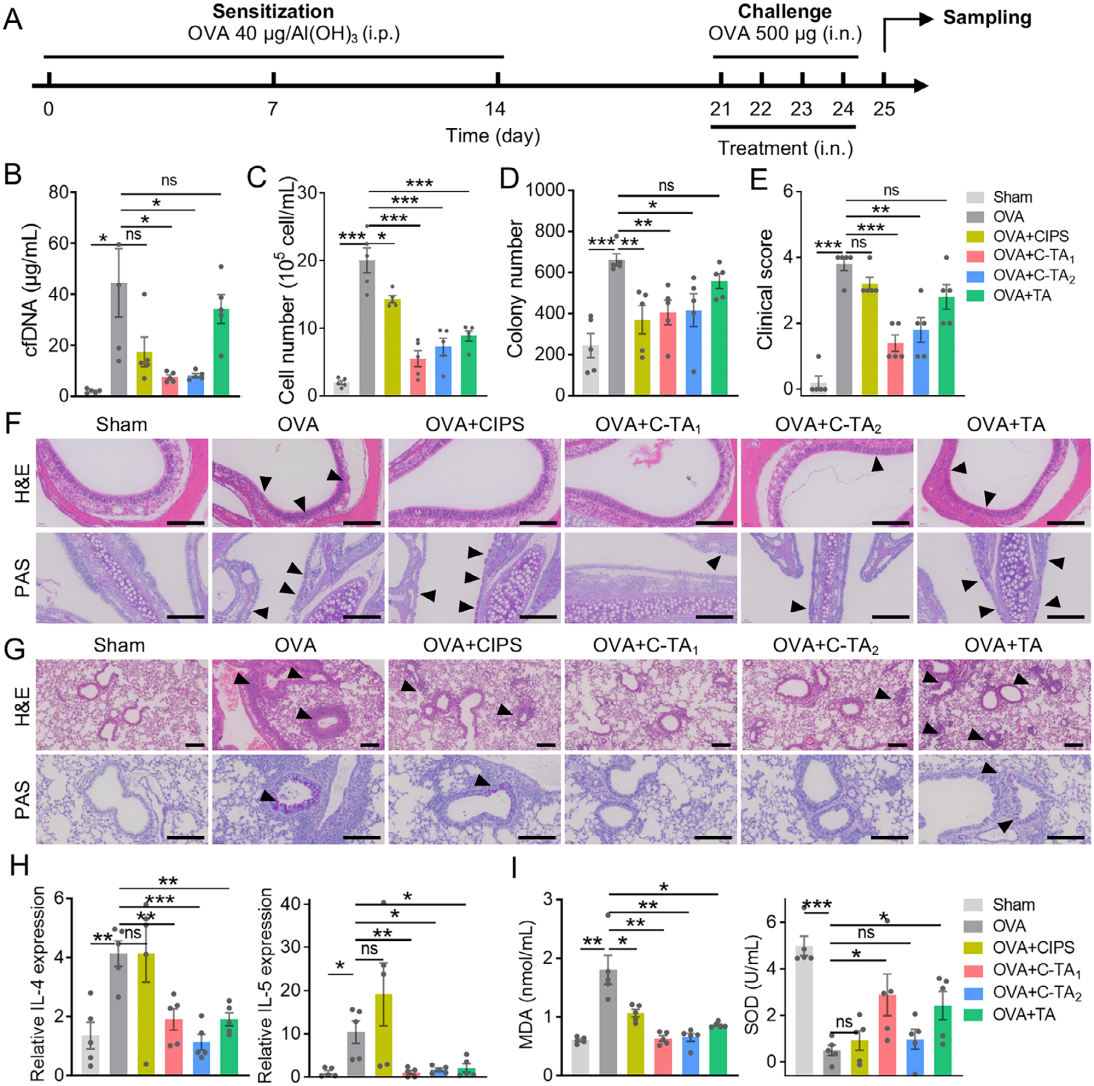
Multifunctional nanosheets alleviated OVA‐induced eosinophilic airway inflammation. (A) Schematic diagram of the OVA‐induced mouse model and treatment strategy. (B) cfDNA concentration in the BALF of experimental mice. (C) Cell counting in the BALF of mice in each group. (D) The agar plate colony counting in the BALF after incubation in the plate for 12 h. (E) Clinical scores based on the H&E staining images of lung slices. (F) H&E staining and PAS staining images of the nasal mucosa from experimental mice. (G) H&E staining and PAS staining images of lungs from experimental mice. Scale bars: 200 µm. (H) RT‐qPCR results of IL‐4 and IL‐5 expressions in the lungs of mice in each group. (I) MDA and SOD levels in BALF of mice in each group. Data are presented as mean ± SEM (*n* = 5, Student's *t*‐test, two‐tailed, ns: no significant difference, *P < 0.05, **P < 0.01, ***P < 0.001).

Compared to Th1‐type neutrophilic inflammation, Th2‐type inflammation dominated by eosinophils was more frequently diagnosed in patients with recalcitrant airway diseases. In this study, OVA‐stimulated mouse models were employed for in vivo investigations of eosinophilic airway inflammation. Firstly, the mice were sensitized by intraperitoneal injection of OVA combined with Al(OH)_3_ three times on 0, 7, and 14 days. Subsequently, the above mice were intranasally challenged with OVA four times in total on 21, 22, 23, and 24 days. In addition, the mice in the therapeutic groups were intranasally treated with nanosheets (5 mg/kg) or dexamethasone (DEX, 5 mg/kg) at 4 h after the challenge each time, and the mice were sacrificed on day 25 (Figure 6A). There was no significant change in the body weight of mice after administration, suggesting the in vivo biosafety of functional nanosheets (Figure S41). Compared to the sham mice, cfDNA levels in the BALF of OVA group increased more than twenty‐fold (from 2.10 ± 0.57 µg/mL to 44.48 ± 13.41 µg/mL), while the value decreased to nearly healthy level after C‐TA_1_ and C‐TA_2_ treatments (7.57 ± 0.86 µg/mL, and 8.19 ± 0.75 µg/mL). However, the effect is less significant in the DEX group (Figure 6B and Figure S42A), proving the excellent in vivo cfDNA scavenging ability of TA‐covered nanosheets. In addition to the cfDNA, the total cell counts in BALF declined to 0.55×10^6^ cell/mL and 0.72×10^6^ cell/mL after C‐TA_1_ and C‐TA_2_ treatment compared to the inflammatory group (2.00×10^6^ cell/mL), whereas DEX showed almost no therapeutic effect (Figure 6C and Figure S42B). On the other hand, the bacterial content in the treated groups was largely reduced to near‐healthy levels in the sham group (Figure 6D and Figure S43).

H&E staining images depicted that the infiltration of inflammatory cells was suppressed in the nasal mucosa and lungs, and the quantified clinical scores were reduced after C‐TA_1_ and C‐TA_2_ treatment (from 3.83 to 1.50 and 1.67), respectively (Figure 6E–G and Figure S42C,E,F). Moreover, PAS staining images manifested the goblet cell hyperplasia and mucus layer thickening in the airway of OVA‐stimulated mice, suggesting that nanosheets are promising to suppress excessive mucus production in patients with chronic airway disease (Figure 6F,G and Figure S42D–F). C‐TA_1_ treatment exhibited a comparable effect to the DEX group in reducing inflammatory cell infiltration and mucus production. The elevated expression of type 2 inflammatory cytokines in mouse lungs (such as IL‐4 and IL‐5) of the OVA group was also proved by RT‐qPCR. In consistency, C‐TA_1_ treatments declined mucus generation (from 70.0% to 5.2%) (Figures S44) and cytokines expressions (relative expression of IL4: from 4.13 to 1.90; IL‐5: from 10.36 to 1.05), while DEX treatment only reduced IL‐5 expression (Figure 6H and Figure S42G,H). In addition, the in vivo antioxidant effects were evaluated in the experimental mice, and C‐TA_1_ and C‐TA_2_ treatments markedly declined the content of MDA and restored the level of SOD, which is consistent with previous antioxidant tests (Figure 6I). Although DEX has been proven effective in treating inflammatory diseases, long‐term use can lead to side effects such as insomnia, adrenal suppression, metabolic problems, and increased risk of infection [57, 58]. The TA‐covered nanosheets exhibited more robust anti‐inflammatory effects and could avoid the undesired side effects of DEX, confirming the significance of the developed multi‐targeting therapeutic strategy.

**FIGURE 7:**
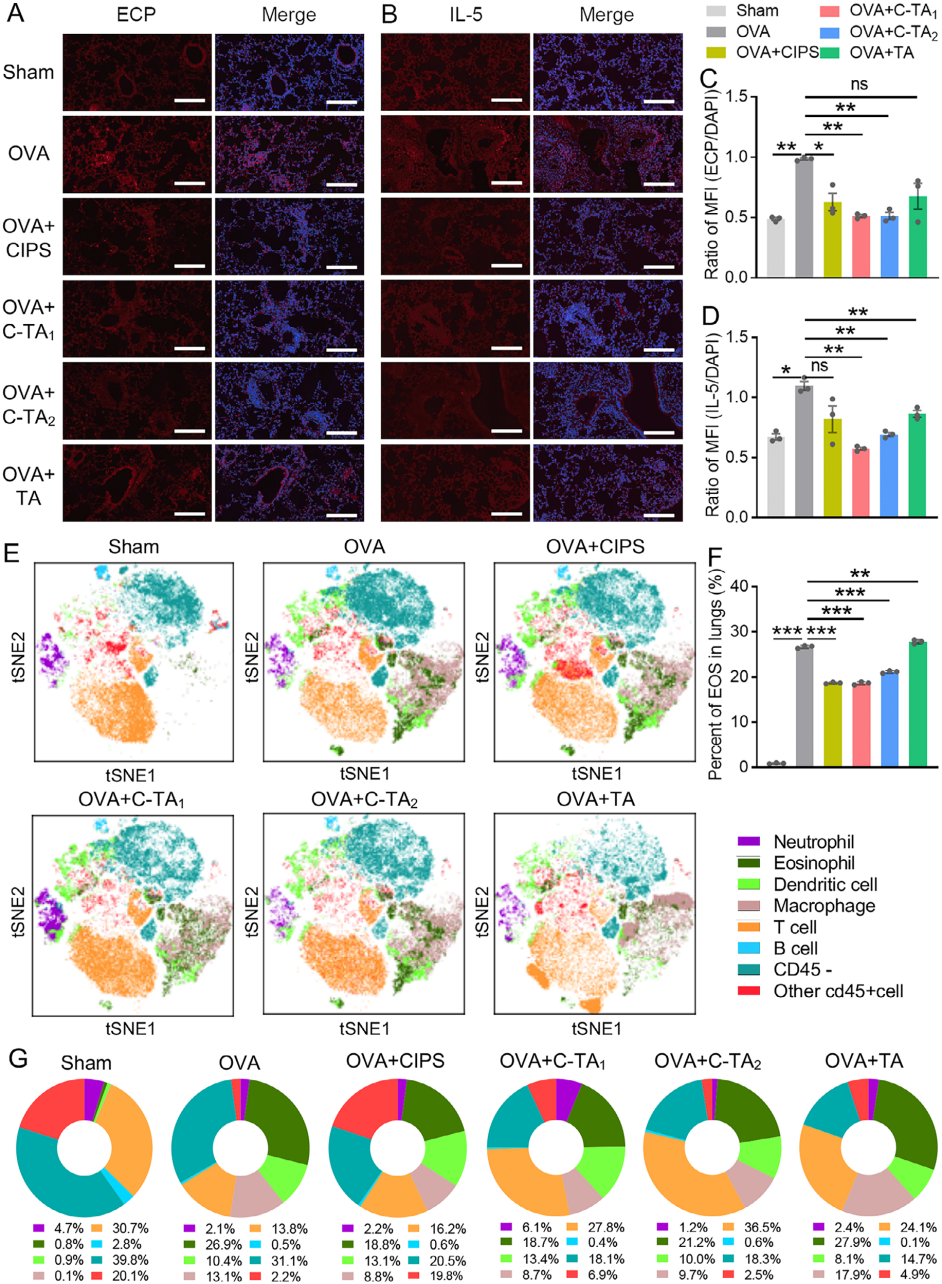
Multifunctional nanosheets alleviated the cytokine generation and immune cell infiltration in inflammatory airway tissues. (A, B) Representative DAPI/ECP co‐staining images and DAPI/IL‐5 co‐staining images of lungs from experimental mice. Scale bars: 200 µm. (C, D) Quantitative analysis of ECP/DAPI fluorescence ratio and IL‐5/DAPI fluorescence ratio in the representative images. (E) Visualization of t‐SNE analysis of various types of cells in the lungs (Neu, Eos, DC, Mac, T cells, B cells etc.). (F) The calculated percentage of eosinophils in total lung cells according to the flow cytometry results. (G) The quantitative analysis of various types of cells in lungs based on the flow cytometry results. Data are presented as mean ± SEM (*n* = 3, Student's *t*‐test, two‐tailed, ns: no significant difference, *P < 0.05, **P < 0.01, ***P < 0.001).

Based on previous publications, OVA stimulation was able to promote the infiltration of immune cells and the expression of pro‐inflammatory factors in the airway of model mice. Immunofluorescence staining of ECP and IL‐5 was exploited on mouse lung slices to determine eosinophil infiltration and IL‐5 generation levels. Compared to the sham mice, a relative increase in ECP and IL‐5 positive area was observed in the representative images of the inflammatory group. CIPS treatment mitigated eosinophil infiltration (to 63.63%) and IL‐5 generation (to 74.54%) in lung tissues, and the relief was more pronounced in the C‐TA_1_ (to 51.51% and 51.81%) and C‐TA_2_ (to 51.51% and 62.72%) treatment groups (Figure 7A–D). Similar to ECP and IL‐5, nanosheet treatments reduced the infiltration of neutrophils and NET formation in the lungs of mice, which was confirmed by immunofluorescence staining of CitH3 and Ly6G (Figure S45).

Based on the above results, we conducted a comprehensive analysis of immune cell infiltration in mouse lungs using multi‐channel flow cytometry (Figure 7E). Compared to the sham group, the infiltration of CD45^+^ immune cells in the lungs of inflammatory mice significantly elevated (Figure S46). More specifically, compared to the OVA group, the infiltration of eosinophils and macrophages in total lung cells declined from 26.67% and 12.15% to 18.61% and 7.77% in the C‐TA_1_ group and 21.12% and 9.16% in the C‐TA_2_ group, highlighting the therapeutic effects of TA‐covered nanosheets for eosinophilic airway inflammation (Figure 7F–G and Figure S46). Interestingly, the percent of specific immune cells, such as T cells, descended in the OVA‐stimulated mice, which was also reversed after treatment with C‐TA_1_ and C‐TA_2_ (Figure 7G and Figure S46C). These consequences collectively confirmed the anti‐inflammatory properties of TA‐modified nanosheets and the superiority of 2D nanostructures in the development of therapeutic nanoplatforms.

### 2.9 Functional Nanosheets Modulated the Imbalance of Airway Microbiota

**FIGURE 8:**
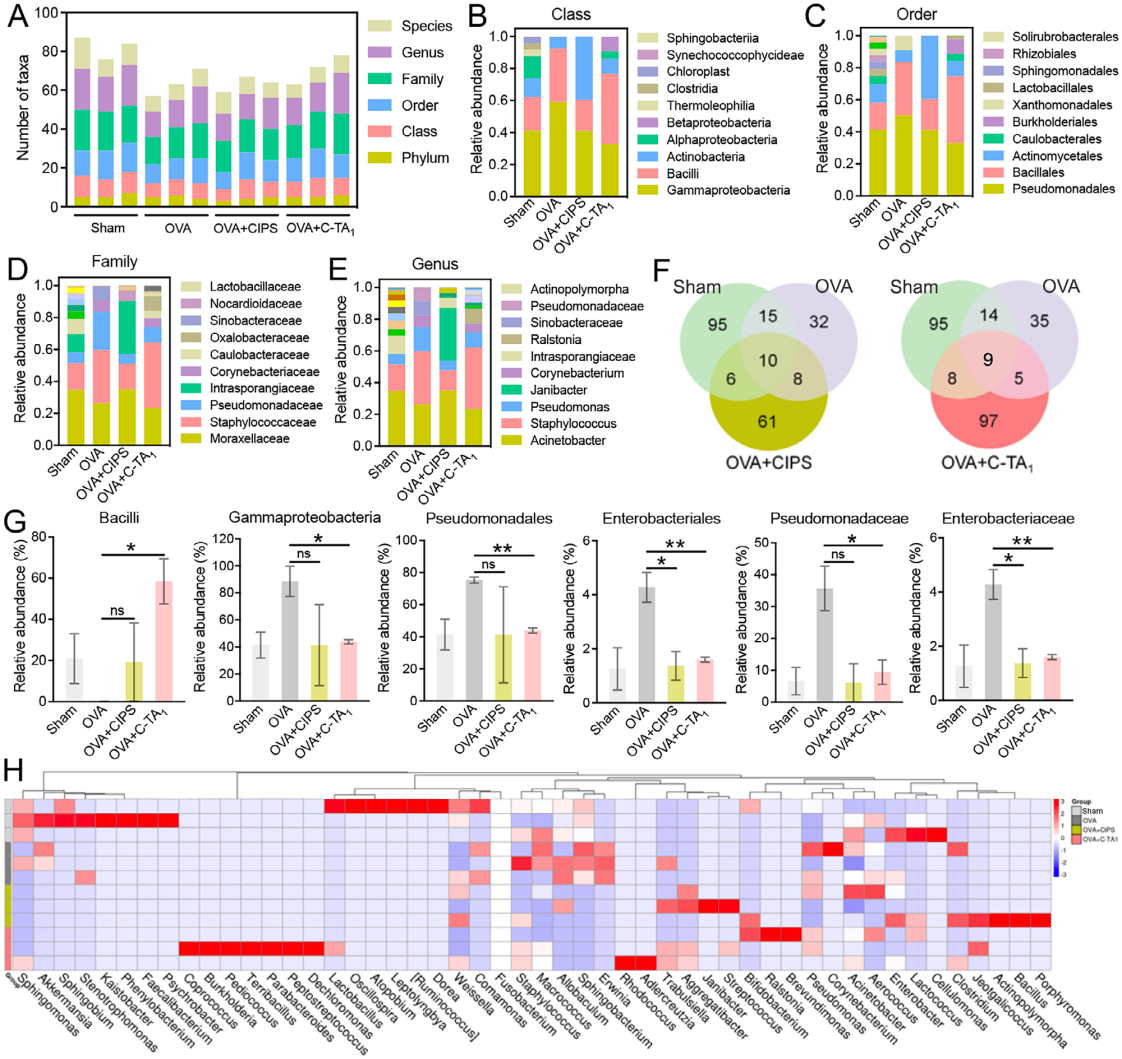
The microbiota in the airway of model mice analyzed using 16s RNA sequencing. (A) Statistical chart of microbial classification units at various levels. (B–E) Histogram of the relative abundance of each group at the (B) class level, (C) order level, (D) family level, and (E) genus level. (F) Veen of intestinal bacteria in mice with sham, OVA, OVA+CIPS and OVA+C‐TA_1_ groups. (G) Relative abundance of *Bacilli*, *Gammaproteobacteria*, *Pseudomonadales*, *Enterobacteriales*, *Pseudomonadaceae*, and *Enterobacteriaceae*. (H) Heatmap of the relative abundance of genus‐level taxa. Data are presented as mean ± SEM (*n* = 3, Student's *t*‐test, two‐tailed, ns: no significant difference, *P < 0.05, **P < 0.01).

Many recent studies revealed that the balance of airway microbiota exerted a pivotal influence on airway immune responses and vice versa [35]. As C‐TA_1_ treatment considerably alleviated airway inflammation, it is markedly meaningful to explore whether anti‐inflammatory therapy is beneficial to restore the balance of airway microbiota. Therefore, 16S rRNA gene sequencing was adopted to analyze the changes of microbiota in BALF of experimental mice. Firstly, we analyzed the abundance of microbiota in the BALF at various levels and found that the cumulative degree of each level in the OVA‐treated mice was significantly reduced compared to the sham group (Figure 8A). More detailed analysis indicated the decline of abundance at the phylum level, class level, order level, family level, and genus level (Figure 8B–E and Figure S47), and C‐TA_1_ treatment improved these abnormal changes. Specifically, compared with the sham group, the abundance of *Firmicutes*, *Bacilli*, descended and the abundance of *Bacteroidetes*, *Gammaproteobacteria*, *Pseudomonadales*, *Enterobacteriales*, *Pseudomonadaceae*, and *Enterobacteriaceae* is increased in the OVA‐treated mice, respectively (Figure 8G and Figure S48). Fascinatingly, C‐TA_1_ treatment modulated the microbiota balance of these bacteria, which is equivalent to the sham mice. In chronic pulmonary inflammation, the enrichment of *Lactobacillus* and *Pseudomonadales* from the pathological bronchoalveolar system of patients is associated with an enhanced inflammatory response [59, 60]. The decrease of the communities of *Firmicutes* and *Bacilli* was probably to induce dysregulation of the pulmonary microbial, which is also related to the progression of inflammation.

Species diversity is a valuable indicator of community composition and characteristics, including alpha diversity and beta diversity [61]. Alpha diversity refers to the diversity within a specific habitat or community, measuring the number of species present and their relative abundance [62, 63]. We analyzed alpha diversity using the Chao1 index, Shannon index, and Simpson diversity index. Among them, the Chao1 index mainly reflects the difference between the practical richness of species in a sample and the estimated richness of the community. The Shannon index describes the disorder and uncertainty of individual occurrences between species, while the Simpson diversity index reflects the probability of two consecutive samples belonging to different species. The results showed that the Chao1 index, the Shannon index, and the Simpson diversity index decreased in the OVA‐treated mice, indicating a decline of alpha diversity in the inflammatory airway. However, C‐TA_1_ treatment promoted alpha diversity in pathological conditions, and the indexes in treated mice were approximately equal to the sham group (Figure S49). At the same time, we also conducted community analysis, and the results manifested that when the sequences clustered at a 100% similarity level, although the number of operational taxonomic units (OTU) shared by the sham group and C‐TA_1_ group did not increase compared to the OVA group, the number of different OTU promoted, which may also be the key to improving inflammation (Figure 8F).

To further compare the differences in species composition between groups and unravel the distribution of species abundance in each sample, a heatmap based on the detailed gene sequencing data was depicted. The species clustering heatmap at the genus level showed that compared to the sham group, the increased microbial community in OVA‐treated mice included *Corynebacterium*, *Pseudomonsa*, *Macrococcus*, *Sphingobacterium*, *Erwinia*, *Allobaculum*, *Staphylococcus*, and C‐TA_1_ treatment restored the microbial community (Figure 8H). The changes of these bacteria altered the airway microbiota and regulated the infiltration of immune cells in the airway tissues. In addition, the results of the double cluster analysis disclosed that the microbiota after C‐TA_1_ treatment was more similar to that of the sham group (Figure S50). In summary, the anti‐inflammatory treatment by C‐TA_1_ was helpful in restoring the disordered airway microbiota, and the modulation of microbiota disorder was also beneficial for the further recovery of dysregulated airway inflammation.

## 3 Conclusion

In recent years, the incidence of recalcitrant airway inflammatory disorders has increased rapidly, while the commonly used glucocorticoids remain unsatisfactory in clinical treatment, and the exploration of more effective therapeutic strategies remains challenging. The analysis of nasal secretions and nasal polys from CRSwNP patients substantiated that cfDNA is associated with both neutrophilic and eosinophilic immune cascades of dysregulated inflammation in the airway. In addition to cfDNA, excessive RONS generation, along with bacterial infection, are closely relevant to airway inflammatory diseases. Therefore, the development of multi‐targeting therapeutic strategies is urgently needed in clinical practice. In this work, we designed and synthesized multifunctional TA‐covered CIPS nanosheets (C‐TA_1_; w/w = 1:1) for the treatment of recalcitrant airway inflammation. Our results depicted that C‐TA_1_ nanosheets exhibited excellent anti‐inflammatory properties, including (1) cfDNA elimination to alleviate neutrophilic/eosinophilic inflammation mediated by the TLR9 pathway, (2) prevention of RONS‐induced tissue injury and inflammatory response, (3) modulation of the airway microbiota through multi‐targeting therapeutic strategy, and (4) outstanding biocompatibility both in vitro and in vivo. Moreover, the analysis of multi‐channel flow cytometry unveiled that the infiltration of immune cells, including neutrophils, eosinophils, and macrophages, was markedly descended in the lungs of nanosheet‐treated mice. In addition, our approach entailed the therapeutic effects of C‐TA_1_ outperformed CIPS, TA, and C‐TA_2_, suggesting the importance of 2D nanostructure and TA modification with appropriate ratio in the construction of multi‐targeting therapeutic nanoplatforms. These results proposed a potential tackling strategy for severe airway inflammation, which also provided new inspirations for the treatment of other intractable inflammatory disorders.

## 4 Materials and Methods

### 4.1 Materials

Ultra‐high purity CuInP_2_S_6_ (CIPS) powder was purchased from Beike 2D Materials Co., Ltd. (Suzhou, China). *N*‐butyllithium in *n*‐hexane was purchased from Shanghai Macklin Biochemical Technology Co., Ltd. (Shanghai, China). *N*‐hexane was purchased from Beijing InnoChem Science & Technology Co., Ltd. (Beijing, China). Tannic acid, sodium hydroxide, Quant‐iT PicoGreen Kit, fluorescein isothiocyanate (FITC), cell counting kit‐8 (CCK‐8) assay, 4,6‐diamidino‐2‐phenylindole (DAPI), were purchased from Fisher Scientific and used without additional purification. Ampicillin Sodium (AMP) Solution and Kanamycin Sulfate Solution (Kana) were purchased from Sangon Biotech (Shanghai) Co., Ltd. (Shanghai, China). Lipopolysaccharide (LPS), 2′, 7′‐dichlorofluorescin diacetate (DCFH‐DA), and ovalbumin (OVA) were purchased from Sigma‐Aldrich (St. Louis, USA). Milli‐Q water was used in all experiments.

### 4.2 Methods

Atomic force microscope (AFM) was performed with Dimension Fastscan Atomic Force Microscope (Bruker, Germany). transmission electron microscope (TEM) was performed with Thermo Tecnai G2 Spirit T120 kV. Zeta potential and dynamic light scattering (DLS) data were recorded on Brookhaven Zeta potential and particle size analyzer. Ultraviolet–visible spectrophotometry (UV–vis) absorption spectra, quanti‐Blue, and CCK‐8 assays were recorded on a microplate reader (Thermo Fisher Scientific, US). Fourier transform infrared spectroscopy (FTIR) was measured on Vertex70‐Hyperion3000. Confocal laser scanning microscopy (CLSM) experiments were conducted with Leica TCS SP8 and quantification of the specified color area was calculated by Image J software. Biodistribution fluorescent images were recorded with an IVIS spectrum system (PerkinElmer, USA). H&E, PAS staining slices were scanned using a high throughput slide scanning image analysis system SQSL‐510 (Shengqiang, China). Real‐time quantitative polymerase chain reaction (RT‐qPCR) was measured with ABI QuantStudio 7 Flex (Thermofisher, US). Flow cytometer was performed using a BECKMAN Cyto FLEX S flow cytometer (BECKMAN, Germany).

### 4.3 Clinical Sample Collection and Analysis

10 CRSwNP patients and 10 healthy volunteers (deviation of nasal septum) in the Sixth Affiliated Hospital of Sun Yat‐sen University were included in this study. Nasal secretions were collected with an expansive sponge, and nasal polys of CRSwNP patients and nasal mucosa of healthy volunteers were collected during the surgery, which was approved by the ethics committee of Sun Yat‐sen University ([2023] KSY No.63; 2023ZSLYEC‐497).

The nasal polyps and nasal mucosa were fixed with 4% paraformaldehyde for 24 h. After that, the tissues were embedded in paraffin, sliced, and subjected to CitH3, Ly6G, ECP, and MPO immunostaining. The images of slices were recorded by CLSM, and the positive areas of markers were analyzed by ImageJ software.

### 4.4 Synthesis of CIPS Nanosheets

The CIPS nanosheets were prepared using lithium anions as intercalation agents as described previously [64]. In brief, CIPS powder (100 mg) was added to a Schlenk flask, followed by the addition of 20 mL of *n*‐butyllithium in n‐hexane. The mixture was stirred at room temperature under nitrogen for 48 h. 50 mL of *n*‐hexane was added to the reaction solution and centrifuged at 5000 rpm for 10 min. The supernatant was discarded, and the precipitate was dispersed with *n*‐hexane and washed with *n*‐hexane (5000 rpm). Finally, the precipitate was dispersed in Milli‐Q water and centrifuged at 10,000 rpm for 30 min. The supernatant (CIPS 1WS) was dialyzed in pure water (MWCO = 3500 K) for 48 h. Precipitation resuspended and centrifuged at 5000 rpm for 30 min obtained supernatant (CIPS 5KS) and precipitate (CIPS 5KP).

### 4.5 Synthesis of C‐TA

10 mg CIPS 1WS nanosheets were suspended in 10 mL Milli‐Q water. Then, the pH was adjusted to 8.0 using a 10 m NaOH solution. After that, 1, 5, 10, 20, 30, 50, 100, or 250 mg TA was added to the above solution, and the mixture was stirred at room temperature for 8 h. The mixture was dialyzed in pure water (MWCO = 10,000 K) for 48 h before the solutions were collected and lyophilized. TA‐covered CIPS nanosheets with different reaction ratios were termed as C‐TA_0.1_, C‐TA_0.5_, C‐TA_1_, C‐TA_2_, C‐TA_3_, C‐TA_5_, C‐TA_10_, and C‐TA_25_.

### 4.6 cfDNA and NETs Binding Assay

cfDNA was measured using Quant‐iT Pico‐Green Kit. Pico‐green reagent emits fluorescence (Ex/Em = 480/520 nm) only after binding to dsDNA and does not emit fluorescence without dsDNA. When pico‐green binds to dsDNA, the intermolecular forces of pico‐green reagent change, resulting in a significant enhancement of the fluorescence signal. The fluorescence intensity is proportional to the concentration of dsDNA in the detection range, the dsDNA concentration can be calculated based on the fluorescence intensity. For the cfDNA and NETs binding test, the nanosheets with a series of concentrations were mixed with cfDNA (500 ng/mL) or NETs (1000 ng/mL) solution in equal volumes at room temperature for 30 min, and then the pico‐green reagent was added. DsDNA concentration was determined by the fluorescence intensity in the solution, and then the cfDNA binding efficiency was calculated by the variation of free ds DNA amount in the solution.

### 4.7 Cell Culture

Neutrophils were extracted from the peripheral blood of volunteers using anticoagulated blood with standardized methods [65], then cultured in RPMI1640 medium with 1% penicillin–streptomycin (PS) and 10% fetal bovine serum (FBS) at 37°C with 5% CO_2_. BEAS‐2B cells were cultured in a high‐glucose Dulbecco's modified eagle medium (DMEM) with 1% PS and 10% FBS at 37°C with 5% CO_2_. RPMI 2650 cells were cultured in Minimum Essential Medium (MEM) with 1% PS and 10% FBS at 37°C with 5% CO_2_.

### 4.8 NET Formation Assay

2×10^5^ neutrophils were seeded into a 24‐well plate with covered glass on the bottom. To evaluate the inhibitory effect of nanosheets on NET formation. Neutrophils were sensitized with CpG ODN1826 and co‐cultured with 20 µg/mL of the CIPS, C‐TA_1_, or C‐TA_2_ for 24 h. Immunofluorescence staining was performed using DAPI and CitH3 and recorded by a confocal laser scanning microscope (CLSM).

### 4.9 TLR3 and TLR9 Activation Tests

HEK‐Blue hTLR9 (HEK‐TLR9) cells were cultured following the manufacturer's protocol. Briefly, 8×10^4^ HEK‐TLR9 cells were seeded into a 96‐well plate. When the cell density reached 50%–80%, CpG ODN 2006 was added and co‐incubated with different concentrations of the CIPS, C‐TA_1_ or C‐TA_2_ for 24 h. The medium was mixed with QUANTI‐Blue Solution, and the OD value at 450 nm was qualified using a Multiplate Reader. The OD value (450 nm) was used to calculate the level of the secreted alkaline phosphatase (SEAP) in the medium to determine the TLR9 activation. Similar protocols were performed to detect TLR3 activation.

### 4.10 Antioxidant Studies

ABTS^+^ and DPPH· assays were adopted to determine the RONS reduction capacity. To detect the ABTS^+^ scavenging capacity, 5 mL of 7.4 mmol/mL ABTS, and 5 mL of 2.6 mmol/mL K_2_S_2_O_8_ were mixed and reacted at room temperature (RT) for 10 h in the dark. Then, the above reaction product was diluted with absolute ethanol to achieve an absorbance (734 nm) of approximately 0.7 to obtain the ABTS^+^ working liquid. Subsequently, 160 µL of ABTS^+^ working liquid was mixed with 40 µL of nanosheets with different concentrations. The mixture was reacted for 6 min at RT, and the working liquid added only with ddH_2_O was considered as the control group. The absorption value after the reaction was measured at 734 nm. The ABTS^+^ elimination (%) = (*A*_control_ − *A*_sample_)/*A*_control_ × 100%.

To detect the DPPH· scavenging capacity, 2 mmol/mL DPPH· solution was prepared with absolute ethanol. Subsequently, 160 µL of the DPPH· solution was mixed with 40 µL of nanosheets with different concentrations in a 96‐well plant. The mixture was reacted for 30 min at RT, and the working liquid added only with ddH_2_O was considered as the control group. The absorption curve value was then measured from 400 nm to 600 nm and the OD value was qualified at 517 nm. The DPPH∙ elimination (%) = (*A*_control_ − *A*_sample_)/*A*_control_ × 100%.

To detect the ∙O_2_^−^ scavenging capacity, 40 µL of 0.05 unit/mL xanthine oxidase and 40 µL of 1.2 mmol/mL xanthine solution were mixed in PBS at 37°C for 40 min to generate ∙O_2_^−^. And 20 µL of nanosheets with different concentrations were added to the above solutions and incubated for another 40 min. Then, 100 µL of 1 mg/mL dihydroethidium (DHE) solution was added, and the mixture added with 20 µL of ddH_2_O was considered as the control group. The concentration of ∙O_2_^−^ was determined by measuring the fluorescence intensity of ethidium (Ex/Em = 470/610 nm). The ∙O_2_^−^ elimination (%) = (*A*_control_ − *A*_sample_)/*A*_control_ × 100%.

### 4.11 Intracellular RONS Detection

BEAS‐2B cells (10^4^ cells/well) or RPMI2650 cells (2 × 10^4^ cells/well) were seeded into 96‐well plates (black) overnight. Subsequently, 10 µg/mL LPS was added to stimulate the cells for 4 h before the nanosheets were introduced and cultured for the following 4 h. After that, the culture medium was removed and the cells were washed twice with PBS. Finally, DCFH‐DA (10 µm) was added into the cell medium and incubated for 30 min. The fluorescent images were taken by fluorescence microscopy.

### 4.12 Antibacterial Studies

*E. coli*, AmpR *E. coli*, KanR *E. coli*, and *S. aureus* were cultured in an optimum culture medium. Then, the individual colony of the bacteria was added to 5 mL of fresh LB medium and incubated on a shaker (220 rpm) at 37°C for 4 h until they reached the logarithmic growth phase. Then the bacterial solution was co‐cultured with nanosheets with different concentrations for 8–12 h. The bacterial concentration in the solution was quantified by measuring the absorbance at 600 nm using Nanodrop. Additionally, the diluted bacterial solution and nanosheets with different concentrations were spread on optimum culture medium agar. The bacterial images were recorded by a multifunctional imaging device after 24 h incubation.

The *E. coli* or *S. aureus* was incubated with 100 µg/mL nanosheets for 8 h and then LIVE/DEAD BacLight Bacterial Viability Kits were applied for bacterial live/dead staining. In brief, the bacteria were centrifuged at 10,000 g for 10 min and washed with PBS multiple times after incubation with nanosheets. Subsequently, fluorescence staining was performed using LIVE/DEAD Kits (SYTO 9 and propidium) for 15 min. Finally, 5 µL of bacterial suspension was dropped onto a glass slide and the fluorescent images were recorded by CLSM. (live: Ex/Em = 480/500 nm; dead: Ex/Em = 490/635 nm).

### 4.13 Antiviral Studies

Pseudovirus production and neutralization assay were performed according to a previous study [66]. In brief, pcDNA3.1‐2019‐nCoV‐Spike, pSPAX2 and pLenti‐CMV‐puro‐Luc (168w‐1) were co‐transfected to HEK293T using Lipo8000 to generation WT SARS‐CoV‐2‐Spike (Wuhan‐Hu‐1) pseudovirus. The virus‐containing supernatant was harvested after 72 h incubation and stored at −80°C. The BA.5 Omicron‐variant spike pseudovirus was obtained using the same method. 2×10^4^ hACE2‐293T cells were seeded on the 96‐well plate (black) and incubated at 37°C overnight. Nanosheets with different concentrations were added to pseudovirus solution at 37°C for 1 h in DMEM. The co‐incubated solution was subjected with 10 µg/mL polybrene to the hACE2‐293T cells for 6 h. The hACE2‐293T cells incubated only with medium and only with pseudovirus were considered as negative control and positive group, respectively. Then, the culture medium was replaced and the cells were incubated for another 42 h at 37°C with 5% CO_2_. Finally, infected cells were lysed and measured by the luciferase assay according to the manufacturer's instructions. The relative light unit (RLU) was measured by a microplate reader. The virus inhibition rate (%) = (RLU_PC_ − RLU_sample_)/(RLU_control_ − RLU_NC_) × 100%.

### 4.14 Establishment of Animal Models

Animal experiments were approved by the ethics committee of the Sixth Affiliated Hospital of Sun Yat‐sen University (No. IACUC‐2023021601). BALB/c female mice (6–8 weeks) were purchased from GemPharmatech (Guangdong, China), and housed in specific pathogen‐free (SPF) conditions at the Experimental Animal Center, The Sixth Affiliated Hospital, Sun Yat‐sen University.

An LPS‐stimulated animal model with neutrophilic airway inflammation was established using modified protocols as previously described [56]. The mice were randomly divided into six groups (Sham, LPS, LPS+CIPS, LPS+C‐TA_1_, LPS+C‐TA_2_, and LPS+TA) with 5 mice per group. 2 µg of LPS in 50 µL PBS was instilled intranasally for the experimental mice. After 4 h, 50 µg of CIPS, C‐TA_1_, C‐TA_2_, or TA was intranasally treated for the LPS pre‐treatment mice. Sham mice were intranasally instilled with 50 µL of PBS instead of LPS stimulation. All of the mice were euthanized at 24 h after treatment. And maxillary bones (MBs), lungs, serum, and bronchoalveolar lavage fluid (BALF) were collected for further evaluation.

An OVA‐induced animal model with eosinophilic airway inflammation was established using modified protocols as previously described [23]. The mice were randomly divided into six groups (Sham, OVA, OVA+CIPS, OVA+C‐TA_1_, OVA+C‐TA_2_, and OVA+TA) with 5 mice per group. These experimental mice were intraperitoneally injected with 40 µg OVA and 20 mg of Al(OH)_3_ in 100 µL PBS three times at 0, 7, and 14 days. From 21 to 24 days, the experimental mice were intranasally challenged with 50 µg OVA once per day. After 4 h, 50 µg of CIPS, C‐TA_1_, C‐TA_2_, or TA was intranasally treated for the OVA pre‐treated mice. Sham mice were sensitized and challenged with PBS instead of OVA stimulation. Mice were euthanized 24 h after the final treatment. And MBs, lungs, serum, and BALF were collected for further evaluation.

### 4.15 Cell and Bacterial Counting in BALF

According to the published protocol [67], BALF was obtained by infusing 0.6 mL of saline three times into the whole lungs via a tracheal cannula. Subsequently, BALF was centrifuged at 3000 g for 10 min, and the supernatant was stored at −80°C. On the other hand, the cell pellet was resuspended in 1 mL of PBS for cell counting using a flow cytometer. The resuspended solution was diluted in Luria–Bertani Medium and spread on agar plates. The photos were taken after 24 h incubation at 37°C.

### 4.16 Histopathological and Immunofluorescence Staining

The MBs, hearts, livers, spleens, lungs, and kidneys were collected and fixed with 4% paraformaldehyde for 24 h. For the nasal mucosa analysis, the MBs were first decalcified in EDTA solution for 3 weeks. Subsequently, the decalcified MBs and lungs were embedded in paraffin, sliced, and subjected to hematoxylin and eosin (H&E) or periodic acid‐Schiff (PAS) staining. The inflammatory degree based on H&E staining was scored as follows [68]: 0 for no inflammatory cell infiltration; 1 for a small amount of inflammatory cell infiltration; 2 for one layer of inflammatory cell infiltration; 3 for 2‐ 4 layers of inflammatory cell infiltration; and 4 for more than 4 layers of inflammatory cell infiltration. In addition, immunofluorescence staining was performed on these slices using ECP, IL‐5, CitH3, and Ly6G antibodies.

### 4.17 RT‐qPCR

Total RNA was extracted from isolated lungs by SteadyPure Quick RNA Extraction Kit and reverse transcribed into cDNA with the PrimeScript RT reagent kit. ABI QuantStudio 7 Flex and SYBR PreMix Ex Taq were used for RT‐qPCR to quantify the targeted mRNA level. The data were analyzed by the 2^−ΔΔ^ *^Ct^* method and reported as the relative expression level. The primers are listed in Table S1.

### 4.18 Quantification of Redox Index In Vivo

The levels of MDA and SOD in mouse BALF were measured following the protocols provided by the respective assay kits.

### 4.19 Hepatic and Renal Function Detection

The peripheral blood of experimental mice was collected from the eye socket before the mice were sacrificed, and serum was prepared by centrifugation at 5000 rpm for 10 min. Aspartate aminotransferase (AST), alanine aminotransferase (ALT), creatinine (CREA), urea (URE), and creatine kinase (CK) levels in serum were measured using an automatic biochemical analyzer.

### 4.20 Biodistribution Assay In Vivo

Mice were intranasally administered with PBS, Cy5‐labeled CIPS (CIPS‐CY5, 50 µg), C‐TA_1_ (C‐TA_1_‐CY5, 50 µg), and C‐TA_2_ (C‐TA_2_‐CY5, 50 µg) while the mice treated with PBS were considered as sham group. Fluorescence images of the noses and major organs (hearts, lungs, livers, spleens, and kidneys) were recorded by using the IVIS Spectrum system at 4 h, 1 day, 3 days, and 7 days post‐instillation (Ex/Em = 640/720 nm). CY5 fluorescence (Ex/Em = 640/720 nm) was measured in serum samples by a microplate reader to evaluate the concentration of nanosheets in blood.

### 4.21 Flow Cytometry Analysis

The lungs were collected and then cut into pieces at the end of the animal experiments. Subsequently, the lung tissues were gently ground to obtain single‐cell suspension. Then, the cells were filtered through a 70 µm cell sieve and treated with red blood cell lysis buffer before being washed with PBS twice. Subsequently, the cells were labeled with antibodies and the cell type was analyzed by flow cytometry. The antibodies used in flow cytometry are detailed in Table S2.

### 4.22 16s RNA Sequencing Analysis

The genomic DNA of BALF from experimental mice was extracted using the OMEGA Soil DNA Kit (M5635‐02) (Omega Bio‐Tek, Norcross, GA, USA). The bacterial 16S rRNA gene V3‐V4 region was amplified using the forward primer 338F (5′‐ACTCCTACGGGAGGCAGCA‐3′) and the reverse primer 806R (5′‐GGACTACHVGGGTWTCTAAT‐3′). Sample‐specific 7‐bp barcodes were incorporated into the primers for multiplex sequencing. Polymerase chain reaction (PCR) amplicons were purified, quantified, and pooled in equal amounts. The paired‐end 2 × 250 bp sequencing was performed using the Illumina NovaSeq platform using the NovaSeq 6000 SP Reagent Kit (500 cycles) at Suzhou PANOMIX Biomedical Tech Co., LTD.

### 4.23 Statistical Analysis

Graph Pad Prism 8.2 was employed for data analysis and calculation of statistical significance. The data are presented as mean ± SEM. Statistical differences between experimental groups were assessed using unpaired two‐tailed Student's *t*‐test or Wilcoxon rank sum test. Spearman correlation analysis was used to analyze the correlation between the two variables.

## Author Contributions

Conceptualization: M.L. and Z.T. Methodology: M.L., C.X., R.M., and Z.T. Investigation: M.L., C.X., R.M., and Z.T. Visualization: M.L., C.X., X.X., Y.X., M.D., Z.X., S.Z., and J.L. Supervision: M.L., W.W., and Z.T. Writing – original draft: M.L., C.X., and Z.T. Writing – review & editing: M.L., C.X., W.W., and Z.T.

## Conflicts of Interest

The authors declare no conflicts of interest.

## Supporting information




**Supporting File 1**: exp270153‐sup‐0001‐SuppMat.docx.

## Data Availability

The data that support the findings of this study are available from the corresponding author upon reasonable request.
