# Supplementary material for: Surmounting Recalcitrant Airway Inflammatory Disorders With a Multi‐Targeting Therapeutic Strategy by Tannic Acid‐Modified CuInP2S6 Nanosheets
Source: Exploration (Beijing). 2026 Mar 12;6(2):20250073. doi: 10.1002/EXP.20250073 (PMC13094517; doi:10.1002/EXP.20250073)
Supplement: Supplementary file 1 — Supporting File 1: exp270153‐sup‐0001‐SuppMat.docx. [file EXP2-6-20250073-s001.docx]

**Supplementary Materials for**

**Surmounting recalcitrant** **airway inflammatory disorders with a multi-targeting therapeutic strategy by tannic acid-modified CuInP_2_S_6_ nanosheets**

**This PDF file includes:**

**Materials and Methods**

**Supplementary Table S1 to Table S2**

**Supplementary Figure S1-Figure S50**

**Materials and Methods**

*DPPH· assay*

To determine the DPPH· scavenging capacity, 2 mmol/mL DPPH· solution was prepared with ethanol. Subsequently, 160 μL of the DPPH· solution was incubated with 40 μL of nanosheets with different concentrations in a 96-well plate at room temperature (RT) for 30 min. The DPPH· solution incubated with only ddH_2_O was considered as the control group. The absorption value was measured at 517 nm. The DPPH· clearance (%) = (A_control_ – A_sample_)/A_control_ × 100%.

*OH· assay*

To detect the OH· reduction capacity, 30 μL of 9 mmol/mL salicylic acid solution, 30 μL of 9 mmol/mL FeSO_4_ solution, and 30 μL of nanosheets with different concentrations were added to a 96-well plate. Then, 30 μL of 8.8 mmol/mL H_2_O_2_ solution was added to the plate, and the volume was filled to 150 μL with ddH_2_O. During the same time, a control group without nanosheets and a blank group without H_2_O_2_ were established. The reactions were incubated at 37 °C for 15 min. The absorption value was measured at 510 nm. The OH· clearance (%) = (A_control_ – (A_sample_ – A_blank_))/A_control_ × 100%.

*Fluorescence value showed ROS*

BEAS-2B cells or RPMI2650 cells were seeded into a 96-well plate (black) and cultured overnight at 37 °C. Subsequently, 10 μg/mL LPS was added to stimulate the cells for 4 h before the medium was replaced by nanosheets with different concentrations and cultured for another 4 h. Next, the culture medium was removed and washed twice with PBS. Finally, the DCFH-DA was added to the cells and incubated at 37 °C for 30 min. The fluorescent value was measured by a plate reader (Ex/Em = 495/525 nm).

*Cytotoxicity test*

The cytotoxicity of CIPS, C-TA_1_, C-TA_2_, and TA were evaluated using the CCK-8 assay. 1×10^4^ cells were seeded in a 96-well plate and cultured at 37 °C with 5% CO_2_. When the cell density reached 70-80%, the medium was replaced by a medium with different concentrations of the above-mentioned nanomaterials. After 24 h or 48 h incubation, the medium was replaced again with a solution containing 10% CCK-8 reagent and incubated for 2-3 h in a 37 °C incubator. Then, the absorbance at 450 nm of the solution was examined using a Multiplate Reader to calculate cell viability.

*Hemolysis test*

1 mL of anticoagulated blood was centrifuged at 1500 rpm for 10 min, and then the supernatant was discarded. The red blood cell pellets were washed with saline, and the above process was repeated twice. After washing, saline with different concentrations of nanosheets was added, and red blood cell lysate was used as a positive control group. After incubation on a shaker at 37 °C for 1 h, the solution was centrifuged at 1500 rpm for 3 min. Finally, photos were recorded for the red blood cell solution, and the absorbance at 540 nm was measured to calculate the hemolysis rate. Hemolysis rate (%) = (Asample – Anc)/(Apc – Anc) × 100%.

*Agarose gel electrophoresis*

50 μL of cfDNA was incubated with 50 μL of 500 μg/mL CIPS, C-TA_1_, and C-TA_2_ at room temperature for 1 hour. Half of the mixture was centrifuged at 10,000 g for 10 minutes, and the resulting supernatant was collected. Both the original mixture and the supernatants were subjected to 1% agarose gel electrophoresis at 120 V for 30 minutes. An equal amount of cfDNA was used as a positive control group, and the results were detected using a chemiluminescence analyzer.

*Western Blot*

0.01 g of nasal polyps (n=6) or nasal mucosa (n=3) were added to 100 μL Radio Immunoprecipitation Assay (RIPA) lysis buffer and ground by the cryogenic grinder for protein extraction. 1×10^6^ neutrophils were seeded into a 6-well plate to evaluate the inhibitory effect of nanosheets on NET formation. Neutrophils were sensitized with CpG ODN1826 and co-cultured with 20 μg/mL of the CIPS, C-TA_1_, or C-TA_2_ for 24 h. RIPA lysis buffer was used to lyse cells for protein extraction, and the protein concentration was determined using a BCA protein quantification kit. Subsequently, 30 μg of protein was subjected to 12.5% polyacrylamide gel electrophoresis, with 80 V applied for the stacking gel and 100 V for the separation gel. Proteins were transferred to a PVDF membrane at 250 mA for 60 min. The membrane was blocked with 5% BSA in TBST at room temperature for 1 h, followed by incubation with primary antibodies: Anti-Histone H3 (mono methyl K36) Antibody (BM4316) and GAPDH Rabbit mAb (380626) at 4 °C overnight. After washing, secondary antibodies were incubated at room temperature for 1 h. Finally, an HRP chemiluminescence assay kit was added, and results were detected using a chemiluminescence analyzer.

*SEM of E. coli*

The *E. coli* solution was co-cultured with 100 μg/mL of nanosheets for 8-12 h. The solutions were then centrifuged at 10,000 g for 10 min, washed with PBS, and the supernatants were discarded. The pellets were resuspended in an electric fixative, followed by washing with PBS and dehydration with ethanol. Bacterial morphology was recorded using a HITACHI SU8100.

**Supplementary Table S1 to Table S2**

**Table S1. Primers of mouse genes used in RT-qPCR**

| Gene | Type | Sequence |
| --- | --- | --- |
| GAPDH | Forward | GTCTCCTCTGACTTCAACAGCG |
|  | Reverse | ACCACCCTGTTGCTGTAGCCAA |
| IL-8 | Forward | CCTAGGCATCTTCGTCCGTC |
|  | Reverse | TTCACCCATGGAGCATCAGG |
| IL-17A | Forward | TATCCCTCTGTGATCTGGGAAG |
|  | Reverse | ATCTTCTCGACCCTGAAAGTGA |
| IL-4 | Forward | GGTCTCAACCCCCAGCTAGT |
|  | Reverse | GCCGATGATCTCTCTCAAGTGAT |
| IL-5 | Forward | TCAGGGGCTAGACATACTGAAG |
|  | Reverse | CCAAGGAACTCTTGCAGGTAAT |

**Table S2. Fluorescent antibodies of mice used in flow cytometry**

| Antibody | Catalogue No. |
| --- | --- |
| Zombie Aqua™ Fixable Viability Kit | 423101 |
| FITC anti-mouse CD45 | 103108 |
| Brilliant Violet 605™ anti-mouse/human CD11b | 101257 |
| PerCP/Cyanine5.5 anti-mouse CD3 | 100218 |
| Brilliant Violet 650™ anti-mouse CD19 | 115541 |
| Brilliant Violet 421™ anti-mouse I-A/I-E | 107632 |
| PE/Cyanine7 anti-mouse CD11c | 117317 |
| APC anti-mouse F4/80 | 123115 |
| PE anti-mouse CD170 (Siglec-F) | 155505 |
| Alexa Fluor® 700 anti-mouse Ly-6G | 127622 |

**Supplementary Figure S1 to Figure S50**


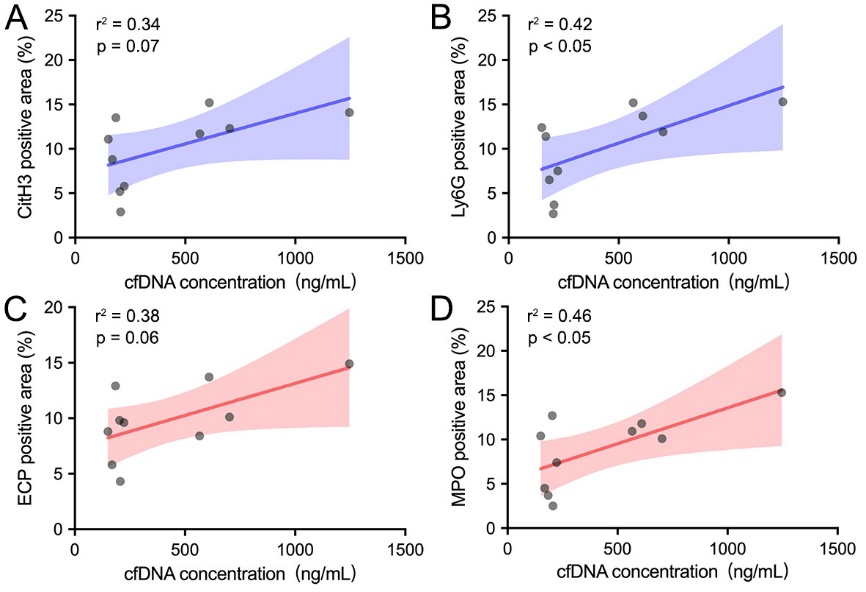


**Figure S1. The relationship between cfDNA and NET/EET levels in CRSwNP patients.** (A-D) The correlation between cfDNA concentration in nasal secretions and (A) CitH3 level, (B) Ly6G level, (C) ECP level, and (D) MPO level in the nasal polyps of CRSwNP patients.


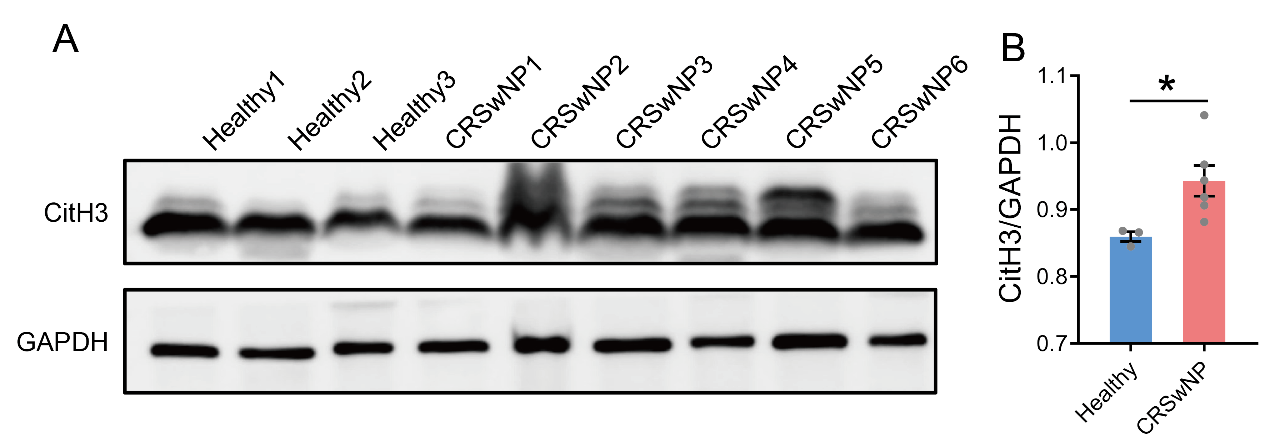


**Figure S2.** **Elevated CitH3 expression in nasal polyps of CRSwNP patients.** (A) Representative WB results showing CitH3 protein levels in nasal mucosa from healthy volunteers (n=3) versus nasal polyps from CRSwNP patients (n=6). (B) Densitometric quantification of CitH3 expression normalized to loading control. Data are presented as mean ± SEM (n = 3 or 6, Student’s t-test, two-tailed, * *P*<0.05).


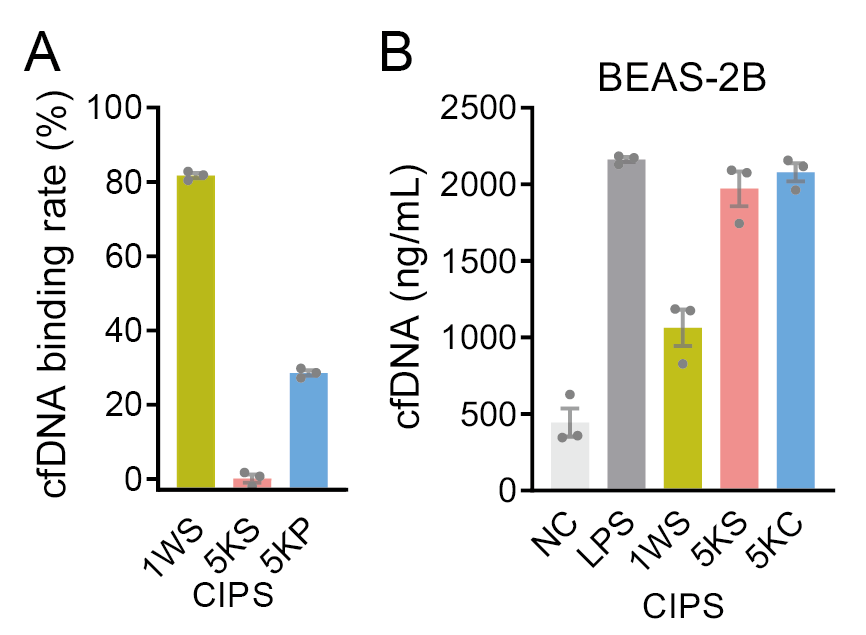


**Figure S3.** (A) cfDNA binding efficiency of CIPS 1WS, CIPS 5KS, and CIPS 5KP. (B) cfDNA released from LPS-stimulated BEAS-2B cells after incubation with CIPS 1WS, CIPS 5KS, and CIPS 5KP. Data are presented as mean ± SEM (n = 3).


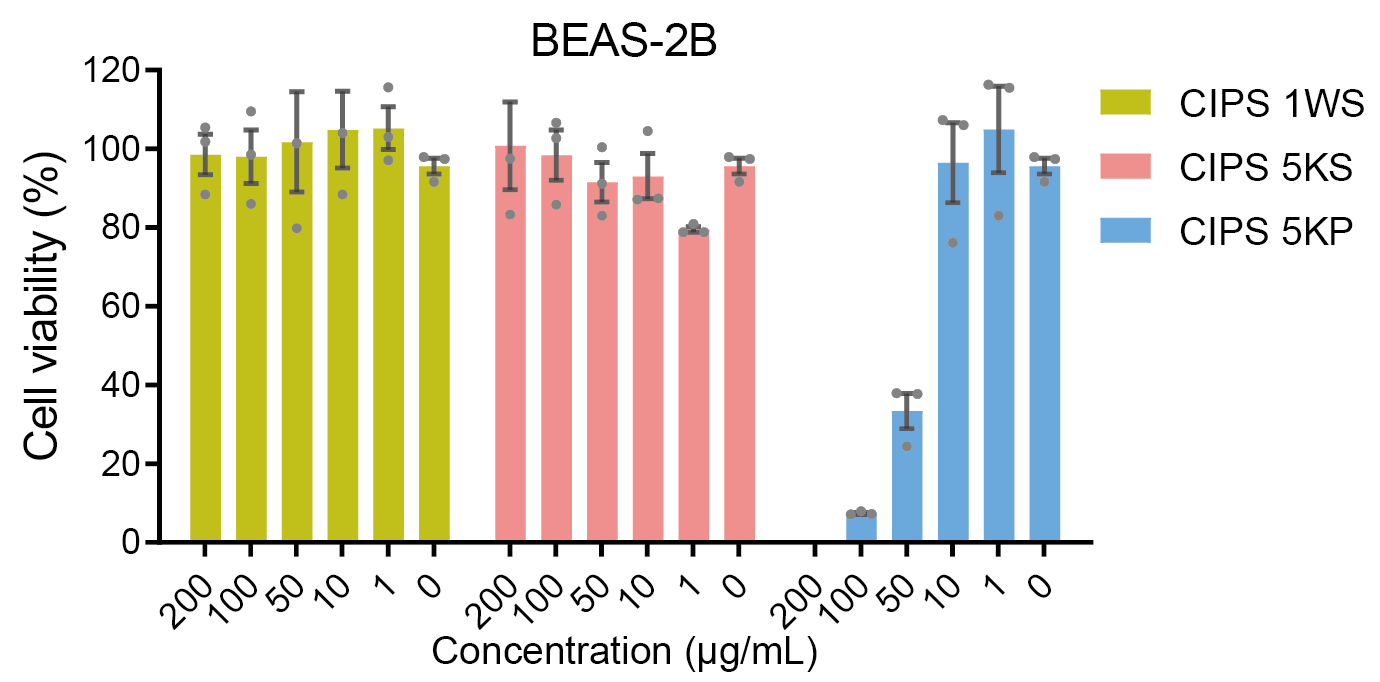


**Figure S4.** The viability of BEAS-2B cells treated with CIPS 1WS, CIPS 5KS, and CIPS 5KP with a series of concentrations for 24 h. Data are presented as mean ± SEM (n = 3).


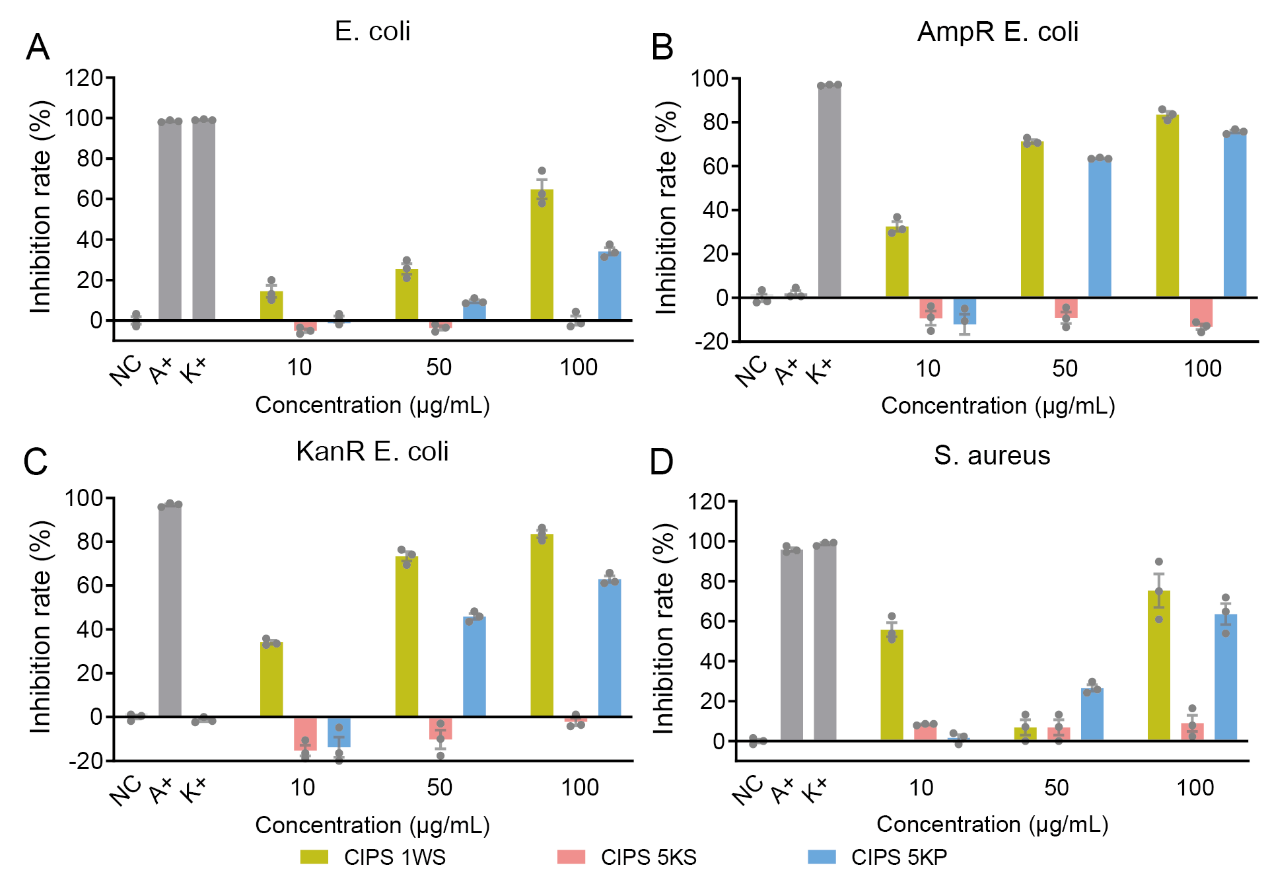


**Figure S5. Anti-bacteria effect of CIPS 1WS, CIPS 5KS, and CIPS 5KP.** The inhibition rate of (A) E. coli, (B) AmpR E. coli, (C) KanR E. coli, and (D) S. aureus after incubation with CIPS 1WS, CIPS 5KS, and CIPS 5KP with a series of concentrations (10 μg/mL to 100 μg/mL). Data are presented as mean ± SEM (n = 3).


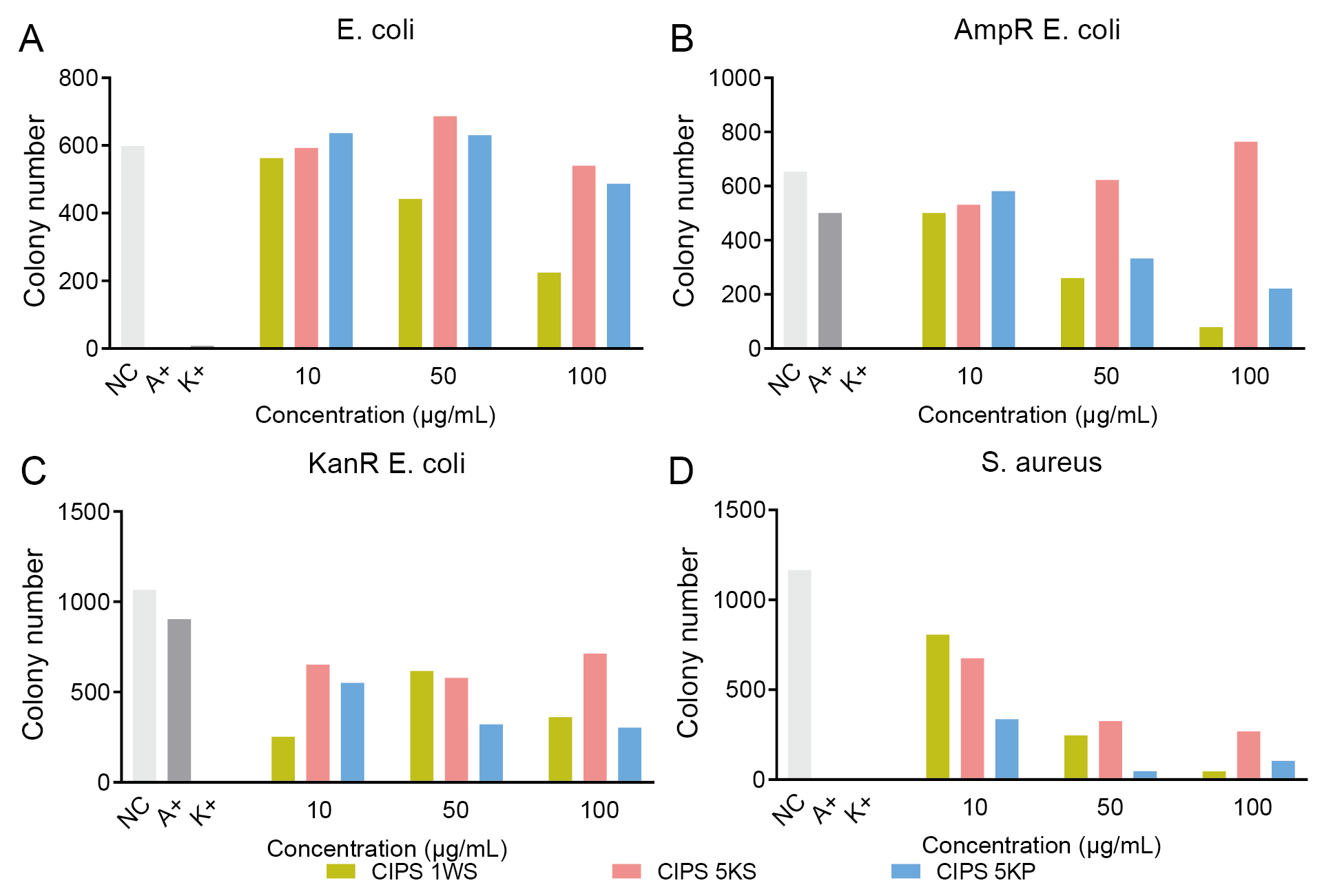


**Figure S6.** Bacterial agar plate colonies count of (A) E. coli, (B) AmpR E. coli, (C) KanR E. coli, and (D) S. aureus after incubation with CIPS 1WS, CIPS 5KS, and CIPS 5KP.


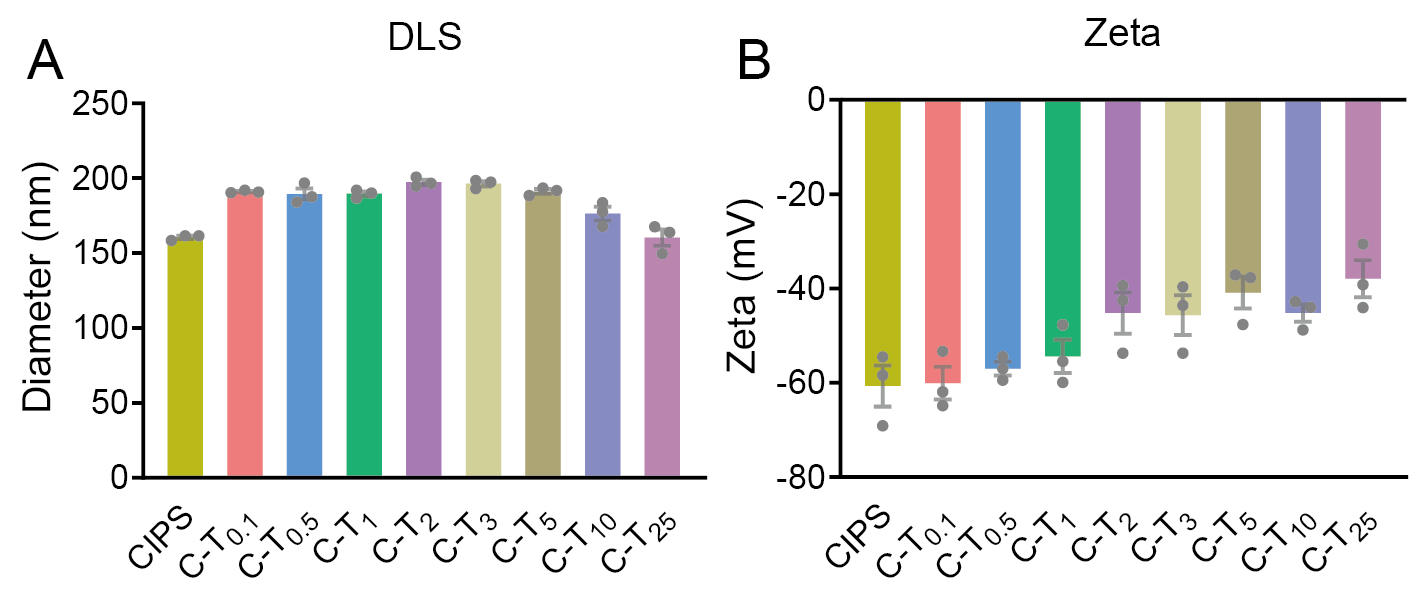


**Figure S7.** (A) Size and (B) Zeta potential of CIPS, C-TA_0.1_, C-TA_0.5_, C-TA_1_, C-TA_2_, C-TA_3_, C-TA_5_, C-TA_10_, and C-TA_25_. Data are presented as mean ± SEM (n = 3).


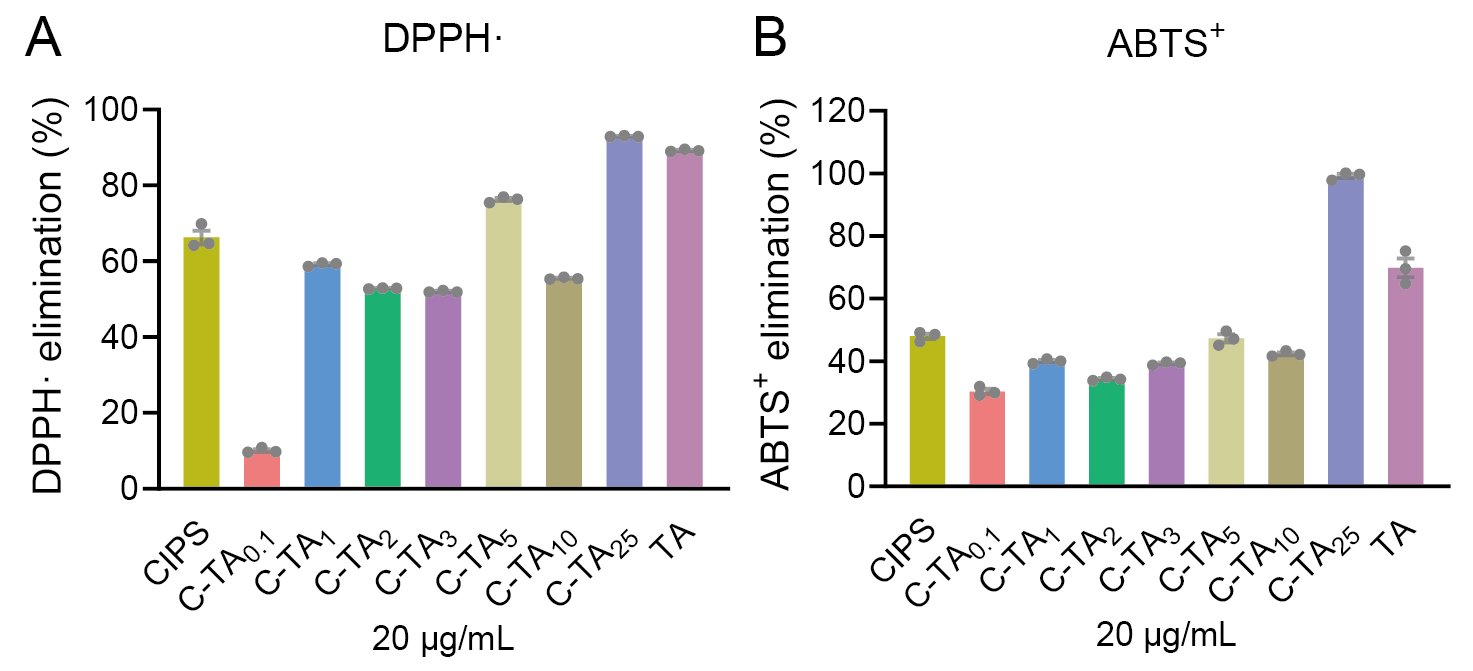


**Figure S8.** RONS reduction capacities of CIPS, C-TA_0.1_, C-TA_1_, C-TA_2_, C-TA_3_, C-TA_5_, C-TA_10_, C-TA_25_, and TA to (A) DPPH·, and (B) ABTS^+^. Data are presented as mean ± SEM (n = 3).


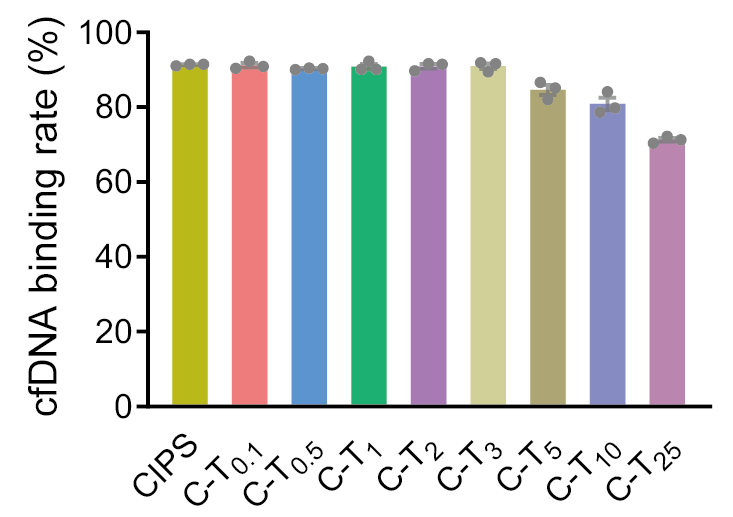


**Figure S9.** cfDNA binding efficiency of CIPS, C-TA_0.1_, C-TA_0.5_, C-TA_1_, C-TA_2_, C-TA_3_, C-TA_5_, C-TA_10_, and C-TA_25_. Data are presented as mean ± SEM (n = 3).


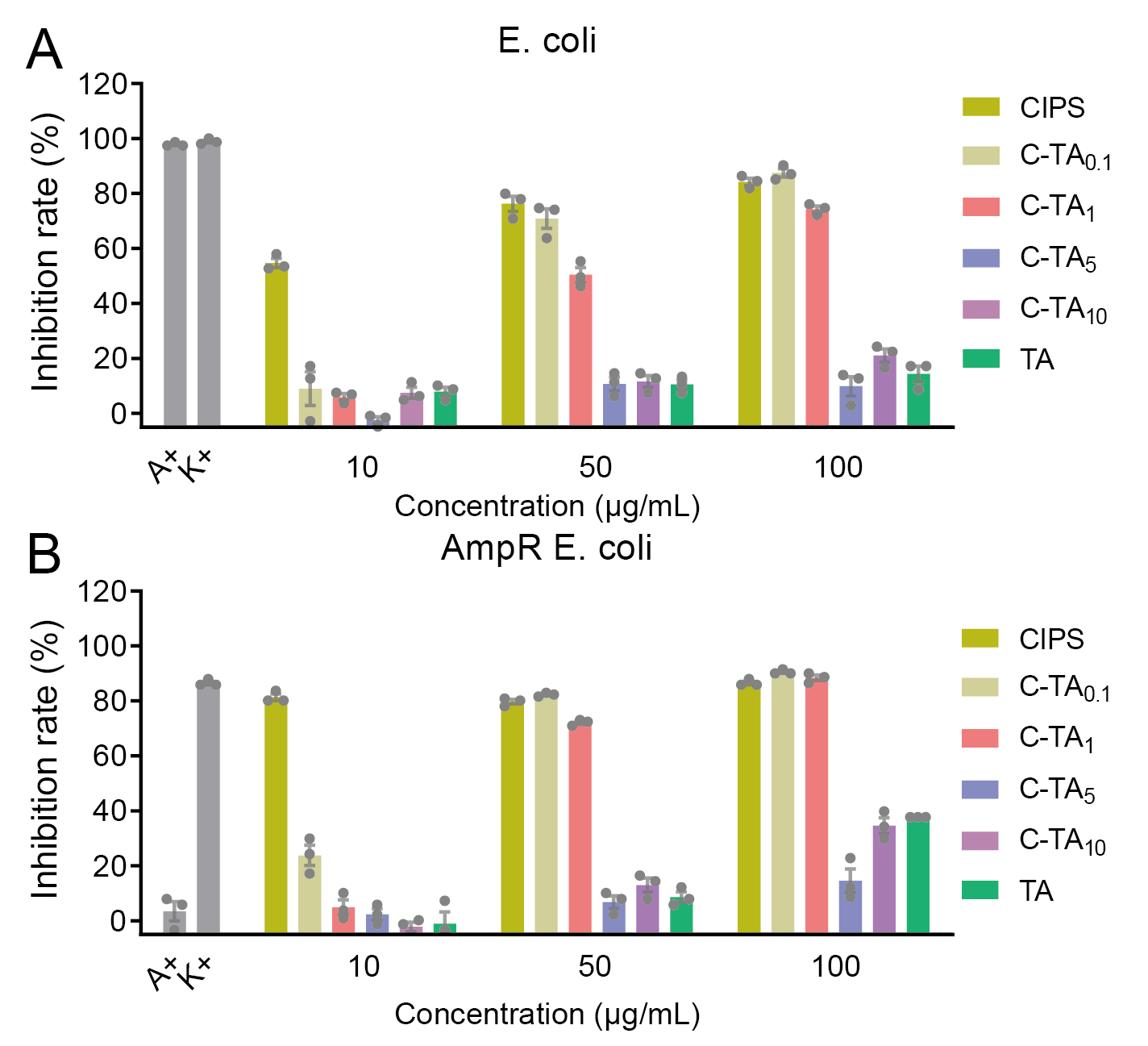


**Figure S10.** Anti-bacteria effect of TA-covered CIPS nanosheets. The inhibition rate of (A) E. coli, and (B) AmpR E. coli incubation with CIPS, C-TA_0.1_, C-TA_1_, C-TA_5_, C-TA_10_, and TA with a series of concentrations (10 μg/mL to 100 μg/mL). Data are presented as mean ± SEM (n = 3).


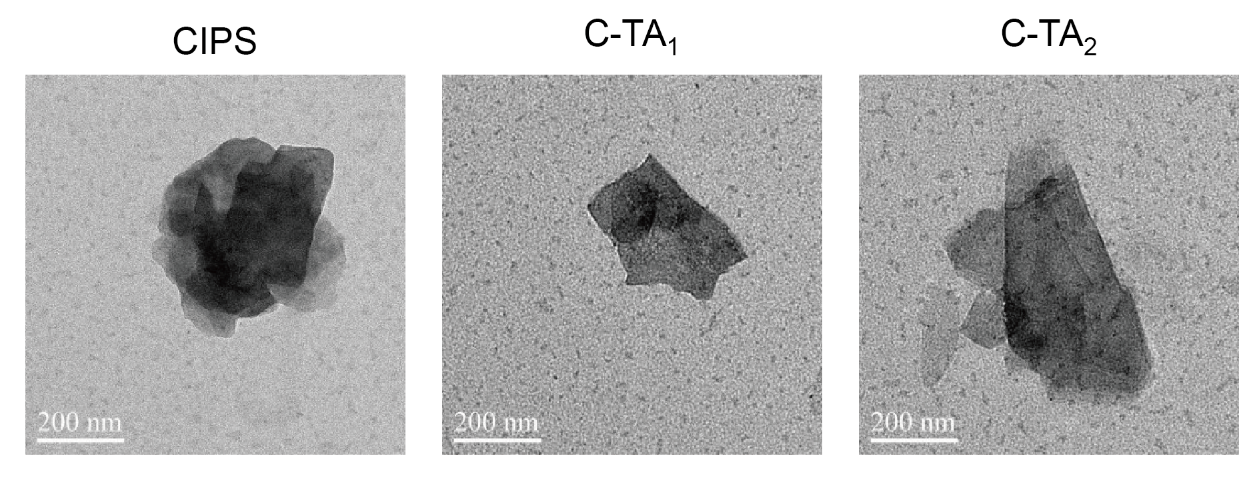


**Figure S11.** Representative TEM images of CIPS, C-TA_1_, and C-TA_2_. Scale bars: 200 nm.


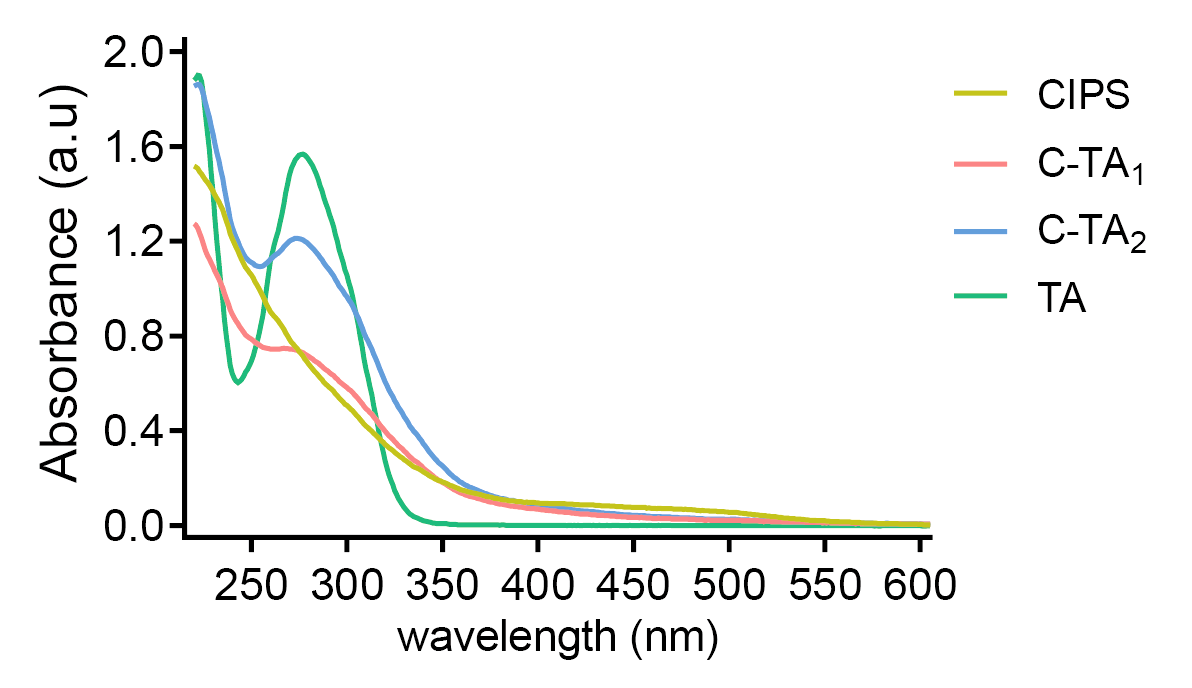


**Figure S12.** The UV-vis absorbance spectra of CIPS, C-TA_1_, C-TA_2_, and TA.


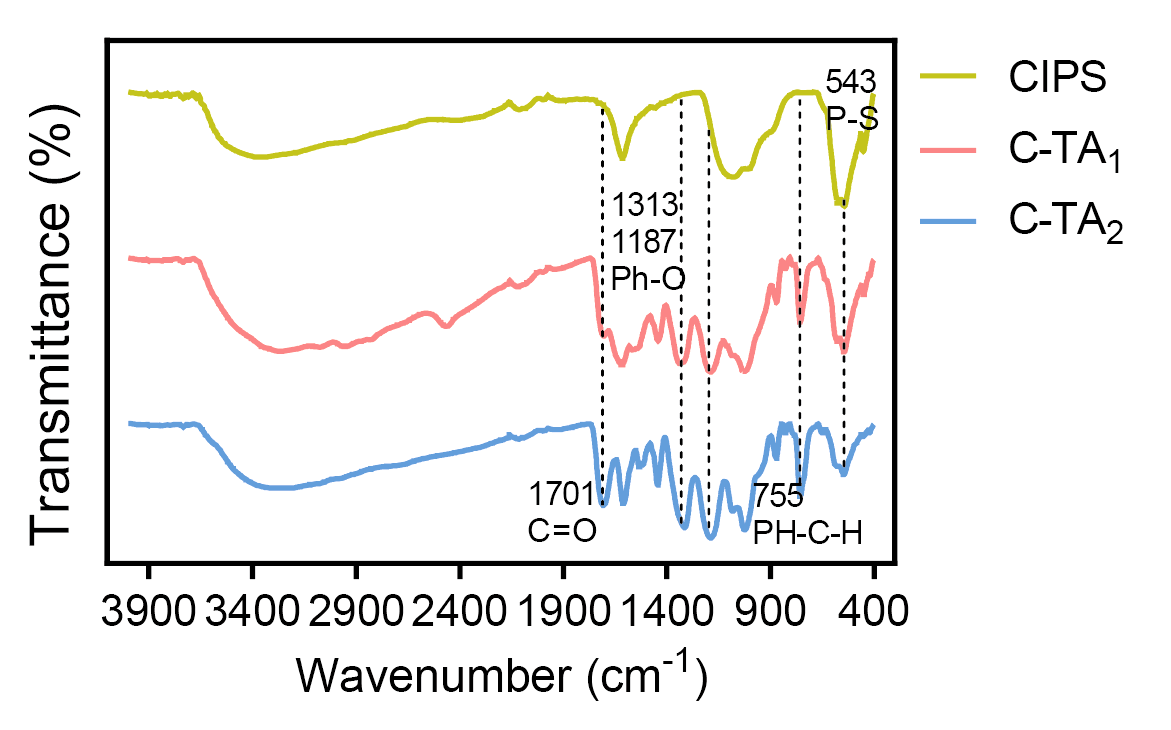


**Figure S13.** The FTIR spectra of CIPS, C-TA_1_, and C-TA_2_.


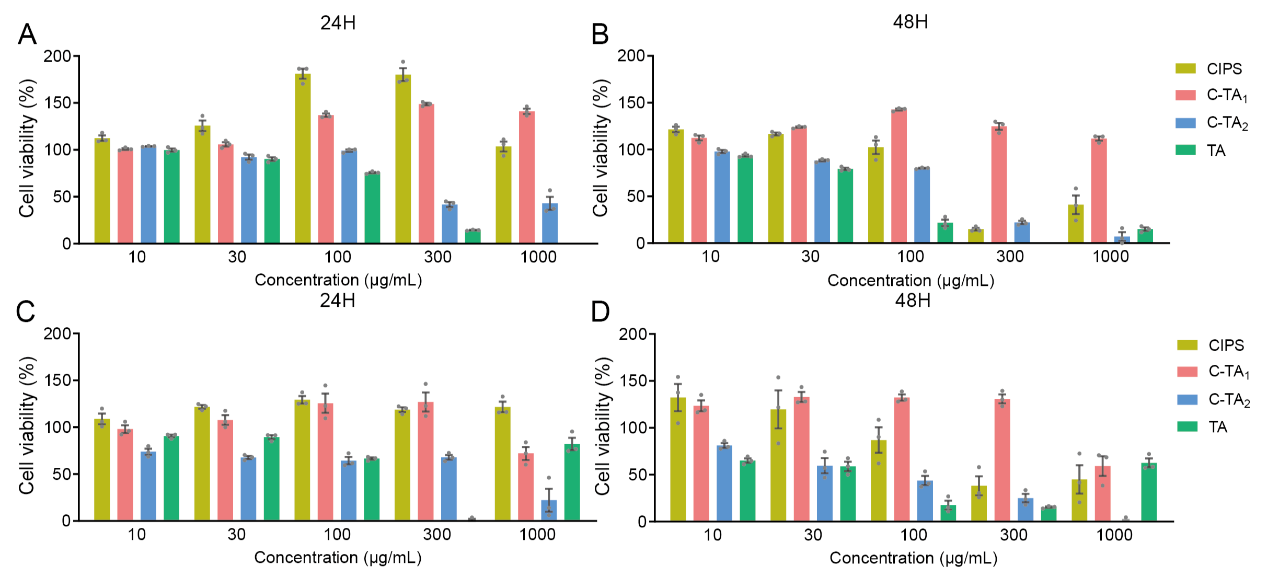


**Figure S14.** Viability of BEAS-2B cells treated with CIPS, C-TA_1_, C-TA_2_, and TA with a series of concentrations for (A) 24 h and (B) 48 h. Viability of RPMI2650 cells treated with CIPS, C-TA_1_, C-TA_2_, and TA with a series of concentrations for (C) 24 h and (D) 48 h. Data are presented as mean ± SEM (n = 3).


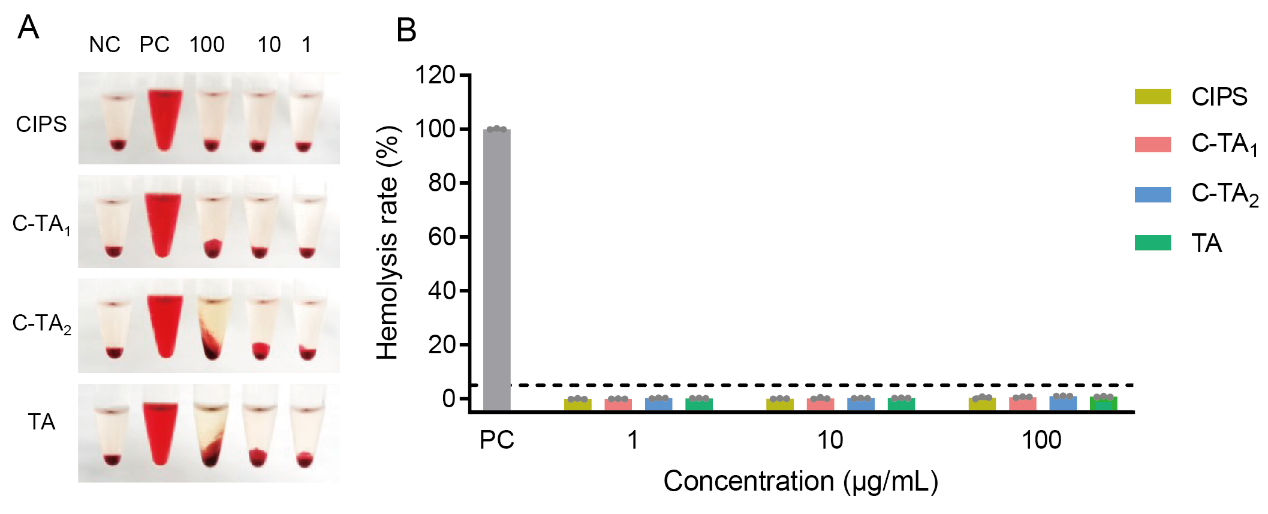


**Figure S15.** (A) Hemolytic assay of nanosheets after incubation with red blood cells. (B) Hemolysis rate calculated by OD540 value. Data are presented as mean ± SEM (n = 3).


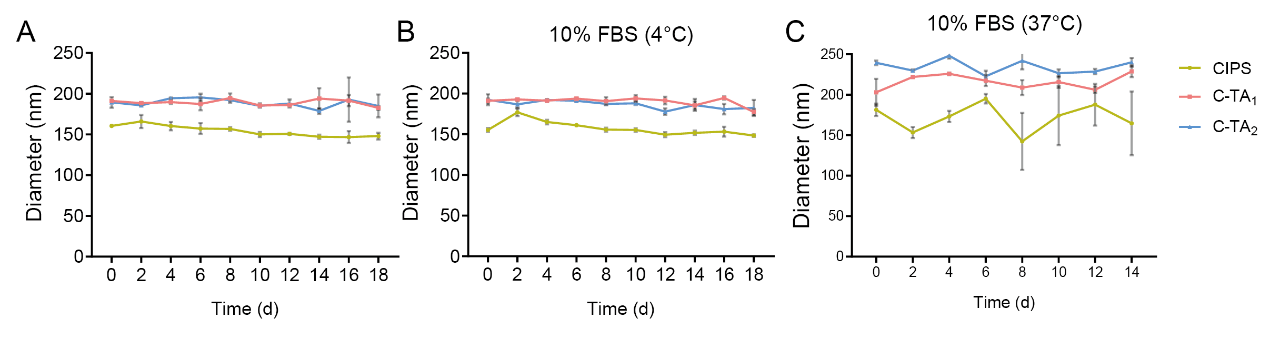


**Figure S16.** Stability of CIPS, C-TA_1_, and C-TA_2_. Size changes of CIPS, C-TA_1_, and C-TA_2_ (A) in ddH_2_O and (B) in 10% FBS at 4**°C** within 18 d, and (C) in 10% FBS at 37**°C** within 14 d. Data are presented as mean ± SEM (n = 3).


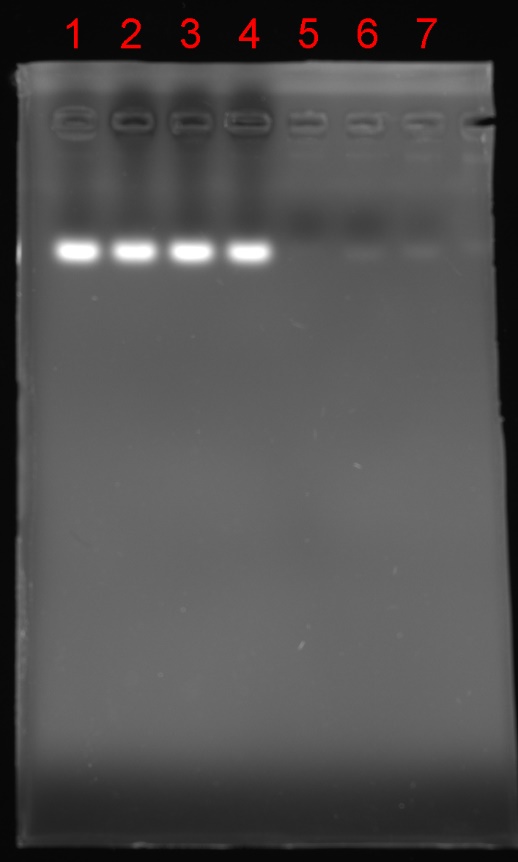


**Figure S17.** The agarose gel electrophoresis of cfDNA with CIPS, C-TA_1_, and C-TA_2_. lane 1: positive control, lane 2: cfDNA with CIPS, lane 3: cfDNA with C-TA_1_, lane 4: cfDNA with C-TA_2_, lane 5: The supernatant of cfDNA with CIPS, lane 6: The supernatant of cfDNA with C-TA_1_, lane 7: The supernatant of cfDNA with C-TA_2_.


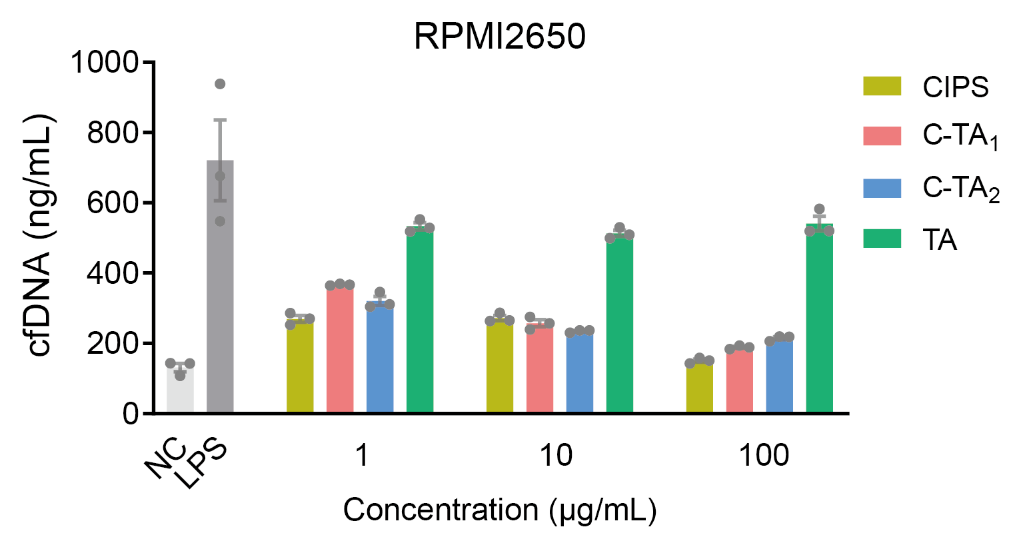


**Figure S18.** cfDNA generation by LPS-stimulated RPMI2650 cells after incubation with CIPS, C-TA_1_, C-TA_2_, and TA with a series of concentrations (1 μg/mL to 100 μg/mL). Data are presented as mean ± SEM (n = 3).


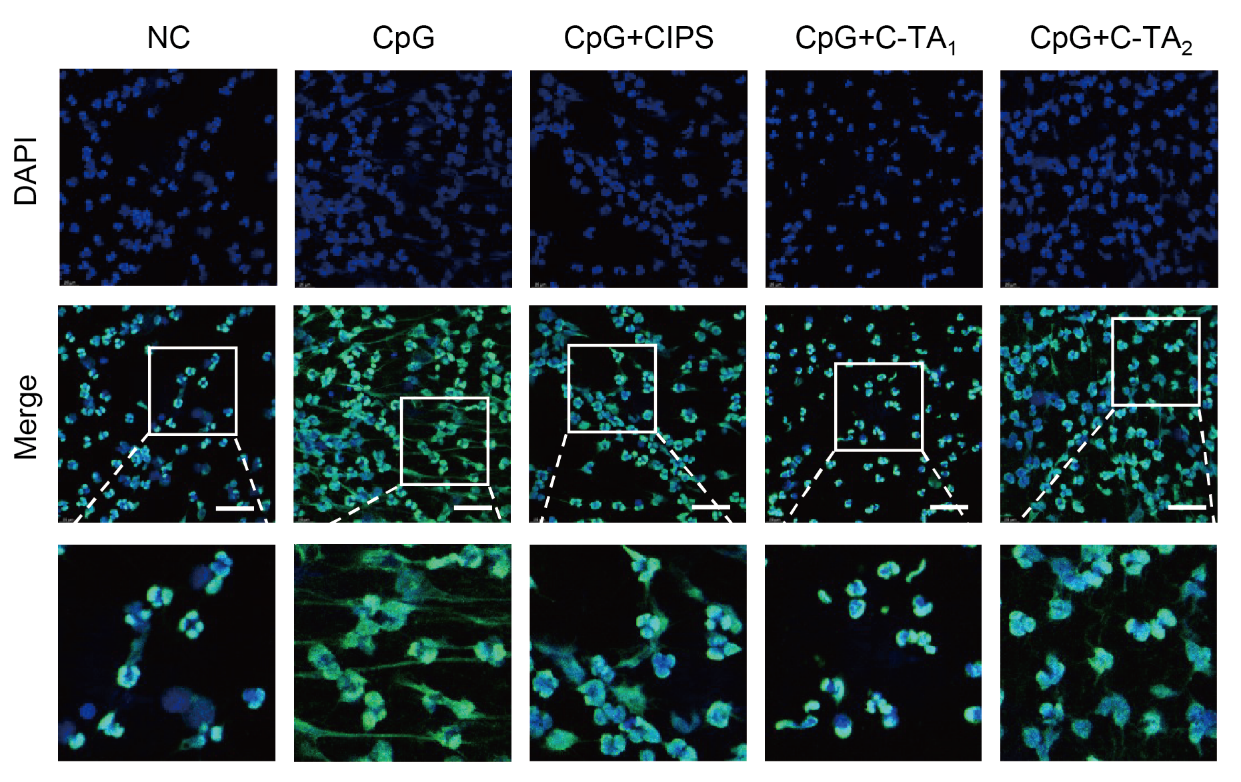


**Figure S19.** Representative DAPI and CitH3 co-staining images of the CpG-treated neutrophils following incubation with CIPS, C-TA_1_, and C-TA_2_, along with a magnified view of the squared region. Scale bars: 50 μm.


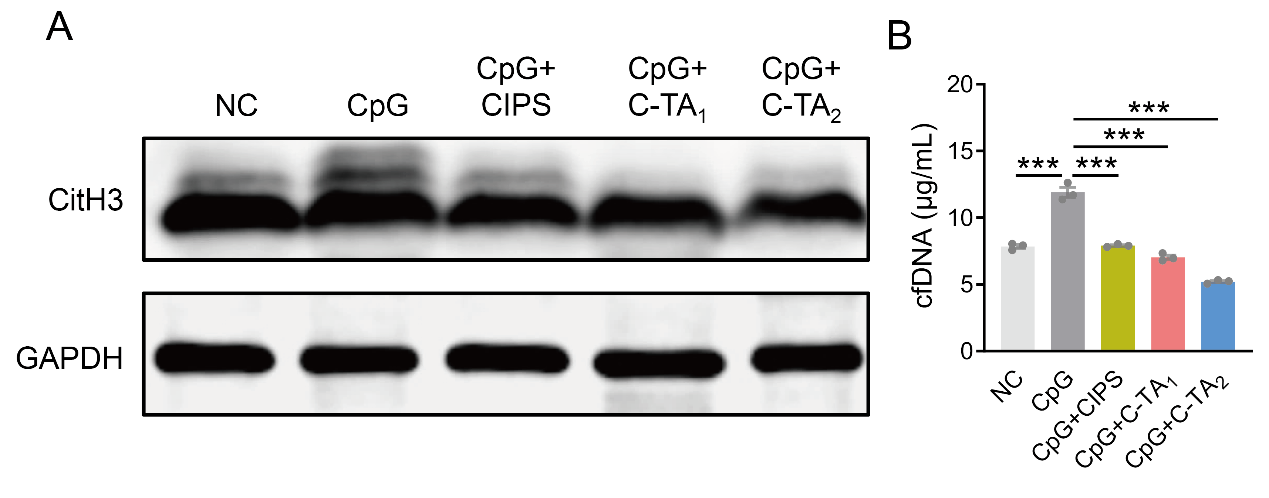


**Figure S20.** **Modulation of NETosis by nanosheets.** (A) WB analysis of CitH3 expression in CpG-stimulated neutrophils following treatment with CIPS, C-TA_1_, and C-TA_2_. (B) Quantification of cfDNA release from CpG-stimulated neutrophils after incubation with CIPS, C-TA_1_, and C-TA_2_. Data are presented as mean ± SEM (n = 3, Student’s t-test, two-tailed, *** *P*<0.001).


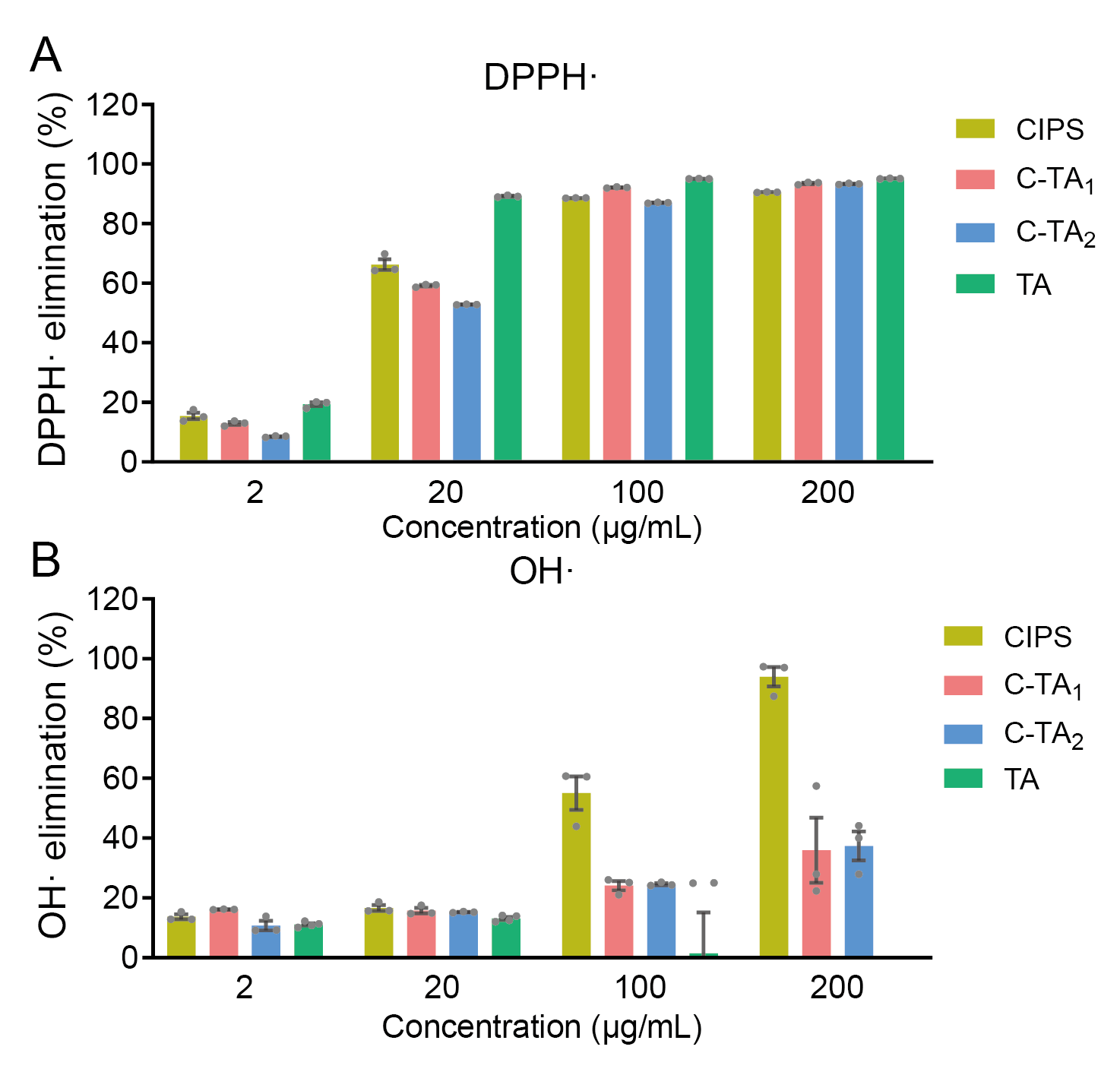


**Figure S21.** The antioxidant capacities of CIPS, C-TA_1_, C-TA_2_, and TA to (A) DPPH·, and (B) OH·. Data are presented as mean ± SEM (n = 3).


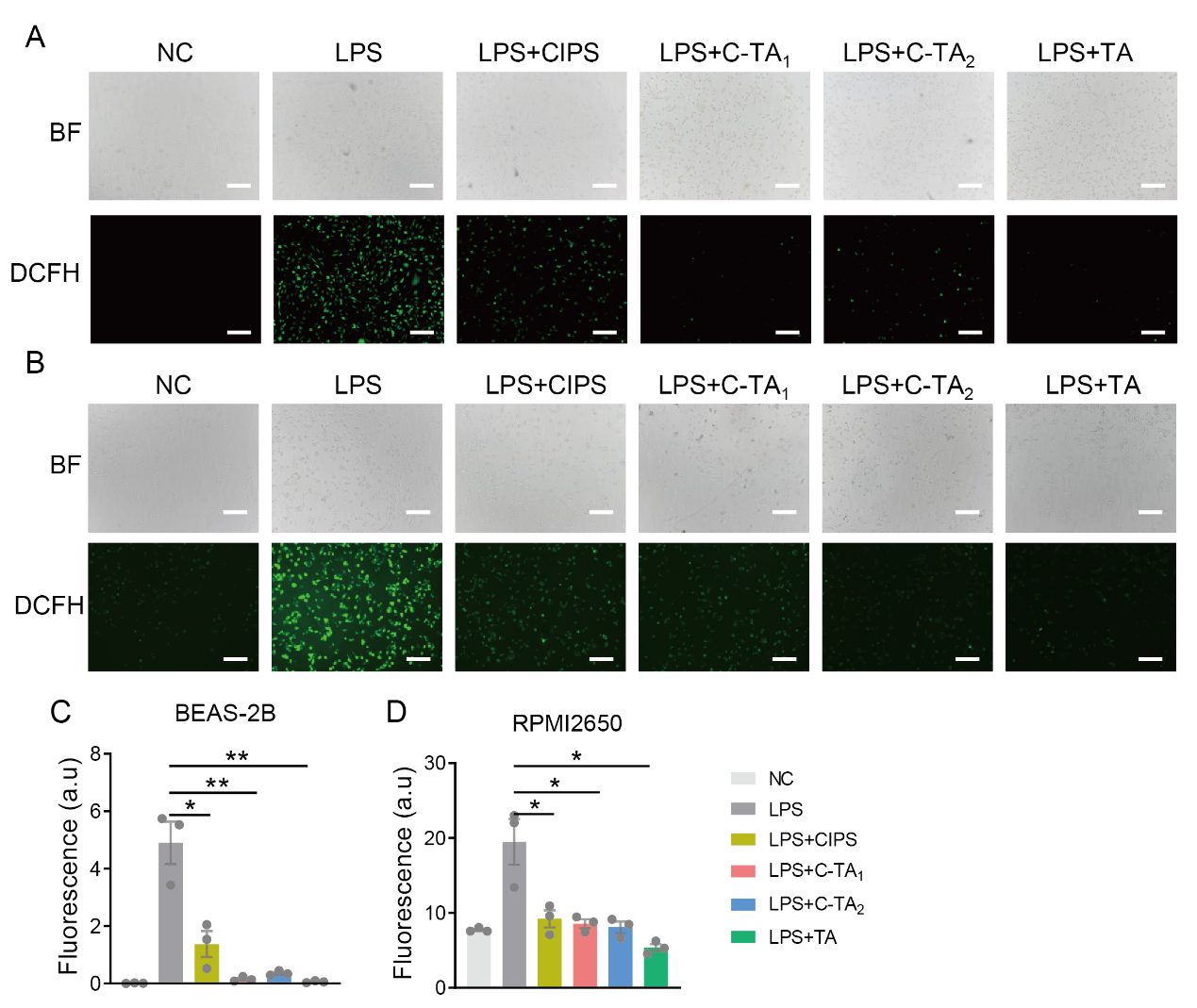


**Figure S22.** (A) Representative DCFH-DA fluorescent images of the LPS-treated BEAS-2B cells after incubation with CIPS, C-TA_1_, C-TA_2_, and TA. Scale bars: 200 μm. (B) Representative DCFH-DA fluorescent images of the LPS-treated RPMI2650 cells after incubation with CIPS, C-TA_1_, C-TA_2_, and TA. Scale bars: 200 μm. (C) Quantitative analysis of fluorescence in (A). (D) Quantitative analysis of fluorescence in (C). Data are presented as mean ± SEM (n = 3, Student’s t-test, two-tailed, * *P*<0.05, ** *P*<0.01).


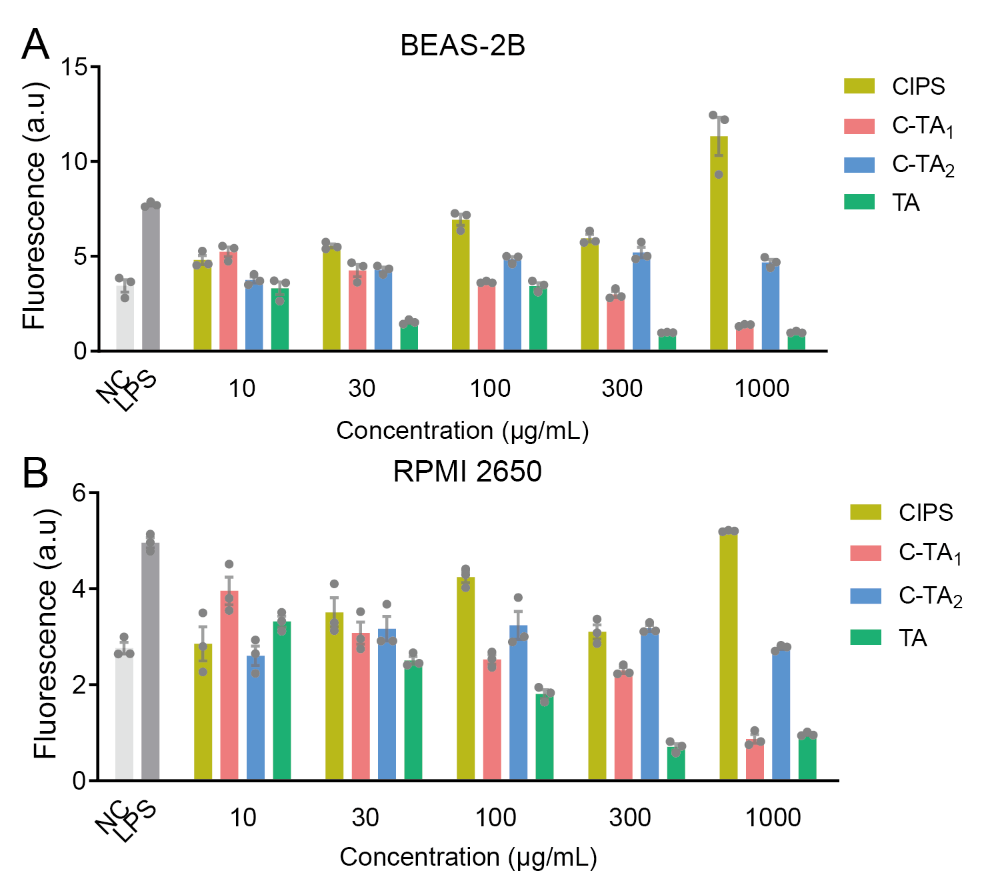


**Figure S23.** DCFH-DA fluorescence value in LPS-treated (A) BEAS-2B cells, (B) RPMI2650 cells with CIPS, C-TA_1_, C-TA_2_, and TA. Data are presented as mean ± SEM (n = 3).


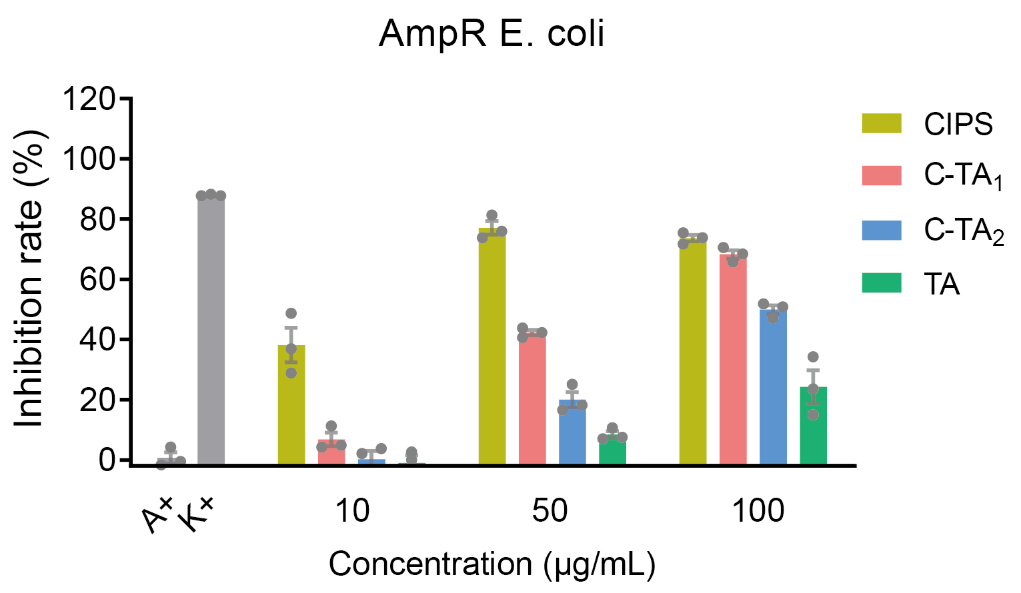


**Fig S24.** The inhibition rate of AmpR E. coli after incubation with CIPS, C-TA_1_, C-TA_2_, and TA. Data are presented as mean ± SEM (n = 3).


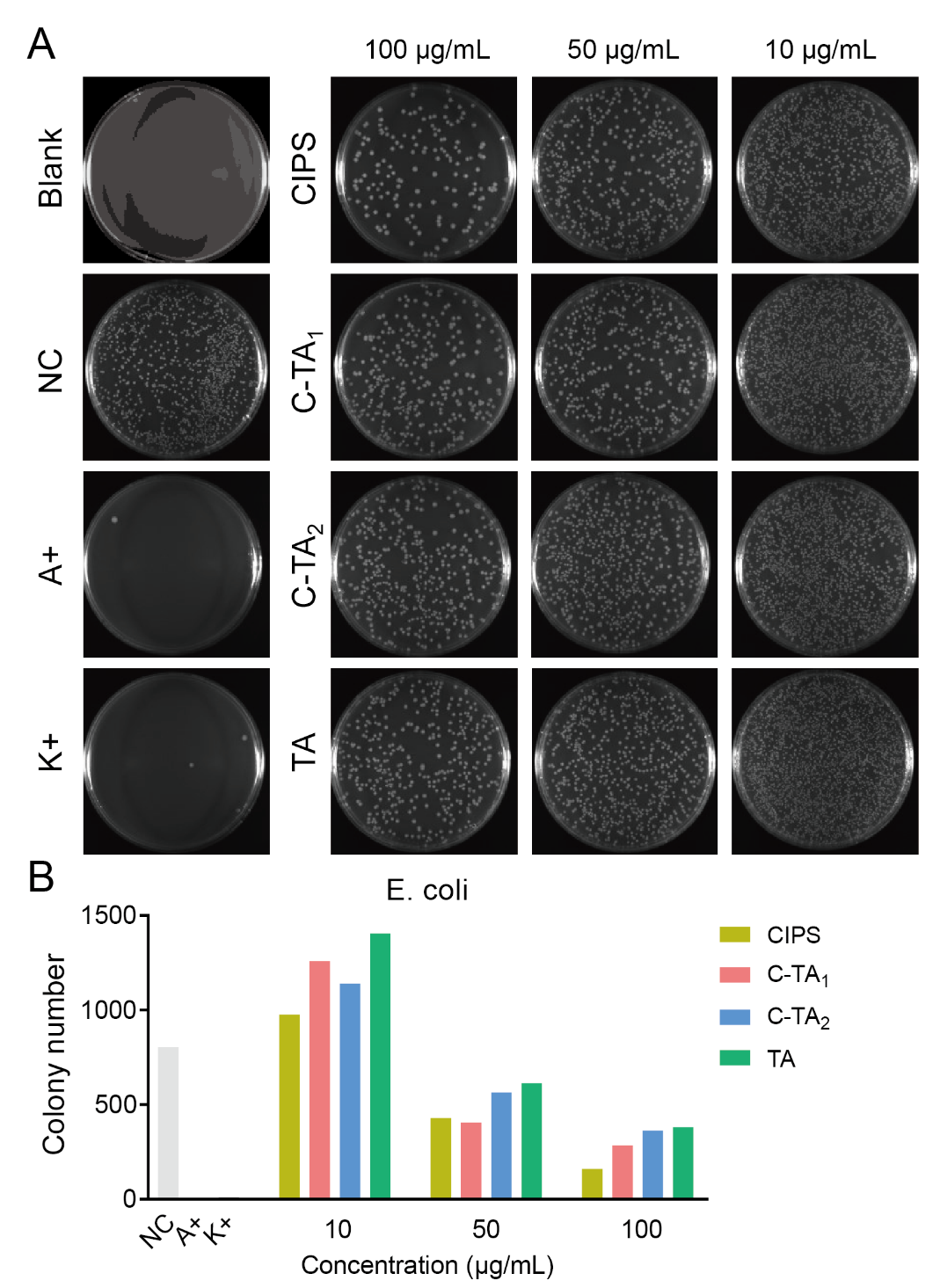


**Fig S25.** (A) Photographs of bacterial colonies formed by E. coli treated with CIPS, C-TA_1_, C-TA_2_, and TA with a series of concentrations (10 μg/mL to 100 μg/mL). (B) Colony count of E. coli in (A).


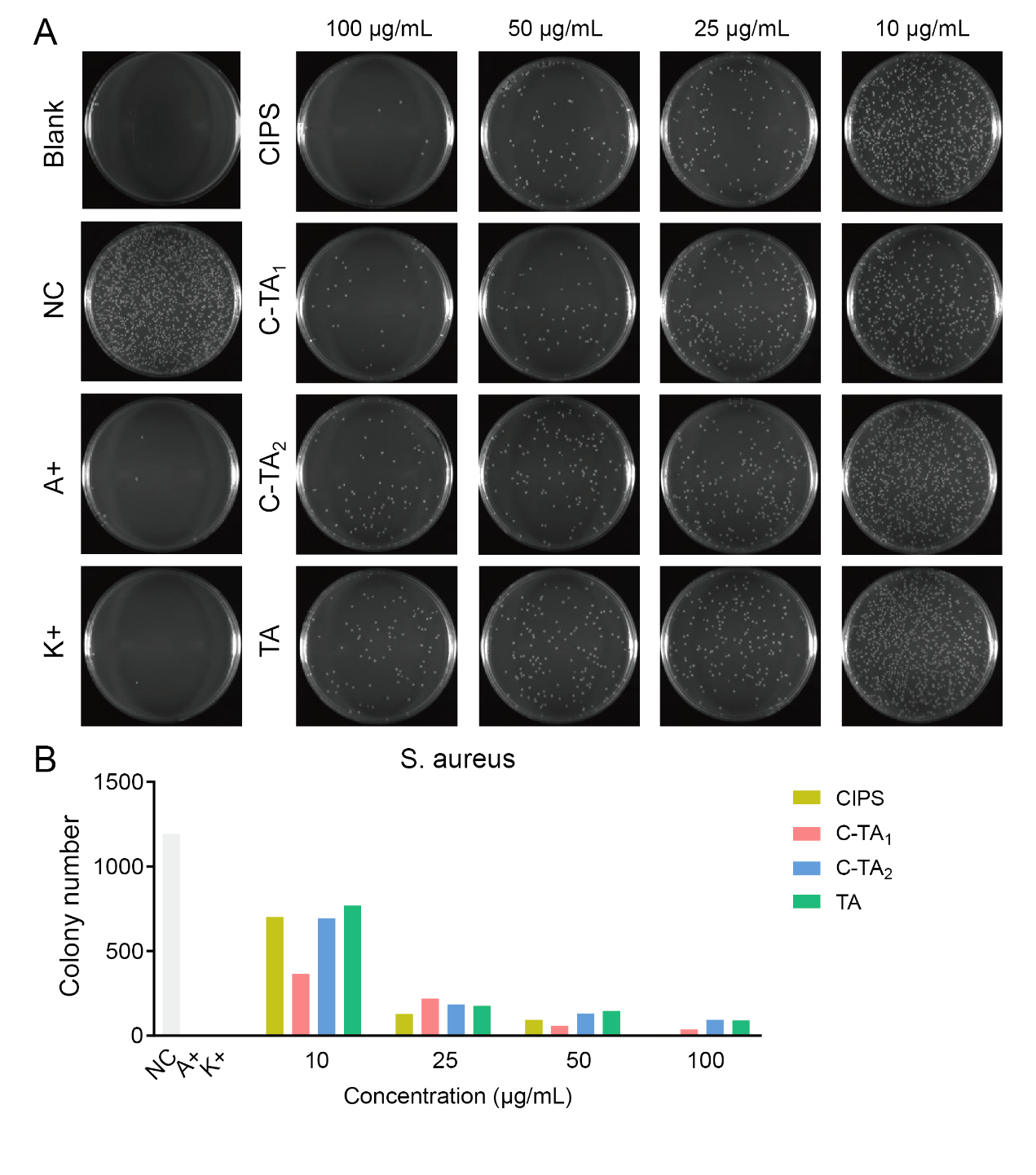


**Fig S26.** (A) Photographs of bacterial colonies formed by S. aureus treated with CIPS, C-TA_1_, C-TA_2_, and TA with a series of concentrations (10 μg/mL to 100 μg/mL). (B) Colony count of S. aureus in (A).


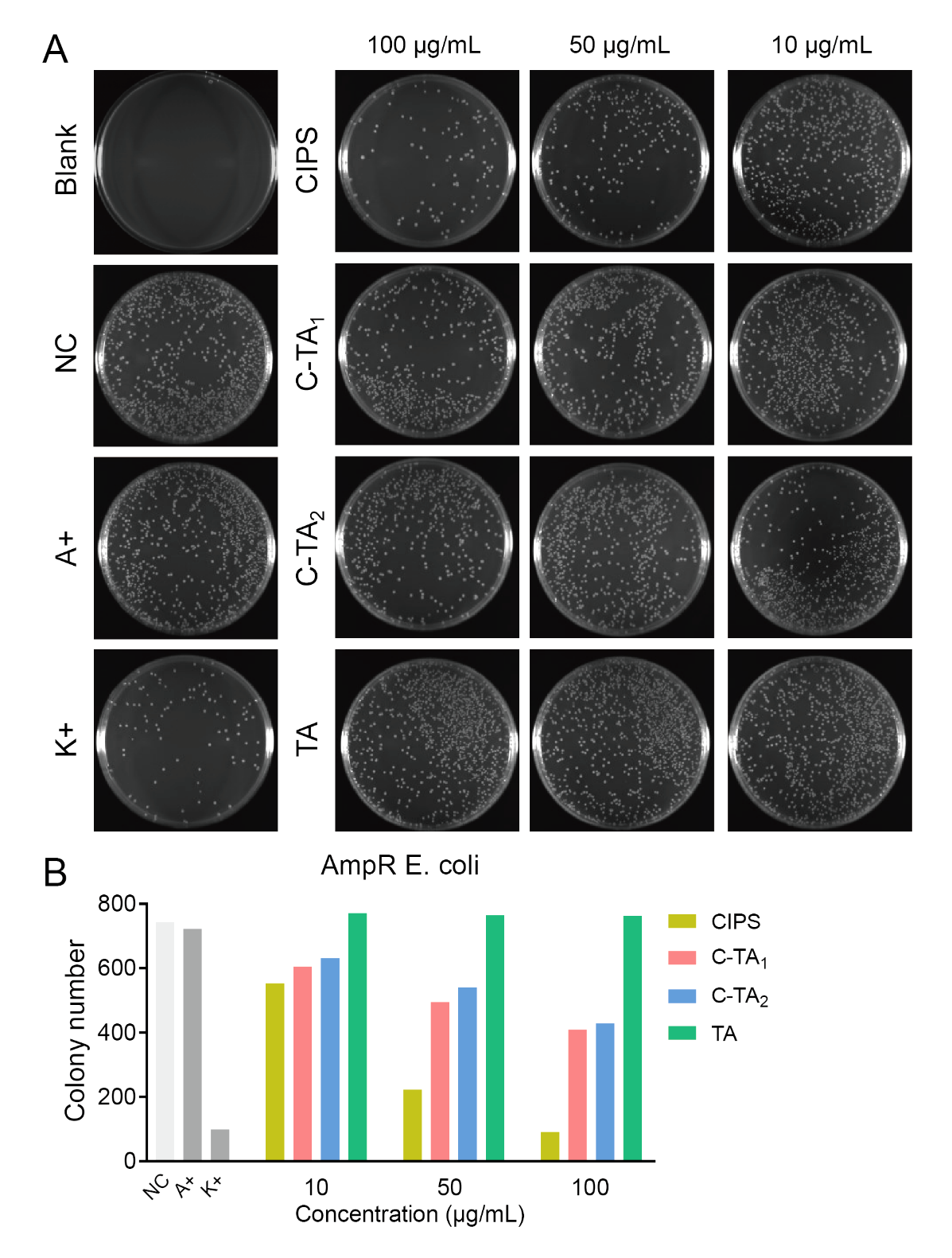


**Figure S27.** (A) Photographs of bacterial colonies formed by AmpR E. coli treated with CIPS, C-TA_1_, C-TA_2_, and TA with a series of concentrations (10 μg/mL to 100 μg/mL). (B) Colony count of AmpR E. coli in (A).


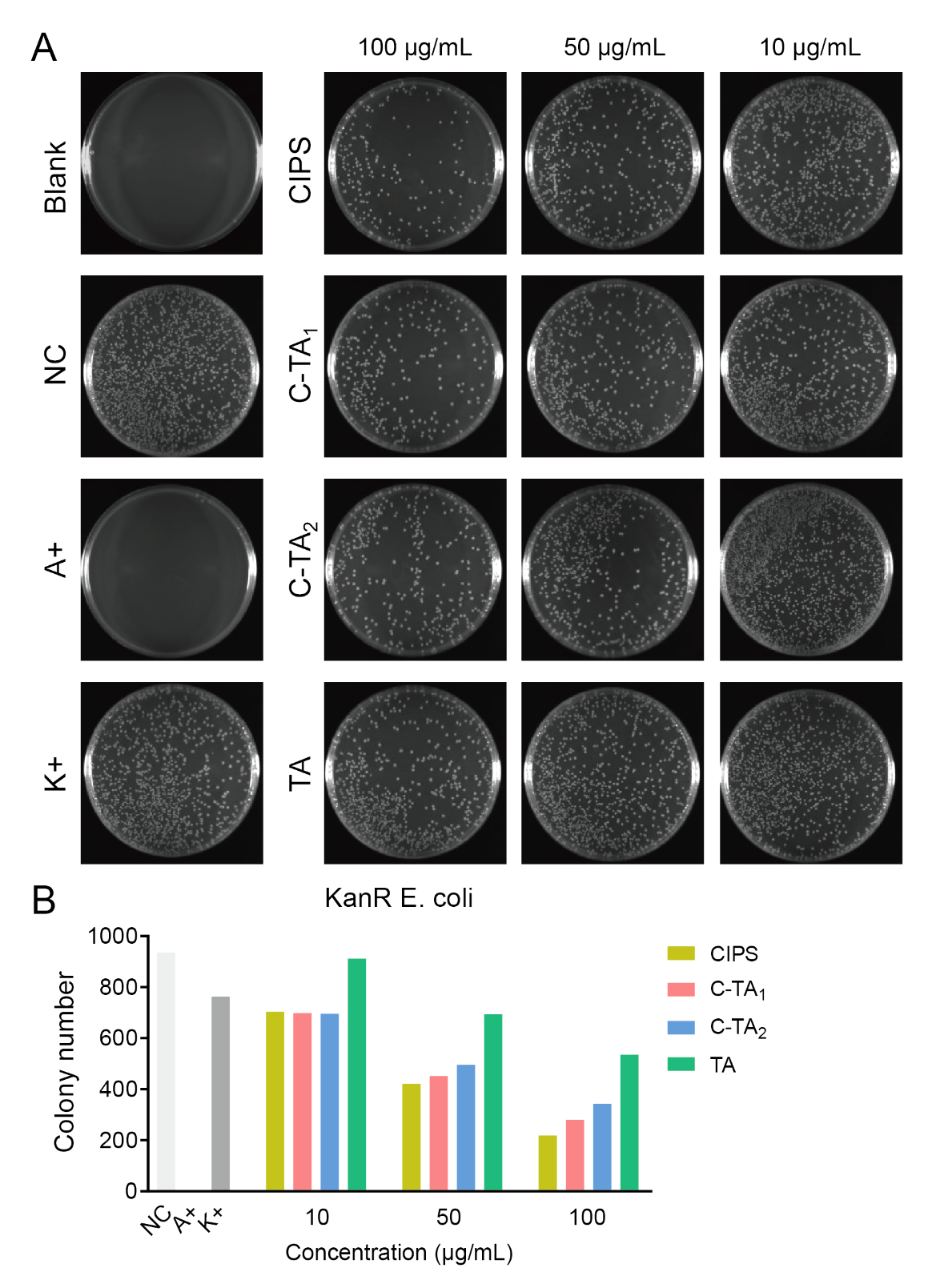


**Figure S28.** (A) Photographs of bacterial colonies formed by KanR E. coli treated with CIPS, C-TA_1_, C-TA_2_, and TA with a series of concentrations (10 μg/mL to 100 μg/mL). (B) Colony count of KanR E. coli in (A).


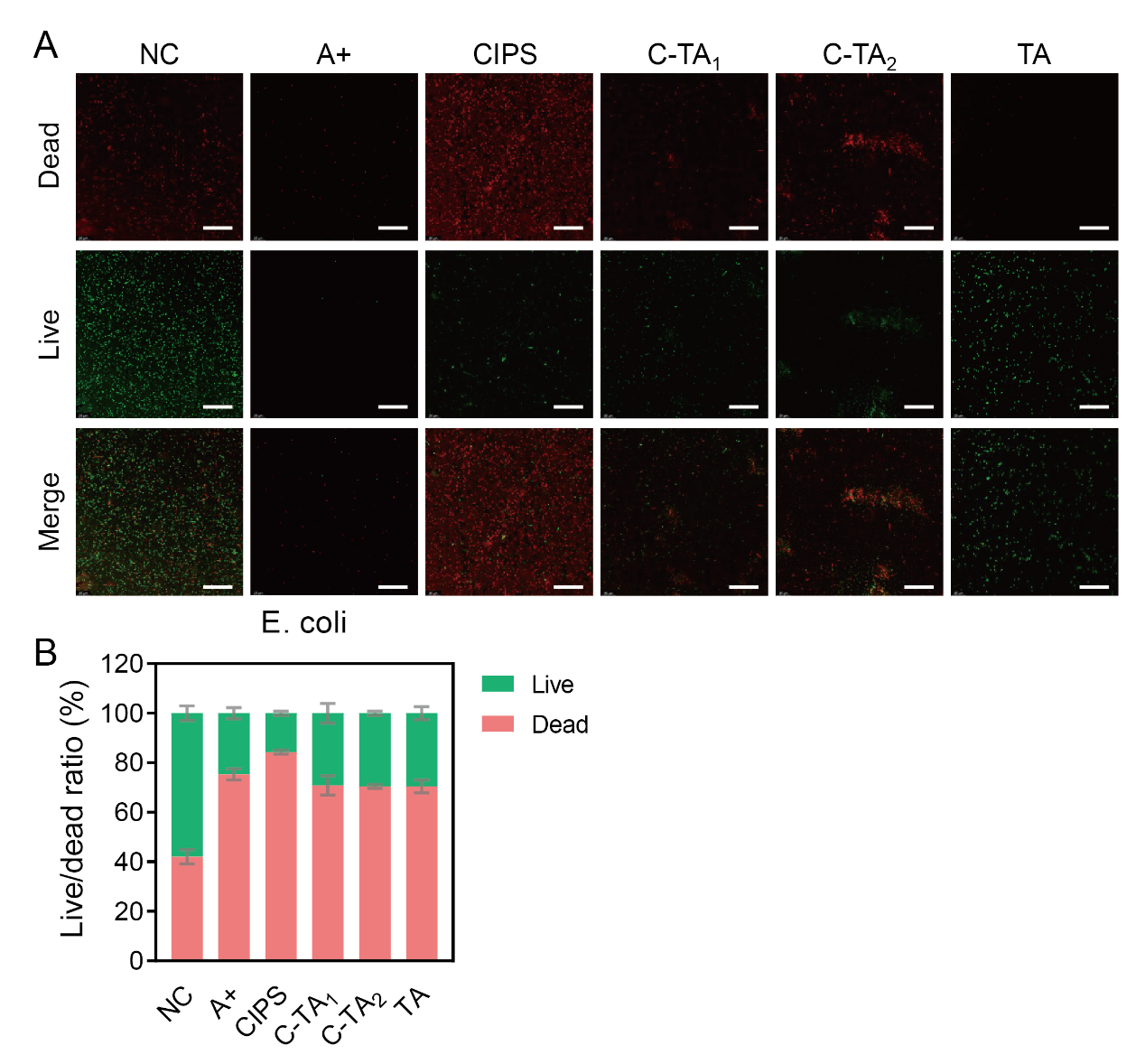


**Figure S29.** (A) Live/dead staining of E. coli treated with CIPS, C-TA_1_, C-TA_2_, and TA. Scale bars: 100 μm. (B) Quantitative analysis of fluorescence in (A). Data are presented as mean ± SEM (n = 3).


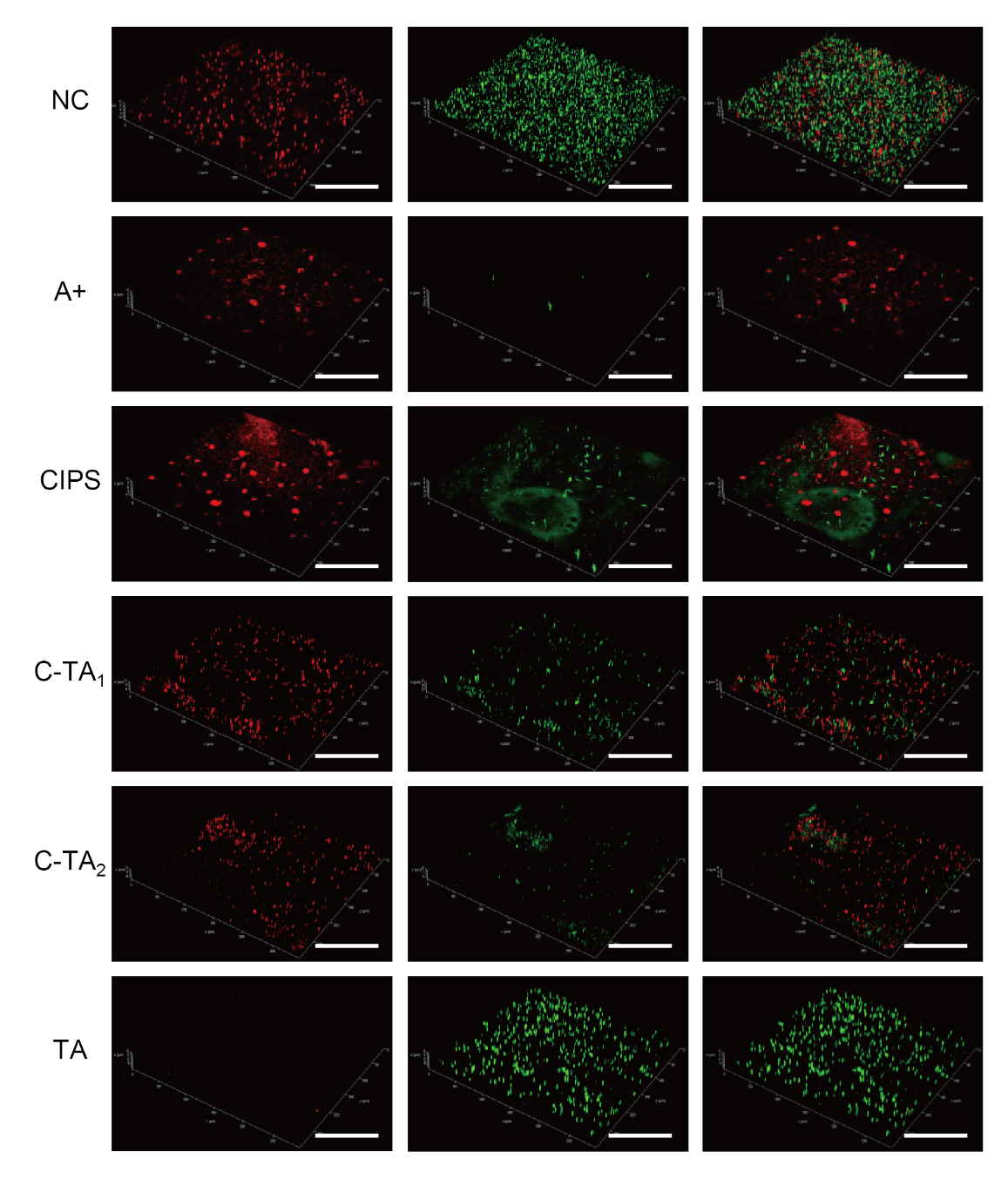


**Figure S30.** 3D images of live/dead staining of E. coli treated with CIPS, C-TA_1_, C-TA_2_, and TA. Scale bars: 100 μm.


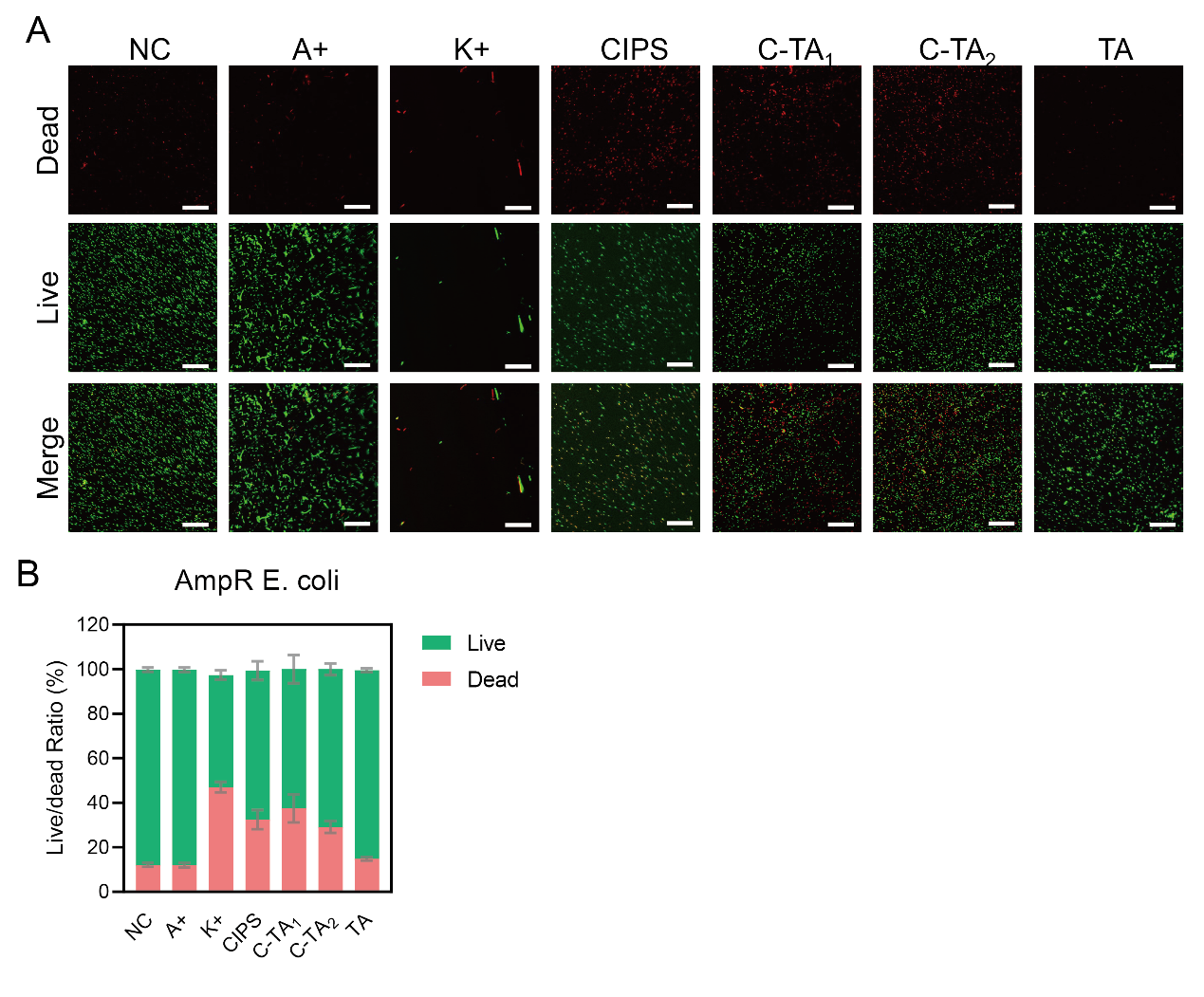


**Figure S31.** (A) Live/dead staining of AmpR E. coli treated with CIPS, C-TA_1_, C-TA_2_, and TA. Scale bars: 100 μm. (B) Quantitative analysis of fluorescence in (A). Data are presented as mean ± SEM (n = 3).


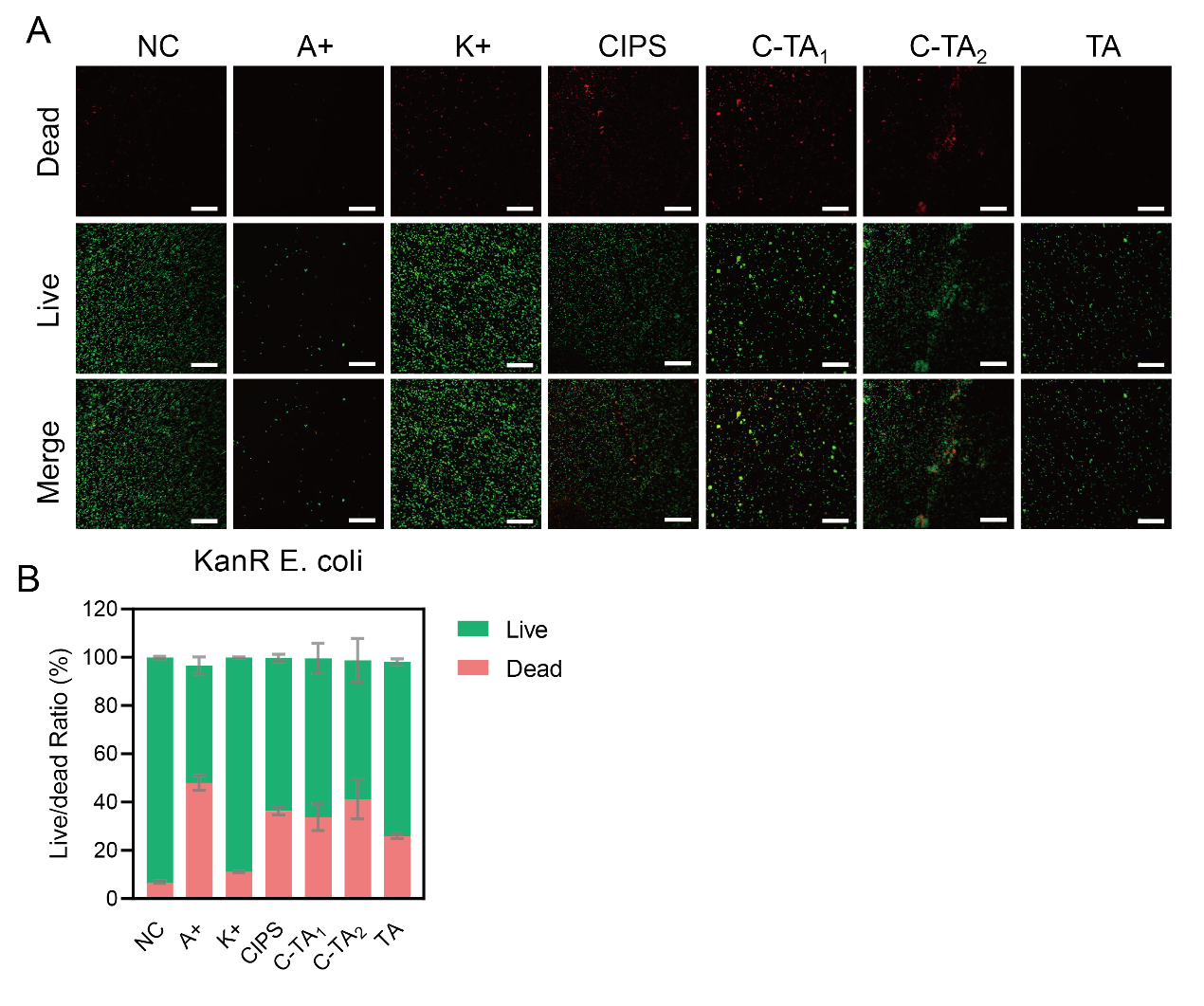


**Figure S32.** (A) Live/dead staining of KanR E. coli treated with CIPS, C-TA_1_, C-TA_2_, and TA. Scale bars: 100 μm. (B) Quantitative analysis of fluorescence in (A). Data are presented as mean ± SEM (n = 3).


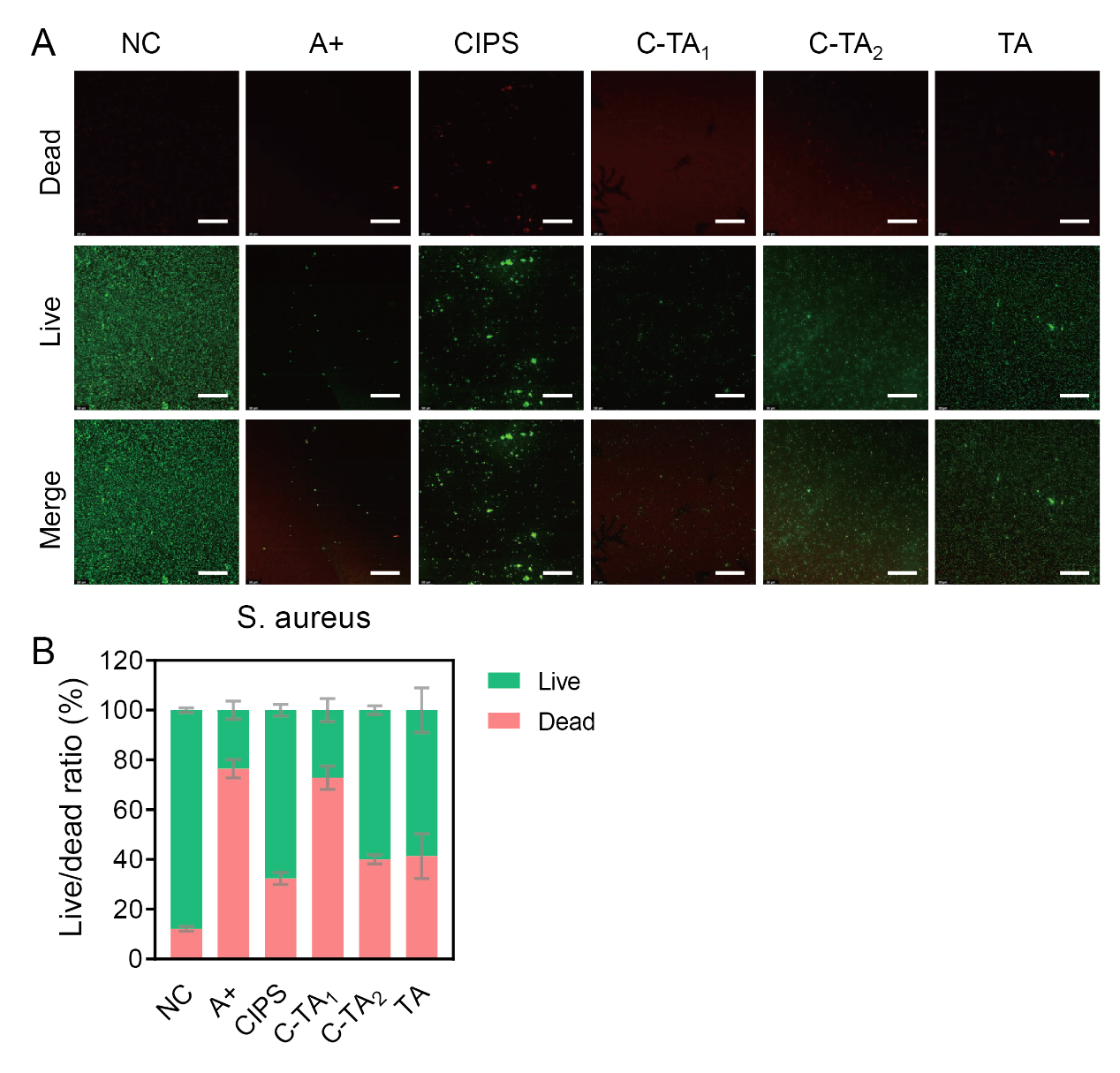


**Figure S33.** (A) Live/dead staining of S. aureus treated with CIPS, C-TA_1_, C-TA_2_, and TA. Scale bars: 100 μm. (B) Quantitative analysis of fluorescence in (A). Data are presented as mean ± SEM (n = 3).


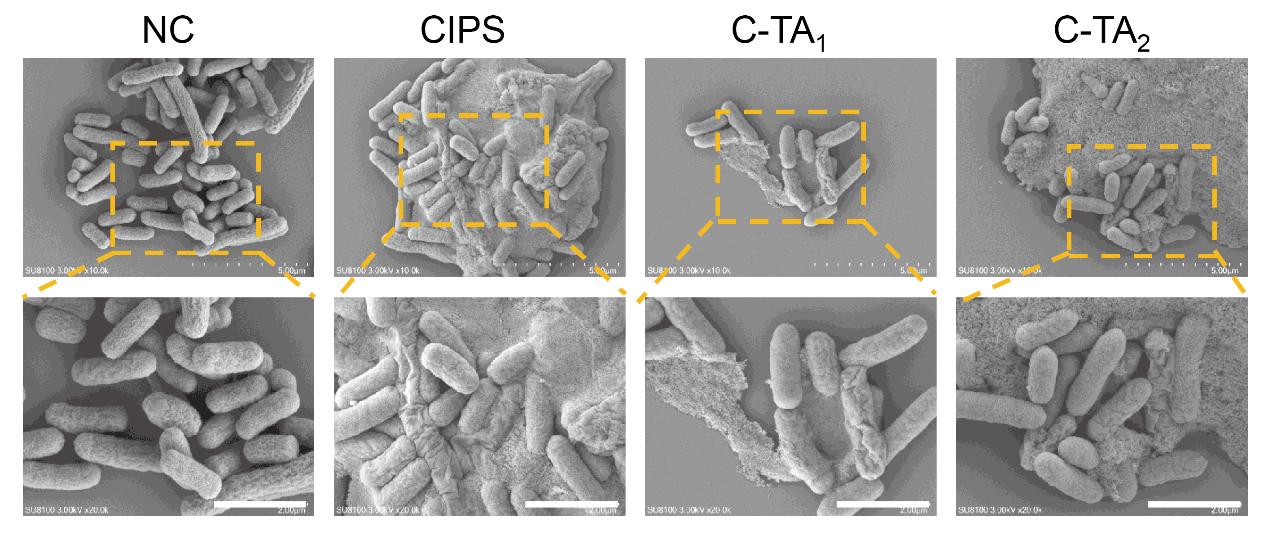


**Figure S34.** Representative SEM images of E. coli treated with CIPS, C-TA_1_, and C-TA_2_. Scale bar (down) = 2 μm.


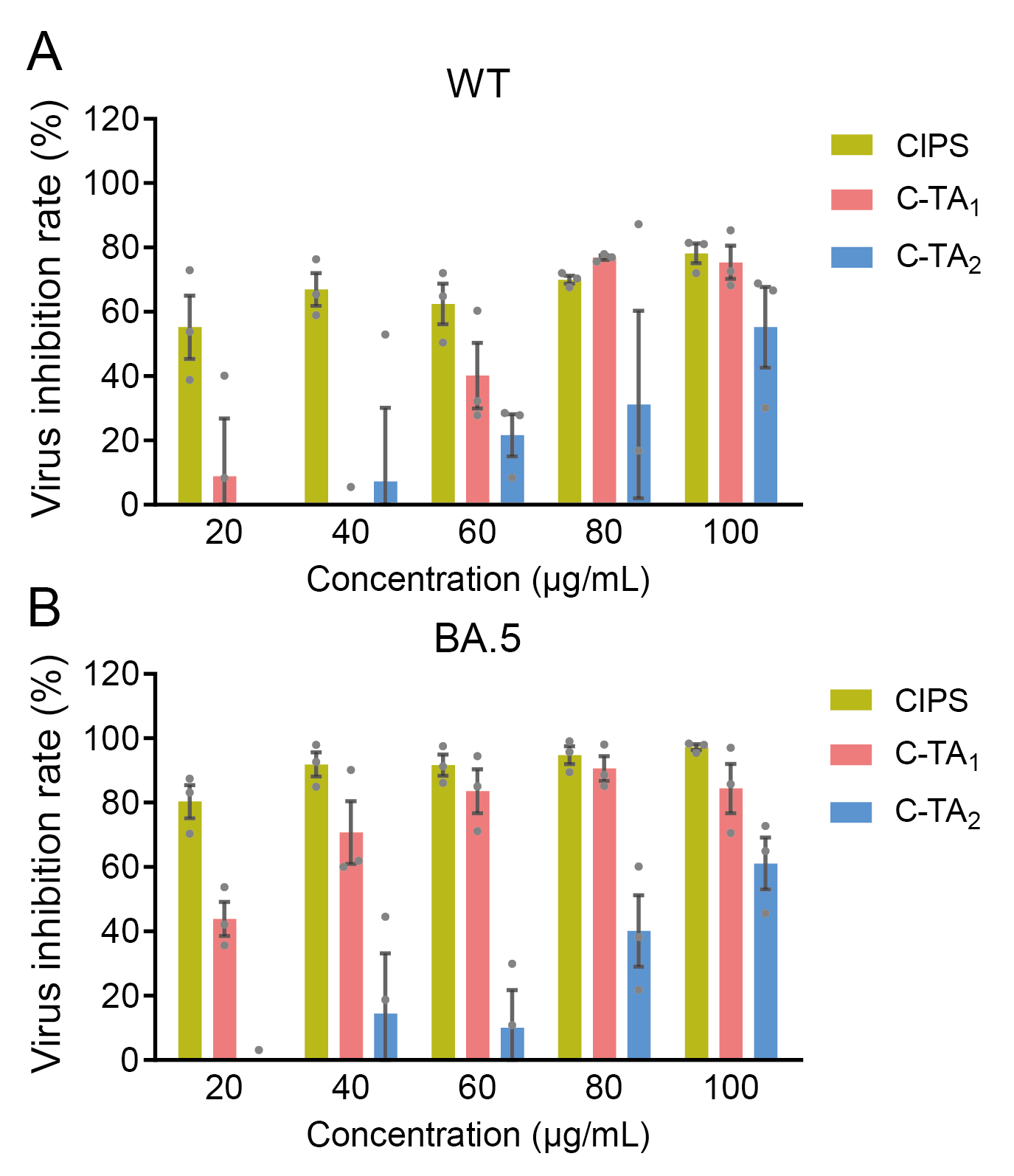


**Figure S35.** Antiviral effect of post-infection ACE2-293 cells treated with CIPS, C-TA_1_, and C-TA_2_ against (A) SARS-CoV-2 WT and (B) SARS-CoV-2 omicron BA.5. Data are presented as mean ± SEM (n = 3).


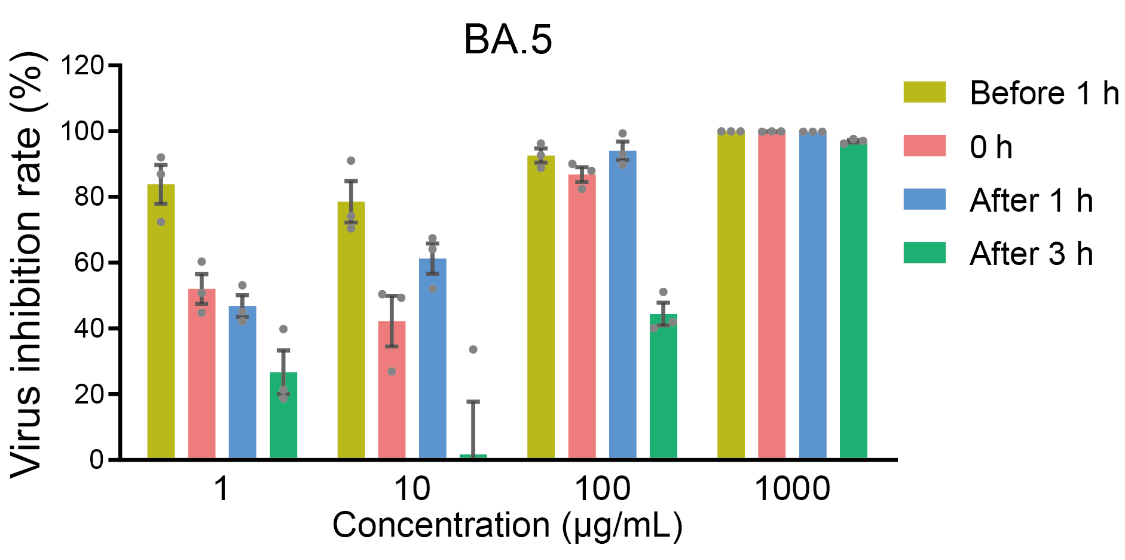


**Figure S36.** Antiviral effect for ACE2-293 cells treated with CIPS with a series of concentrations (1 μg/mL to 1000 μg/mL). CIPS was added to the cells at different times after infection. Data are presented as mean ± SEM (n = 3).


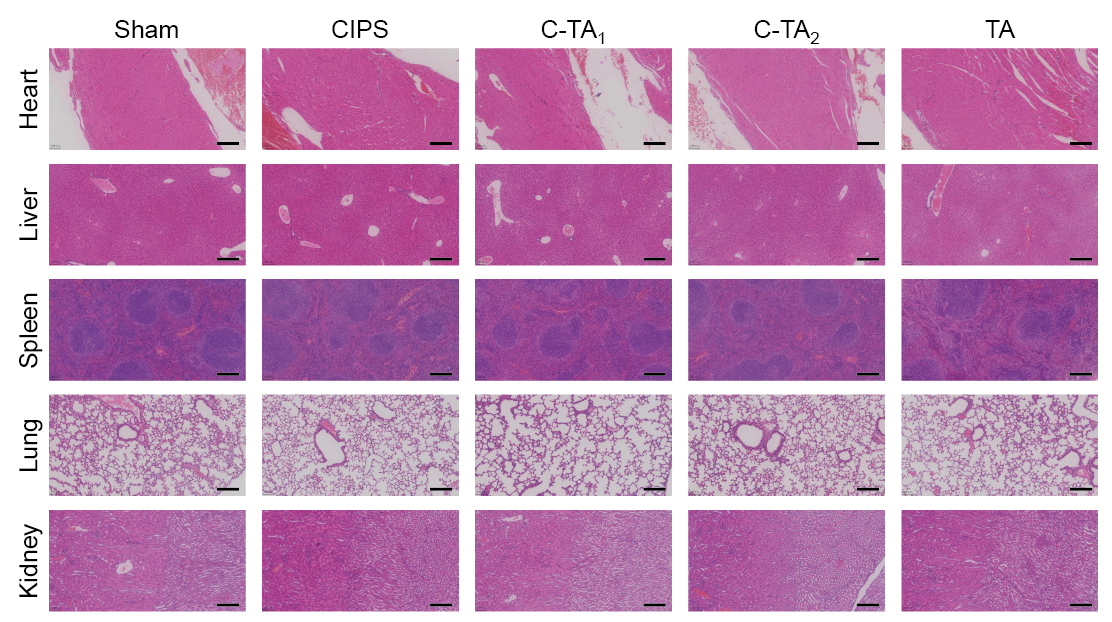


**Figure S37.** Biocompatibility teats of CIPS, C-TA_1_, and C-TA_2_ in vivo. Representative H&E staining images of hearts, livers, spleens, and kidneys of experimental mice. Scale bars: 200 μm.


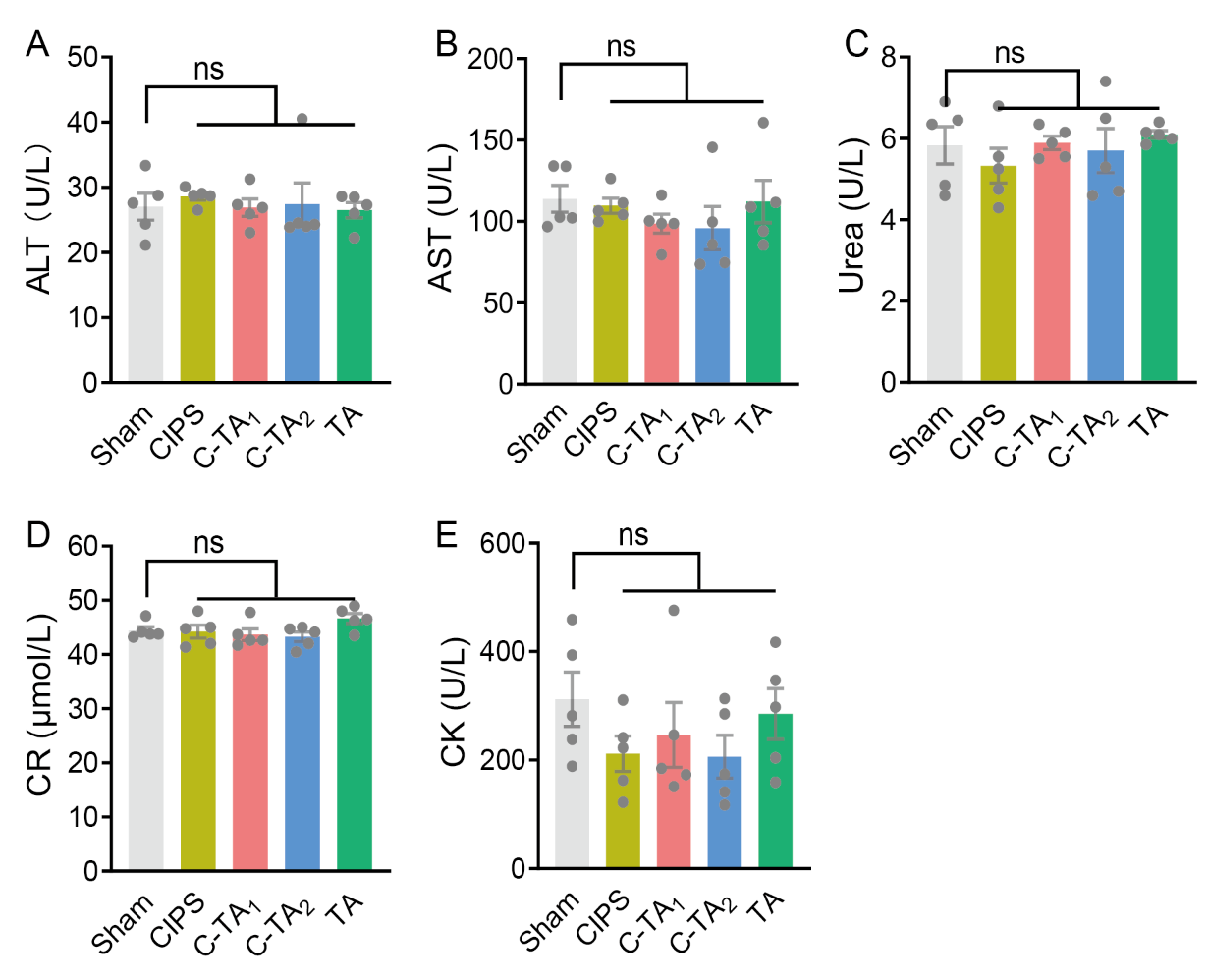


**Figure S38.** (A) ALT, (B) AST, (C) UREA (D) CREA, and (E) CK levels in the serum of mice in each experimental group. Data are presented as mean ± SEM (n = 5, Student’s t-test, two-tailed, ns: no significant difference).


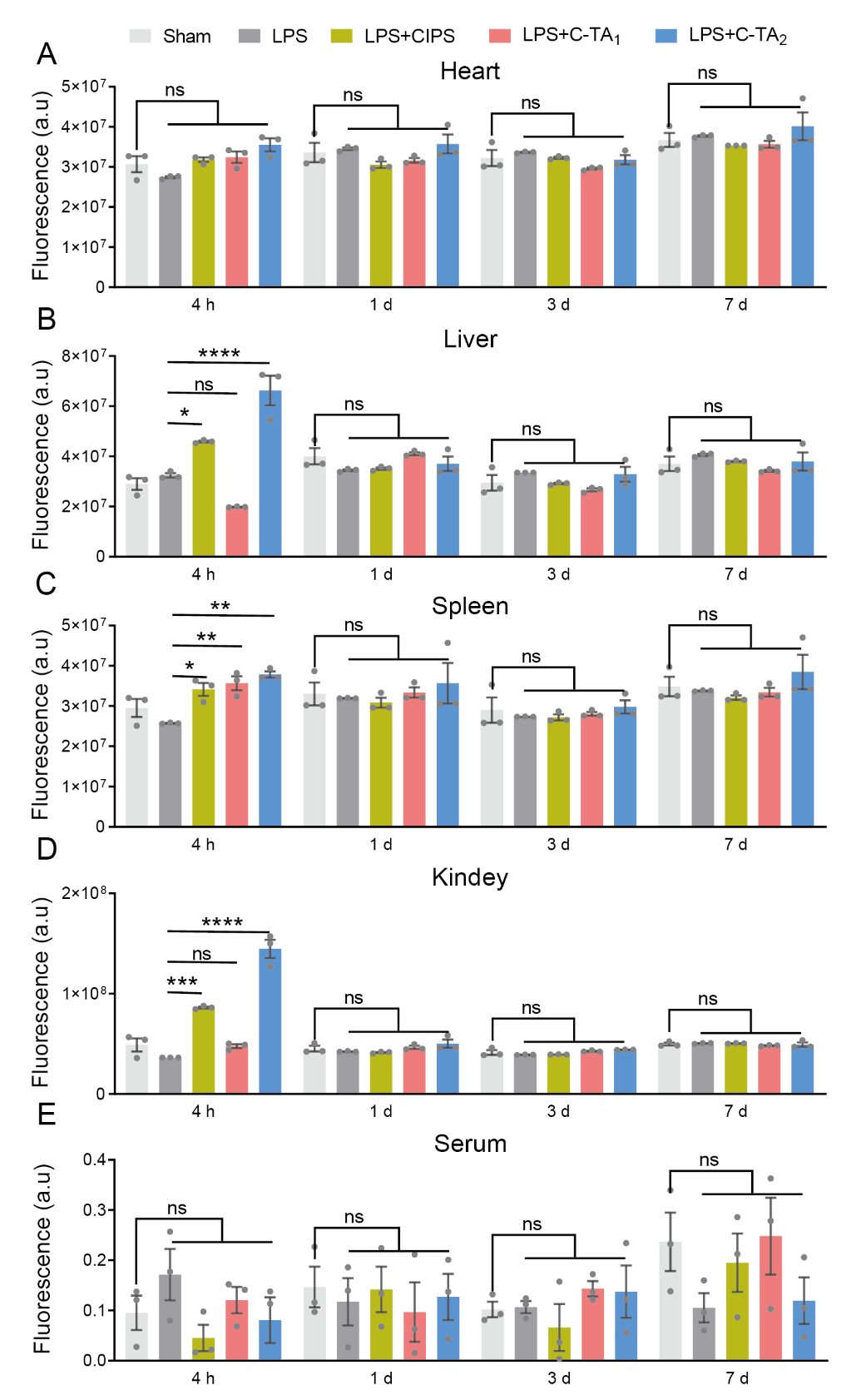


**Figure S39.** Quantification of fluorescence intensities from (A) heart, (B) liver, (C) spleen, (D) kidney, and (E) serum. Data are presented as mean ± SEM (n = 3, Student’s t-test, two-tailed, ns: no significant difference, * *P*<0.05, ** *P*<0.01, *** *P*<0.001, **** *P*<0.0001).


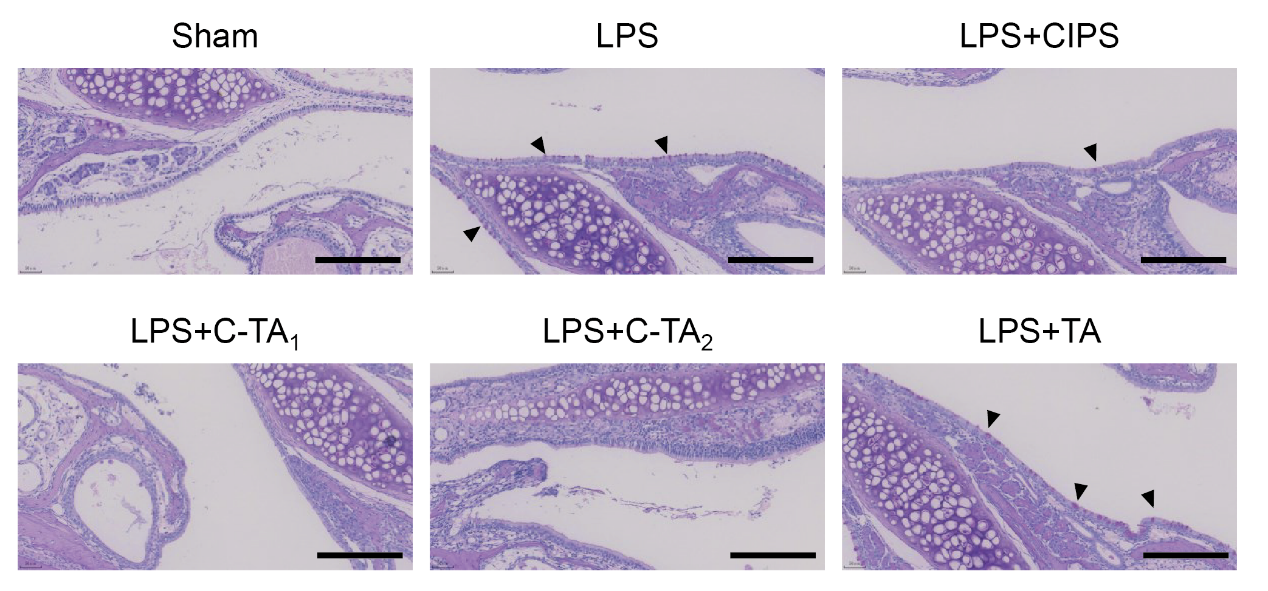


**Figure S40.** PAS staining of the nasal mucosa of experimental mice after different treatments. Scale bars: 200 μm.


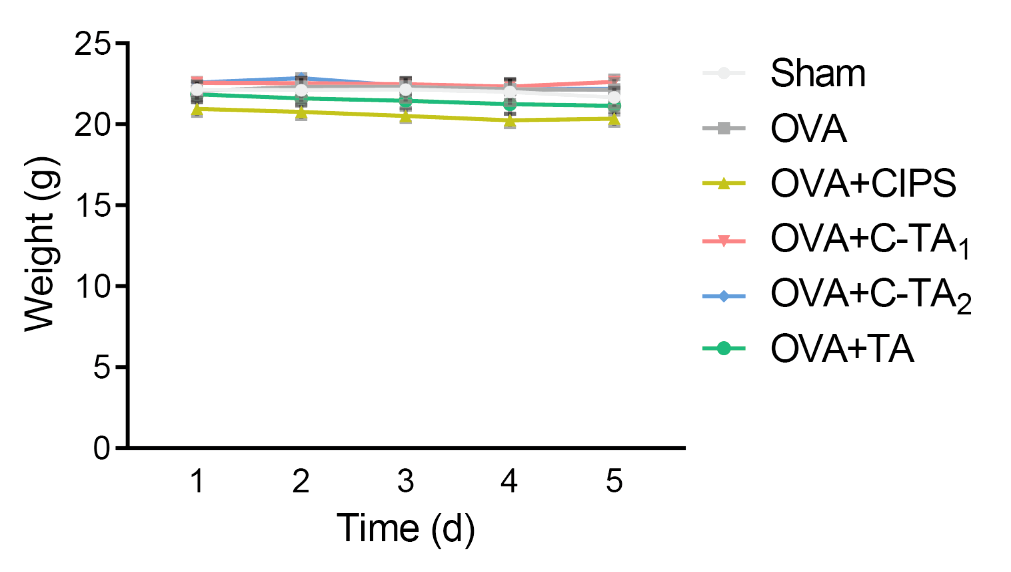


**Figure S41.** The body weight of mice in each group. Data are presented as mean ± SEM (n = 5)


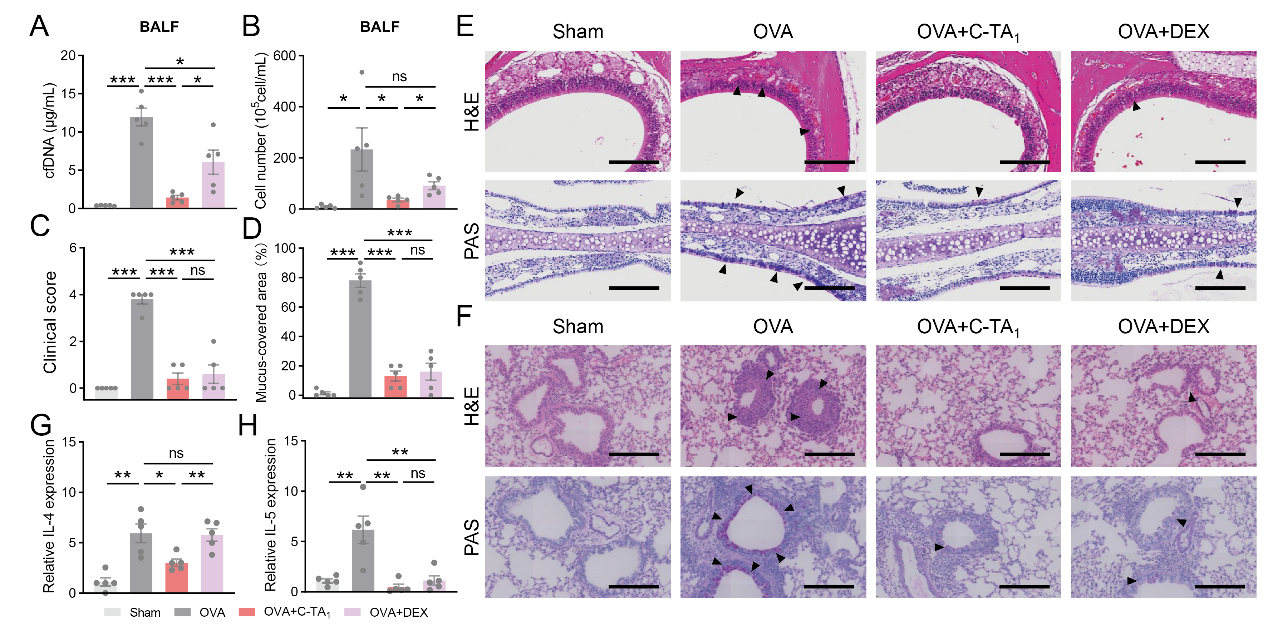


**Figure S42.** **Comparison of the therapeutic results between C-TA_1_ and DEX.** (A) cfDNA levels in BALF of experimental mice. (B) Cell counting in the BALF of mice in each group. (C) Clinical scores based on H&E-staining lung sections. (D) Quantification of mucus formation in lung PAS staining for each group. (E) H&E staining and PAS staining images of the nasal mucosa from experimental mice. (F) H&E staining and PAS staining images of lungs from experimental mice. Scale bars: 200 μm. (G-H) RT-qPCR analysis of IL-4 (G) and IL-5 (H) expression in the lungs of mice in each group. Data are presented as mean ± SEM (n = 5, Student’s t-test, two-tailed, ns: no significant difference, * P<0.05, ** P<0.01, *** P<0.001).


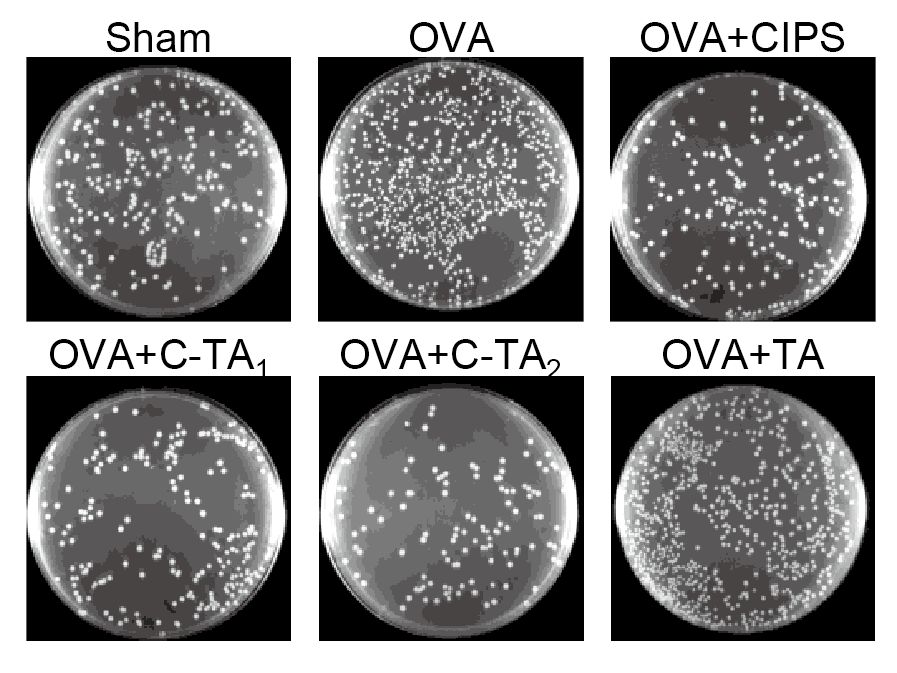


**Figure S43.** BALF bacterial plate cultured in each group.


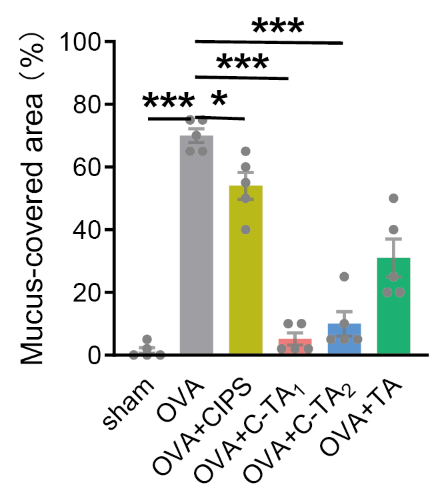


**Figure S44.** Quantification of mucous formation in lung PAS straining in each group. Data are presented as mean ± SEM (n = 5, Student’s t-test, two-tailed, * *P*<0.05, *** *P*<0.001).


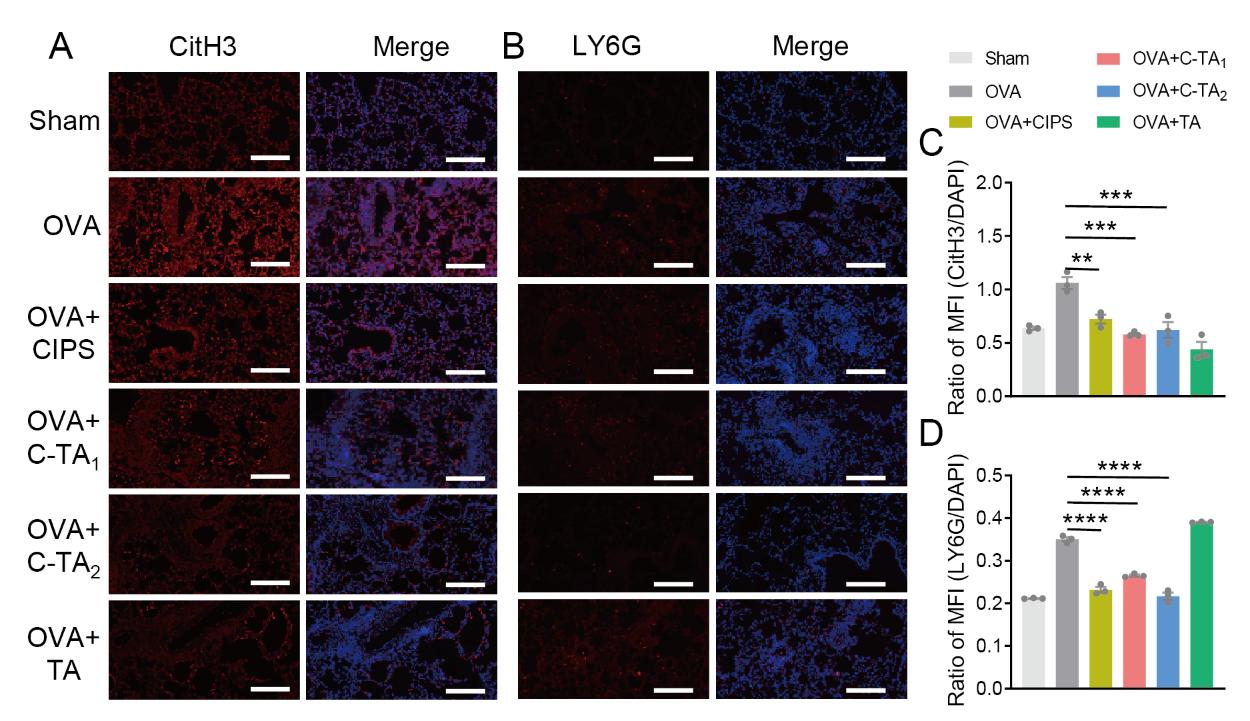


**Figure S45.** (A) Representative DAPI and CitH3 co-staining fluorescent images of lungs from experimental mice. (B) Representative DAPI and LY6G co-staining fluorescent images of lungs from experimental mice. (C) Quantitative analysis of fluorescence in (A). (D) Quantitative analysis of fluorescence in (B). Data are presented as mean ± SEM (n = 3, Student’s t-test, two-tailed, ** *P*<0.01, *** *P*<0.001, **** *P*<0.0001).


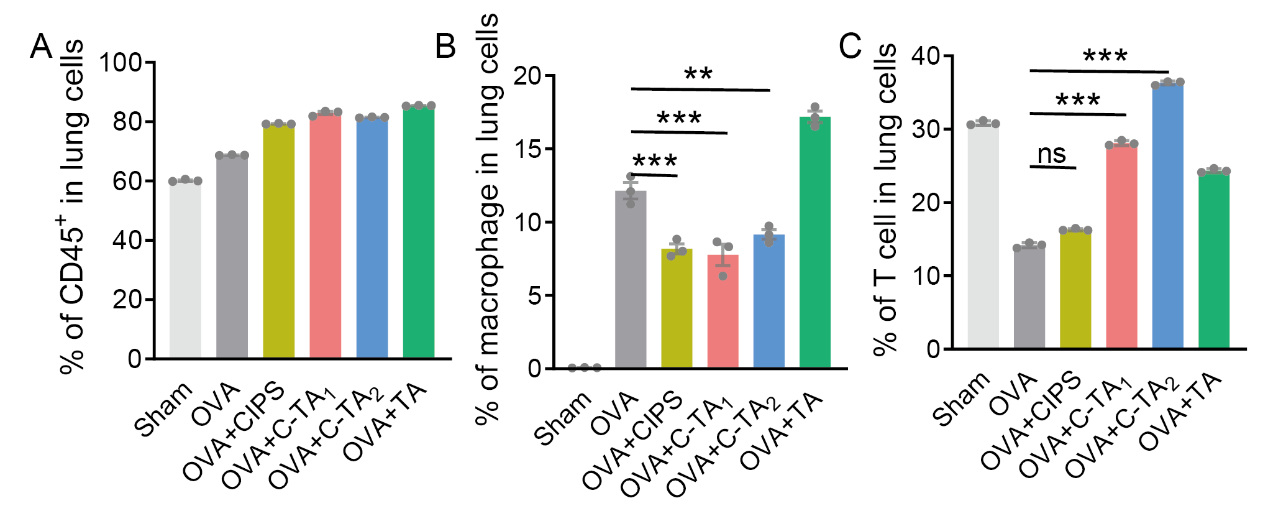


**Figure S46.** Percentage of (A) CD45^+^ cells, (B) macrophages, and (C) T cells in total lung cells calculated by the results of multi-channel flow cytometry. Data are presented as mean ± SEM (n = 3, Student’s t-test, two-tailed, ** *P*<0.01, *** *P*<0.001).


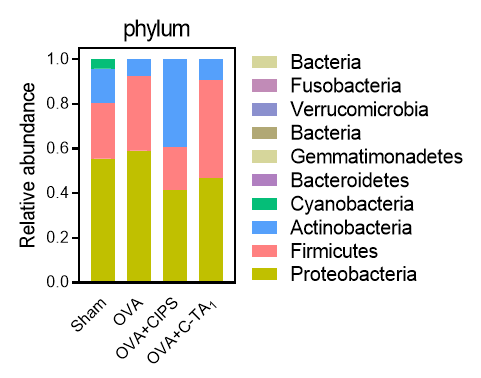


**Figure S47.** Histogram of the relative abundance of each group at the phylum level for the microbiota of BALF from experimental mice.


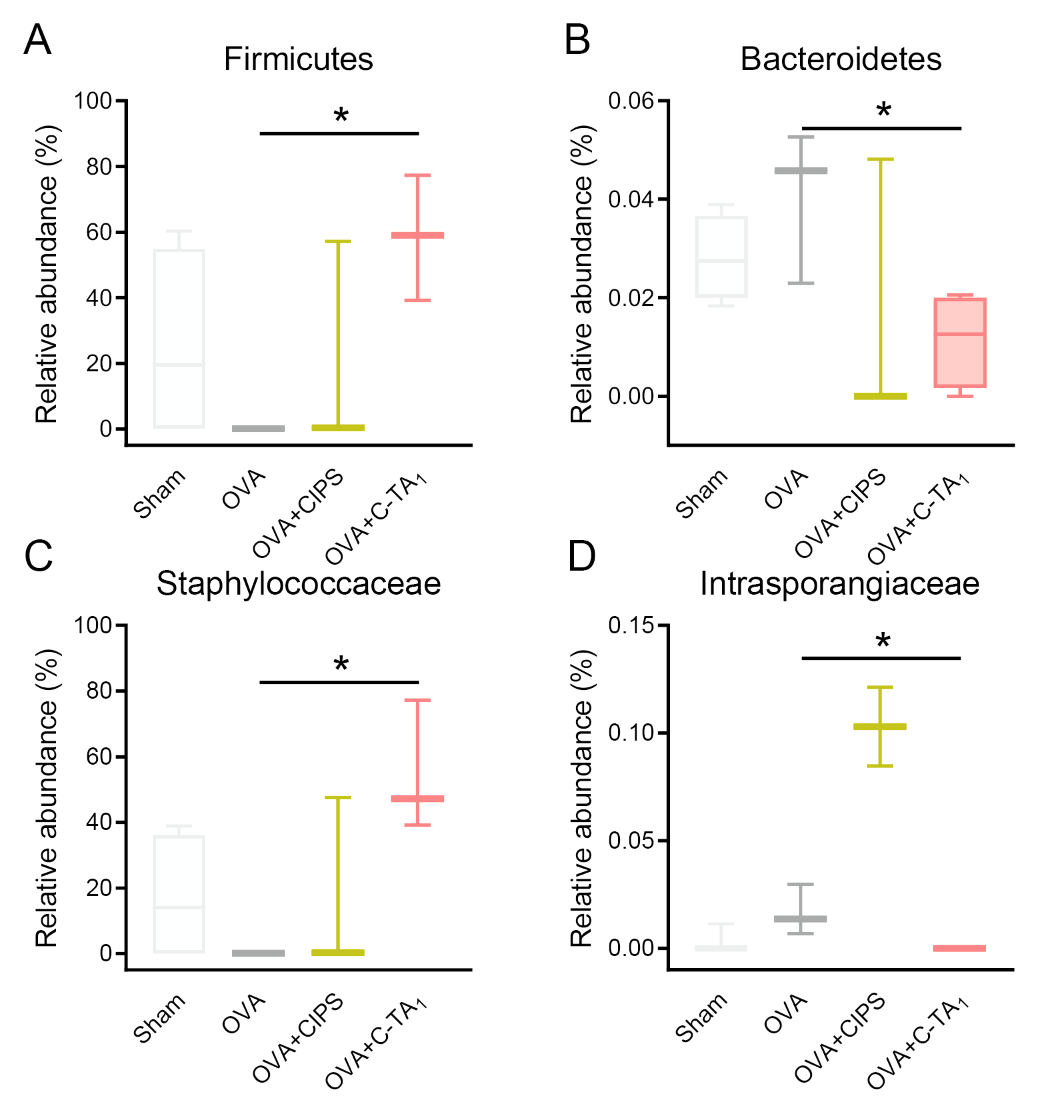


**Figure S48.** Relative abundance of (A) *Firmicutes*, (B) Bacteroidetes, (C) *Staphylococcaceae*, and (D) *Intrasporangiaceae* for the microbiota of BALF from experimental mice*.* Data are presented as mean ± SEM (n = 3, Student’s t-test, two-tailed, * *P*<0.05).


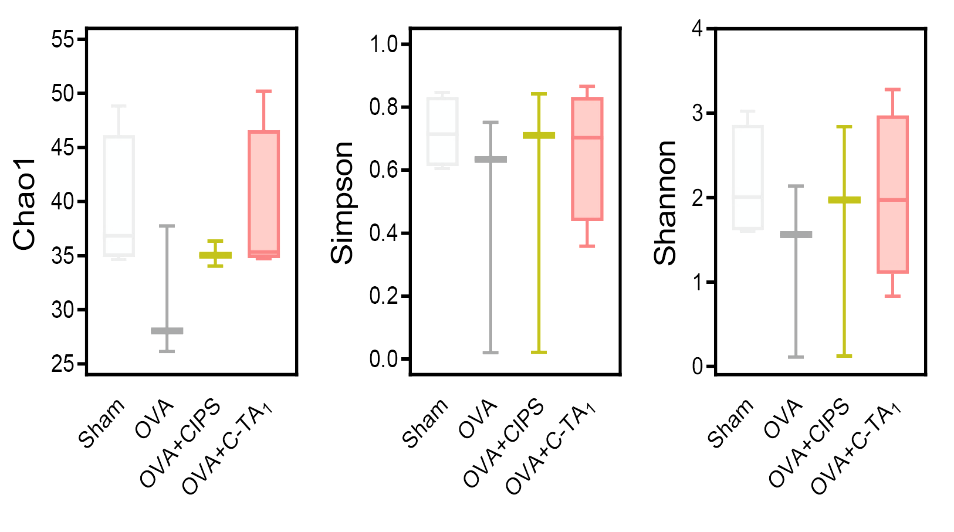


**Figure S49.** Analysis of 𝛼-diversity of oral mucosa microbiome by Chao1, Shannon, and Simpson analyses.


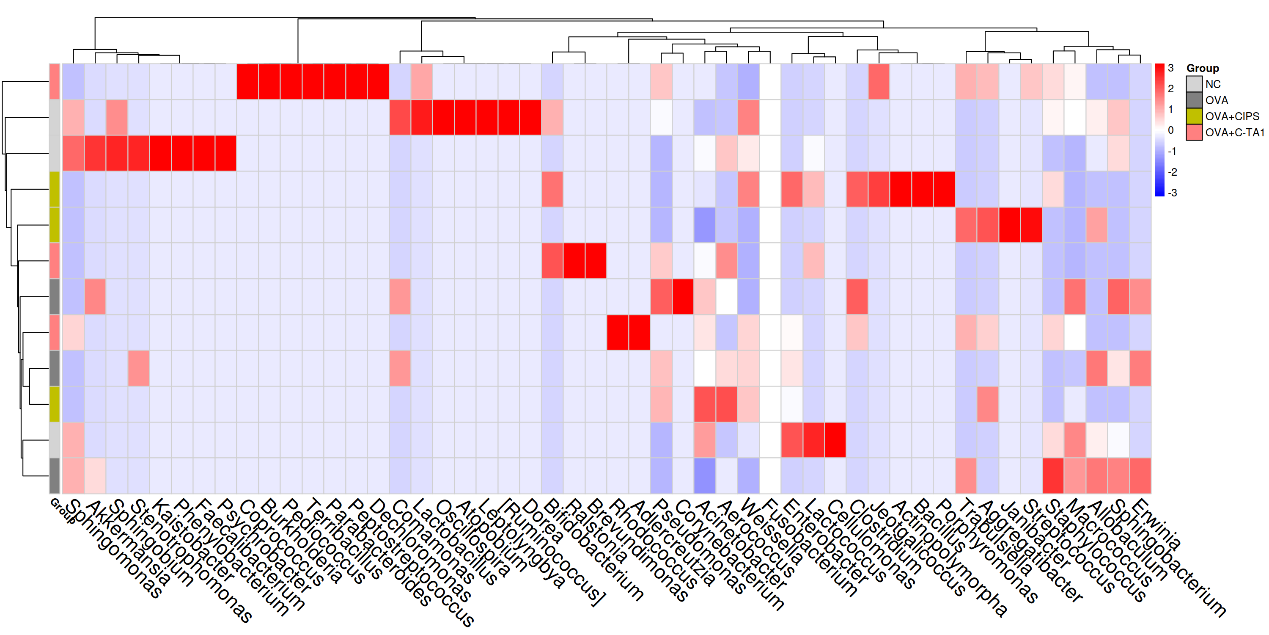


**Figure S50.** Heatmap of the relative abundance of genus-level taxa with dual clustering for the microbiota in the BALF of experimental mice.
